# Global assessment of landscape pattern changes from 1992 to 2020

**DOI:** 10.1007/s10980-025-02210-0

**Published:** 2025-10-16

**Authors:** Tamsin L. Woodman, Peter Alexander, David F. R. P. Burslem, Justin M. J. Travis, Karina Winkler, Felix Eigenbrod

**Affiliations:** 1 School of Biological Sciences, Zoology Building, University of Aberdeen, Tillydrone Avenue, Aberdeen, AB24 2TZ Scotland; 2 School of GeoSciences, University of Edinburgh, Drummond Street, Edinburgh, EH8 9XP UK; 3 Global Academy of Agriculture and Food Systems, University of Edinburgh, Edinburgh, EH25 9RG UK; 4 Land Use Change and Climate, IMKIFU, Karlsruhe Institute of Technology (KIT), Campus Alpin, Garmisch-Partenkirchen, Germany; 5 School of Geography and Environmental Science, University of Southampton, Highfield Campus, Southampton, SO17 1BJ UK

**Keywords:** Landscape patterns, Landscape metrics, Land use and land cover change, Global spatial scale, Landscape fragmentation

## Abstract

**Context:**

Changes in landscape patterns, which refer to the composition and spatial configuration of land use and land cover (LULC) classes in a landscape, can have negative impacts on biodiversity and environmental processes such as carbon cycles. Such impacts are both dependent on the spatial extent of changes and which LULC classes are affected, but previous global-scale landscape pattern assessments have focused on single LULC classes or landscape-level measurements only. A comprehensive, multiscale analysis across multiple LULC types is therefore key for understanding the full impact of landscape pattern change on the environment.

**Objectives:**

We assessed global-scale change in landscape patterns for six LULC classes from the HILDA+ dataset (urban, cropland, pasture/rangeland, forest, unmanaged grass/shrubland, and sparse/no vegetation) between 1992 and 2020.

**Methods:**

Six class-level landscape metrics with predictable scaling behaviour across landscape extents were calculated at global scale for each LULC class and year. Landscape metrics were quantified for five landscape extents (100, 400, 1600, 6400 and 25,600 km^2^). Trends in landscape metrics were evaluated and linked to changes in LULC composition (area) and configuration over time.

**Results:**

Unmanaged grass/shrubland LULC expanded in area and showed increased number of patches, edge length, and complexity in shapes, while pasture/rangeland and forest LULC tended to decline in area, number of patches, and edge length. Even though there was high spatial heterogeneity in landscape pattern change for all LULC classes, neighbouring 100 km^2^ landscapes often showed the same directional change in area and fragmentation.

**Conclusions:**

Global landscape pattern change was highly variable for all LULC classes between 1992 and 2020, suggesting that drivers of LULC change act on local to regional scales. We expect that the multiscale global dataset of landscape metrics generated here will have future applications in understanding the drivers of landscape pattern change and its environmental impacts.

**Supplementary Information:**

The online version contains supplementary material available at 10.1007/s10980-025-02210-0.

## Introduction

Understanding how global landscape patterns have fluctuated through time is key for deciphering the drivers of changes and how these changes impact the Earth System. Anthropogenic land use and land cover (LULC) change is driving the loss and fragmentation of the Earth’s remaining natural ecosystems, with potentially damaging consequences for the Earth System (Haddad et al. 2015). Approximately 2.3 million km^2^ of forest was lost between 2000 and 2012 (Hansen et al. 2013), for example, and over 70% of remaining forest cover is within 1 km of a forest edge (Haddad et al. 2015). By contrast, the amount of cropland has increased 9% from 2003 to 2019 (Potapov et al. 2021). LULC change alone can have adverse effects on the environment, such as through driving biodiversity loss (Maxwell et al. 2016; Jaureguiberry et al. 2022). Changes in landscape patterns can also have negative environmental impacts; for example, the increasing amount of tropical forest edge habitat has been estimated to release 0.34 Gt of carbon per year (Brinck et al. 2017). Moreover, landscape patterns are important for a range of environmental processes, including the movement of organisms (Fischer and Lindenmayer 2007), fire spread (Ryu et al. 2007) and ignition (Pais et al. 2021), and accumulation of soil carbon (Liu et al. 2022). Therefore, knowledge of how LULC has changed historically in terms of both area and pattern is important for estimating the impacts of LULC change on the Earth System.

‘Landscape patterns’ encompass both the amount (composition) and configuration (spatial arrangement) of LULC classes within a landscape (Gustafson 1998, 2019). LULC change alters landscape patterns by changing both the composition (area) and configuration of LULC classes. For natural land cover types, such changes in LULC are often characterized as habitat loss and fragmentation. Habitat loss always refers to changes in composition, while fragmentation can refer to the process of the splitting apart of a LULC class into more, smaller patches with greater edge length (which affects both composition and configuration), or to changes in configuration independently of composition (fragmentation per se; Fahrig 2003).

The drivers of landscape pattern change and the impact of landscape patterns on the environment may differ across scales (Ewers and Laurance 2006; Cattarino et al. 2014; Jin et al. 2023), hence the choice of scale on which to study the effects of landscape patterns can have considerable influence on the conclusions from a study (Turner 1989; Miguet et al. 2016). For example, indices of macroinvertebrate richness were most closely associated with landscape patterns calculated at a scale of 200 m-wide riparian corridors (Sponseller et al. 2008), suggesting that this scale would be most appropriate for studying the effects of landscape patterns on macroinvertebrate diversity. Similarly, the relationships between landscape patterns and plant diversity are strongly dependent on the scale at which landscape patterns are quantified (Martello et al. 2023; Jin et al. 2023). The choice of scale on which landscape metrics, which are used to quantify landscape patterns (Gustafson 1998, 2019), are calculated is therefore important as it may affect how landscape patterns appear to relate to their drivers and to environmental processes. Calculating landscape metrics at a range of scales may be preferable to better understand their cross-scale associations with landscape pattern drivers and environmental processes (Miguet et al. 2016).

Although it is important to study landscape patterns at multiple scales, there are relatively few landscape metrics that behave predictably as the scale of a landscape changes in terms of both extent and resolution (scale invariant behaviour; Turner 1989; Wu et al. 2002; Wu 2004; Uuemaa et al. 2005; Argañaraz and Entraigas 2014). The scale-sensitive behaviour of most landscape metrics may confound the relationships between metrics and environmental processes (Frazier and Kedron 2017). Therefore, using scale-sensitive landscape metrics might impede our ability to (a) identify the scale on which landscape metrics are most relevant for environmental processes and (b) make cross-scale comparisons of the drivers and impacts of landscape patterns. An increasing number of landscape metrics have become available in recent years compared to those tested for scaling relationships in previous studies (Wu et al. 2002; Wu 2004; Šímová and Gdulová 2012), which suggests there is an opportunity to assess the scaling behaviour of these new landscape metrics and update our knowledge of the behaviour of landscape metrics across scales.

The choice of landscape metrics used to assess landscape patterns at a global scale has varied between studies, making it hard to compare studies that have focused on a single LULC class or landscape extent (e.g., Haddad et al. 2015; Hu et al. 2020; Ma et al. 2023). To our knowledge, previous estimations of global landscape pattern change have focused on forest or cropland patterns only (Riitters et al. 2000; Haddad et al. 2015; Hu et al. 2020; Ma et al. 2023), or quantified change at the level of entire landscapes rather than for individual LULC classes (Jacobson et al. 2019). Moreover, few studies have assessed global-scale landscape patterns across scales, except for Riitters et al. (2000) who calculated global forest fragmentation across four spatial scales for a single time point. Although natural land cover classes such as forest are thought to have decreased and become more fragmented through time (Hansen et al. 2013; Haddad et al. 2015; Jacobson et al. 2019), a more recent study indicated that the majority of forested landscapes across the globe may have exhibited a trend of declining fragmentation between 2000 and 2020 (Ma et al. 2023). However, the metrics used to quantify landscape patterns differ between studies; for instance, Haddad et al. (2015) utilised distance to edge, number of fragments and fragment area to quantify global forest fragmentation, whereas Ma et al. (2023) employed a fragmentation index constructed from edge density, patch density, and mean patch area. Consequently, there is a need for global-scale landscape pattern change assessments that encompass multiple LULC classes and landscape extents to give a better understanding of how landscape patterns are changing over time in terms of both area and configuration.

This study aims to quantify global-scale landscape patterns for several LULC classes and landscape extents using scale invariant landscape metrics, and assess how landscape patterns have changed over recent decades. This is the first study to calculate landscape metrics at global scale for multiple LULC classes, landscape extents, and years. Given that most class-level landscape metrics show unpredictable scaling relationships across landscape extents (Wu 2004), we first use a single country, Colombia, which has highly heterogeneous landscape patterns, to identify metrics with predictable scaling relationships when landscape extent increases. The selected metrics are then used to calculate landscape metrics globally for a range of landscape extents between 1992 and 2020, to address how global landscape patterns have changed over time. Our study aims to characterise the overall trends and spatial variability in global-scale landscape patterns over the past three decades. We expect that our global dataset of landscape metrics could be used in future to investigate both the drivers of landscape pattern change and the impacts of these changes on environmental processes. The inclusion of landscape metrics with predictable behaviour across scales will allow for multiscale studies of the relationships between landscape metrics, drivers of change, and environmental processes.

## Methods

### Land use and land cover data

We used land use and land cover (LULC) data from the HILDA+ version 2b dataset in the Eckert IV projection for the calculation of landscape metrics at global scale (Winkler et al. 2020, 2021, 2025; Woodman et al. 2025). HILDA+ provides yearly 1 km spatial resolution LULC data from 1960 to 2020. Each grid cell contains a single LULC class which is derived from an aggregation of multiple LULC maps and other related datasets, such as FAO land use statistics (Winkler et al. 2020, 2021, 2025). There are six LULC classes in HILDA+ version 2b: urban, cropland, pasture/rangeland, forest, unmanaged grass/shrubland and sparse/no vegetation. HILDA+ distinguishes between managed pasture/rangelands and unmanaged grass/shrublands, giving it an advantage over other global LULC datasets that treat managed and unmanaged grasslands as the same LULC class. Pasture/rangeland is defined in HILDA+ as managed herbaceous plants with at least 10% cover, including areas that are used for livestock and hay production. Unmanaged grass/shrublands are natural herbaceous plants with at least 10% cover that are not managed by people, including wetland areas. Both the pasture/rangeland and unmanaged grass/shrubland classes include mosaics of herbaceous plants with trees and shrubs. A grid cell must have at least 10% cover of trees that are taller than 5 m to be classed as forest (Winkler et al. 2020, 2021, 2025). Uncertainty in the HILDA+ dataset varies over both space and time, as well as by land use class. Annual per-pixel uncertainty layers are provided with the dataset and are based on the number of available input datasets, the maximum deviation in class area fraction, and the mean class area fraction from all input datasets for each year (Winkler et al. 2020, 2021). Uncertainty is generally higher prior to the 1990s, mainly due to limited availability and lower resolution of input data for earlier years. Spatially, uncertainty is elevated in heterogeneous landscapes, such as the savannahs of Sub-Saharan Africa, where mixtures of managed and unmanaged lands lead to greater disagreement between input datasets. This is particularly relevant for the managed versus unmanaged grass/shrubland classes, which are more difficult to distinguish in regions with complex land management and natural vegetation mosaics. These uncertainties should be considered when interpreting change results, especially for grassland classes (Winkler et al. 2021).

We restricted the study period to between 1992 and 2020 because 1992 is the first year that a high resolution, yearly LULC dataset (the ESA CCI Land Cover time series) is used as input to HILDA+ (ESA 2017; Winkler et al. 2020, 2025). Although uncertainty in the HILDA+ dataset for 1992 is still higher than for 2020, primarily due to the limited availability of high-resolution input data at the beginning of the time series, the continuous coverage provided by ESA CCI Land Cover with 300 m spatial resolution guarantees that there are no data gaps due to missing spatial detail within the 1992–2020 period. For global analysis we used HILDA+ maps in the World Eckert IV projection, which is an equal area projection. These maps were cropped to exclude the continent of Antarctica and sub-Antarctic islands as very little LULC change occurred here during the study period. The HILDA+ LULC maps were cropped in R software version 4.1.3 (R Core Team 2022) using the ‘terra’ R package version 1.7–23 (Hijmans 2022) and an outline of Antarctica from the ‘rnaturalearth’ package version 0.3.2 (Massicotte and South 2023).

### Selection of landscape metrics

A key goal of our study was to generate a dataset of landscape metrics that could be used to a) assess changes in global-scale landscape patterns and b) investigate relationships between landscape metrics, drivers of landscape pattern change, and ecological processes in future. Scale-dependent behaviour of landscape metrics may confound the relationships between metrics and ecological processes (Frazier and Kedron 2017), and might also cause differences in landscape trends across scales that are an artifact of scale-sensitive behaviour. Therefore, we chose to include only landscape metrics with scale invariant behaviour in our global dataset. To achieve this, we first assessed the behaviour of class-level landscape metrics from the ‘landscapemetrics’ R package (Hesselbarth et al. 2019) with increasing landscape extent for Colombia, before moving to global level analyses. There have been large changes in LULC over time in Colombia, and the rate and drivers of LULC change have varied both spatially and temporally. For instance, in the late twentieth century the Andean region experienced the highest rates of deforestation (Etter et al. 2008). Current drivers of LULC change in Colombia include clearing of forests for cattle grazing, legal and illegal crop production, mining, and urbanization (Etter et al. 2008; Armenteras et al. 2011; González-González et al. 2021). Given its large area, diverse land covers and varied drivers of LULC change, all six LULC classes from HILDA+ were represented in Colombia (Winkler et al. 2020, 2021, 2025; Woodman et al. 2023). Colombia was therefore considered a suitable case study to test the scaling relationships of landscape metrics across ten landscape extents (100, 400, 900, 1600, 2500, 3600, 4900, 6400, 8100 and 10,000 km^2^).

**Fig. 1:**
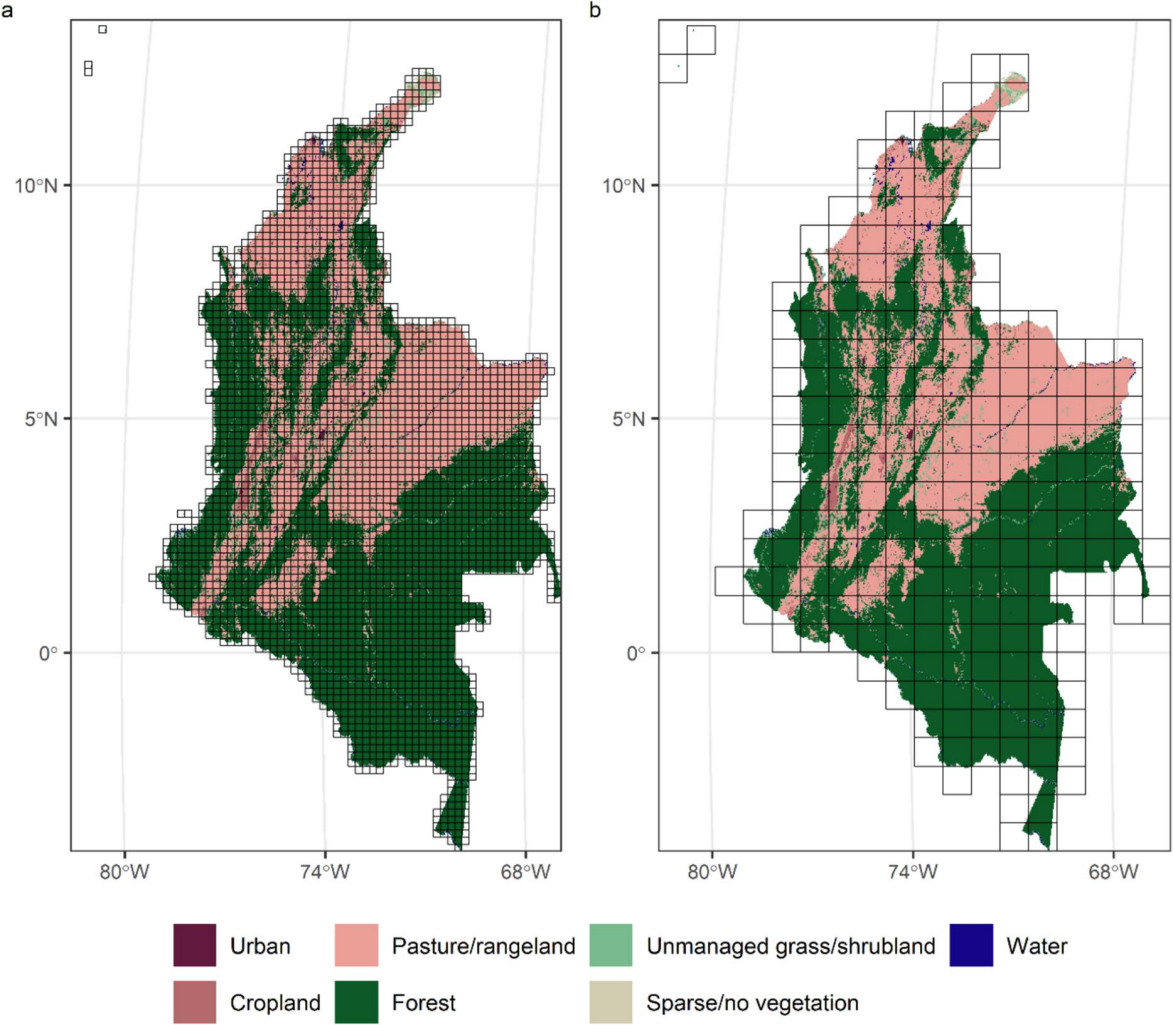
Example landscapes for Colombia with extents of **a** 400 km^2^ and** b** 6400 km.^2^. Landscapes are depicted using black lines and are overlaid on land use and land cover maps from HILDA+ in 1992

First, an outline of Colombia from the ‘rnaturalearth’ version 0.3.2 package (Massicotte and South 2023) was used to crop the HILDA+ dataset from 1992 to 2020 to the same extent as Colombia. Next, a set of ten regular grids covering the terrestrial surface of Colombia were created to represent landscapes with different extents. Each grid had landscapes (grid cells) with sides of between 10 and 100 km length at 10 km increments, giving a total of ten grids. Each grid was overlaid with LULC in Colombia from HILDA+ and all grid cells that were entirely classified as ocean by HILDA+ in every year from 1992 to 2020 were removed. Each individual cell in a grid was treated as a landscape for the calculation of landscape metrics. Two examples of landscapes with different extents (sides of length 20 km and 80 km, or 400 km^2^ and 6400 km^2^ landscape extent, respectively) overlaid on LULC in Colombia in 1992 are shown in Fig. 1. Next, all 55 class-level landscape metrics implemented in the ‘landscapemetrics’ R package version 1.5.6 (Hesselbarth et al. 2019) were calculated in the first year of the study period (1992) for each landscape in the ten grids.

After calculating class-level landscape metrics using the ‘landscapemetrics’ R package, we analysed the scaling relationship between the mean of each landscape metric and landscape extent. First, we removed the two HILDA+ water LULC classes (ocean and water) from the dataset of landscape metrics in Colombia in 1992. The ‘landscapemetrics’ R package does not return a value for class-level landscape metrics when a LULC class is not present in a landscape (Hesselbarth et al. 2019), which resulted in missing values for metrics in many landscapes. It is not possible to calculate a value for the majority of landscape metrics when a LULC class is not present in a landscape, so we treated missing values for these metrics as missing when summarising the dataset (Table S1). However, for other metrics, such as class area (CA) and total edge length (TE), the value of the metric is equivalent to zero when a LULC class is not present in a landscape. Therefore, we replaced missing values with zeros for eleven out of the 55 landscape metrics available in the ‘landscapemetrics’ package (Hesselbarth et al. 2019).

The mean of each landscape metric was then calculated across all landscapes for every landscape extent, and the mean of landscape metrics as predicted by landscape extent in terms of the length of each landscape in kilometres was plotted (as in Wu 2004). The resulting plots were examined to establish which landscape metrics showed consistent scaling relationships as landscape extent increased. Wu (2004) identified five class-level landscape metrics that behaved predictably with increasing landscape extent, and these metrics had either a power law or linear relationship with landscape extent. Therefore, we selected landscape metrics as having consistent scaling relationships if they showed a smooth linear or power law relationship with increasing landscape extent for all LULC classes on visual inspection. Landscape metrics where the relationship with extent differed between LULC classes, such as effective mesh size (positive relationship with extent for forest and pasture/rangeland, negative relationship for urban LULC) were excluded from further analysis. Similarly, there were several metrics (e.g., Largest Patch Index and mean of patch area) that exhibited a change in the direction of the relationship with landscape extent at larger extents. These metrics were considered to have unpredictable scaling relationships. Overall, six landscape metrics were found to have predictable scaling behaviour as landscape extent increased (Figs. S 1 and S 2).

The six landscape metrics that demonstrated predictable scaling with landscape extent across Colombia were: class area (CA), Landscape Shape Index (LSI), number of disjunct core area patches (NDCA), number of patches (NP), total core area (TCA), and total edge length (TE). Four of these six metrics were previously identified as having consistent behaviour as landscape extent was increased across a set of landscapes in the United States (Wu 2004). CA is the total area of a LULC class in one landscape in square kilometres, TE is the total edge length of a LULC class in a landscape in kilometres, and NP gives the number of non-contiguous patches of a LULC class. NDCA and TCA are both core area metrics, where ‘core area’ consists of grid cells that are surrounded by cells of the same class. NDCA counts the number of non-contiguous core area patches for a LULC class within a landscape, meaning it is a measure of the number of ‘patches within patches’. Meanwhile, TCA is the total area of a LULC class that can be considered as the core area within a landscape, with units of square kilometres. LSI is calculated as a ratio of the total edge length of a LULC class to the hypothetical minimum edge length of that class if it was as aggregated as possible (Hesselbarth et al. 2019).

### Calculating landscape metrics at global scale

**Fig. 2:**
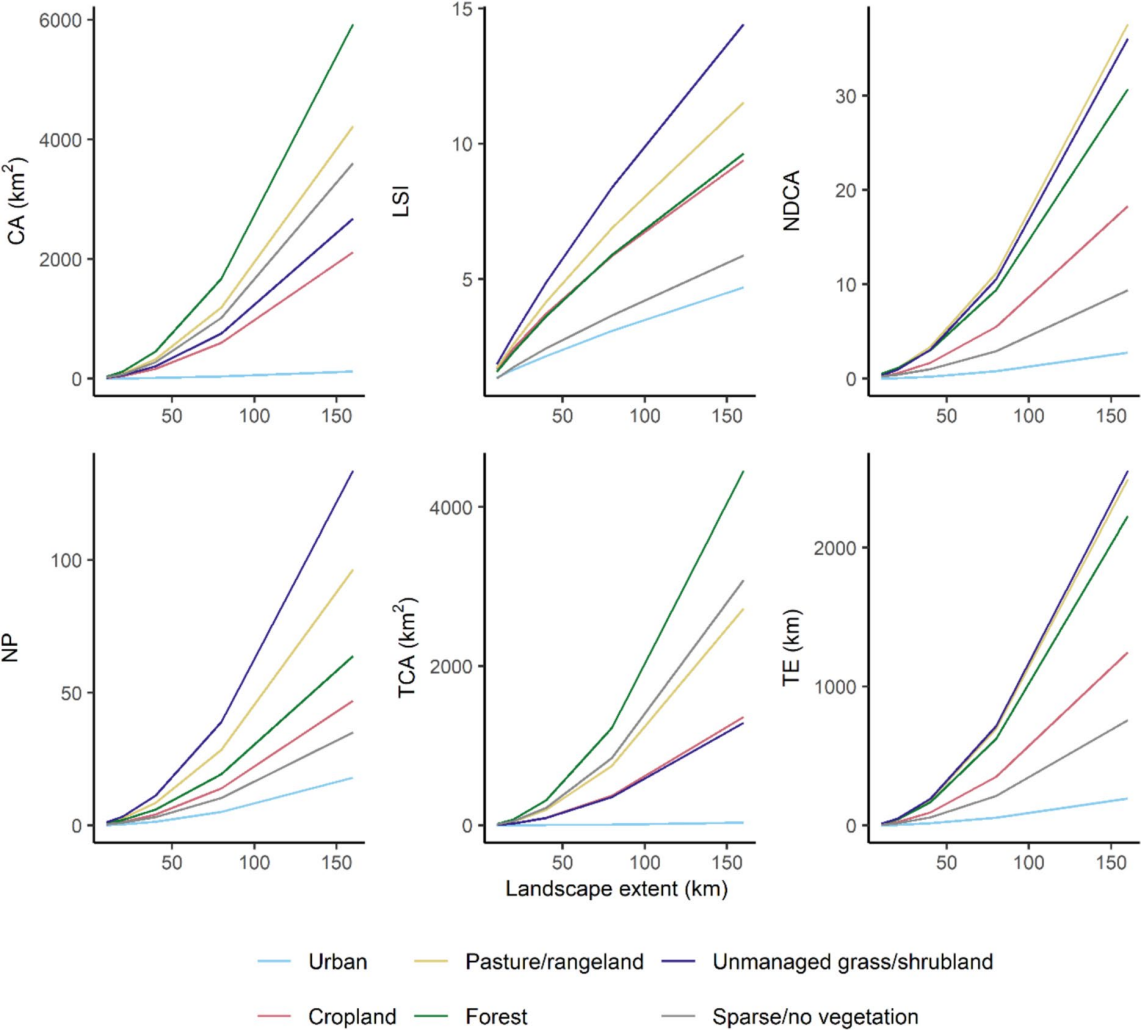
Scaling relationships for six landscape metrics at global scale. Lines give the mean value of a metric across landscapes of different extents for one LULC class. Landscape extent is the length of each side of a landscape in kilometres. CA = class area (km^2^), LSI = Landscape Shape Index, NDCA = number of disjunct core area patches, NP = number of patches, TCA = total core area (km^2^), TE = total edge length (km)

The six landscape metrics that showed consistent scaling relationships with increasing landscape extent across Colombia in 1992 were calculated at global scale to create a cohesive dataset of landscape metrics for multiple LULC classes and landscape extents. We decided to calculate global landscape metrics for five landscape extents: 10 by 10 km, 20 by 20 km, 40 by 40 km, 80 by 80 km, and 160 by 160 km (100, 400, 1600, 6400 and 25,600 km^2^, respectively). The 10 by 10 km (i.e., 100 km^2^) extent was chosen because it approximately matches the resolution of available socioeconomic datasets (e.g.: Center for International Earth Science Information Network—CIESIN—Columbia University 2018; Kummu et al. 2018; Fischer et al. 2021) that could be utilised in future to investigate drivers of landscape patterns. The further four landscape extents were selected by doubling the number of kilometres per side of a landscape. Most global land use models typically generate projections at coarse resolutions (e.g., in regions or 0.5° grids; Alexander et al. 2017), so the 1600, 6400 and 25,600 km^2^ landscapes (40, 80, and 160 km per side, respectively) aim to approximate the range of outputs obtained from global land use models. One grid covering the terrestrial surface of the Earth, excluding Antarctica, was created per landscape extent using the same method as for creating landscapes across Colombia. Each of the six class-level landscape metrics was then calculated within each landscape in the five global-scale grids using the ‘landscapemetrics’ R package version 1.5.6 (Hesselbarth et al. 2019) in R version 4.0.0 (R Core Team 2020) for all years in the study period (1992 to 2020). We assessed whether the selected landscape metrics showed predictable scaling across landscape extents at global scale using the same method as for Colombia (Fig. 2). Note that a colour palette from the’rcartocolor’ R package version 2.1.1 (Nowosad 2018) was used for plotting the scaling relationships of landscape metrics.

### Analysis of landscape pattern change

The dataset of global landscape metrics from 1992 to 2020 was used to analyse landscape pattern change of six LULC classes through time. Given the large number of analyses carried out, we do not present all the results for all landscape extents in the main Results. However, as all six landscape metrics show consistent scaling relationships globally (Fig. 2) we expect our conclusions will be applicable across scales. We focused our analyses on the smallest landscape extent to increase the sample size for subregions (i.e., continents) and to better characterize relatively rare land cover classes, such as urban land cover (Fig. 2). Prior to analysing landscape pattern change during the study period, we first processed landscape metrics in the same way as for assessing the scaling relationships of landscape metrics. First, we removed HILDA+ classes representing water from the dataset. Next, missing values were replaced with zero for any landscape metrics where we considered a missing value to be equivalent to zero (Table S1).

Landscape metrics were summarised by calculating the mean and standard deviation across all landscapes of a given extent in each year for every LULC class. Calculating the mean and standard deviation allowed us to assess the average and variation in landscape patterns across the globe from 1992 to 2020, and whether the trends were consistent through time. Additionally, we assessed the spatiotemporal variation in landscape pattern change by calculating the net change within each landscape between 1992 and 2020 for every landscape metric and LULC class. Net change was plotted for each metric and LULC class for 100 km^2^ extent landscapes only to evaluate whether the magnitude and direction of change varied between landscapes and regions (Figs. S 29–S 34). We also calculated the average net change in landscapes for each continent to test for differences between continental- and global-scale landscape pattern change.

To disentangle the impacts of changes in the area and configuration of LULC classes, we focused on two particular indices: class area (CA), which measures the area (composition) of a LULC class in a landscape, and Landscape Shape Index (LSI). LSI is a good measure of changes in configuration independently of CA because it calculates edge length in relation to a hypothetical minimum edge length for a given area, and hence accounts for the area of a LULC class within the landscape. A high LSI value indicates a LULC class with high fragmentation per se (Fahrig 2003; configuration changes only), as the actual edge length is much longer than the hypothetical minimum if the LULC class was as aggregated as possible. Overall, LSI provides a better measure of configuration independently of CA than total edge (TE), the patch metric NP, or the core area metrics NDCA and TCA, because it a) can account for multiple configurational changes, including alterations in shape, edge amount, and number of patches (Lockhart and Koper 2018), and b) is only weakly correlated with CA at the landscape scale when habitat amount varies from 20 to 60% (Wang et al. 2014; Lockhart and Koper 2018). All six scale invariant landscape metrics that we identified here are associated with habitat area (Wang et al. 2014), hence none completely capture changes in landscape configuration independently of composition.

To identify landscapes where both CA and LSI were changing in the same direction for one LULC class, we first selected all landscapes of a given extent that contained that class in both 1992 and 2020. Next, the selected landscapes were classified into nine categories: both CA and LSI increasing (CA+LSI+); CA increasing and LSI decreasing (CA+LSI−); CA increasing and no change in LSI (CA+LSI=); CA decreasing and LSI increasing (CA−LSI+); both CA and LSI decreasing (CA−LSI−); CA decreasing and no change in LSI (CA−LSI=); no change in CA and increasing LSI (CA=LSI+); no change in CA and decreasing LSI (CA=LSI−), and no change in either CA or LSI (CA=LSI=). Maps of the nine categories of CA and LSI change were created to examine whether there was a tendency for regions and LULC classes to exhibit configurational changes over time as LULC area changed. The percentage of landscapes in each of the nine categories was calculated at global scale for each landscape extent and at global and continental scales for 100 km^2^ landscapes, to test whether the prevailing direction of change differed between LULC classes and across scales.

## Results

### Scaling relationships of landscape metrics

The mean values of the selected landscape metrics (CA, LSI, NDCA, NP, TCA and TE) exhibited predictable behaviour with increasing landscape extent at global scale in 1992 (Fig. 2). Five out of the six metrics appeared to exhibit a power-law relationship with landscape extent, whereas LSI had an approximately linear relationship with landscape extent. The relationship between each metric and landscape extent followed the same pattern across LULC classes, although the rate of increase differed between LULC classes. For example, forest CA increased much more rapidly with landscape extent compared to urban CA, likely because urban areas will not cover more than a small fraction of a 25,600 km^2^ landscape.

### Global trends in landscape patterns

**Fig. 3:**
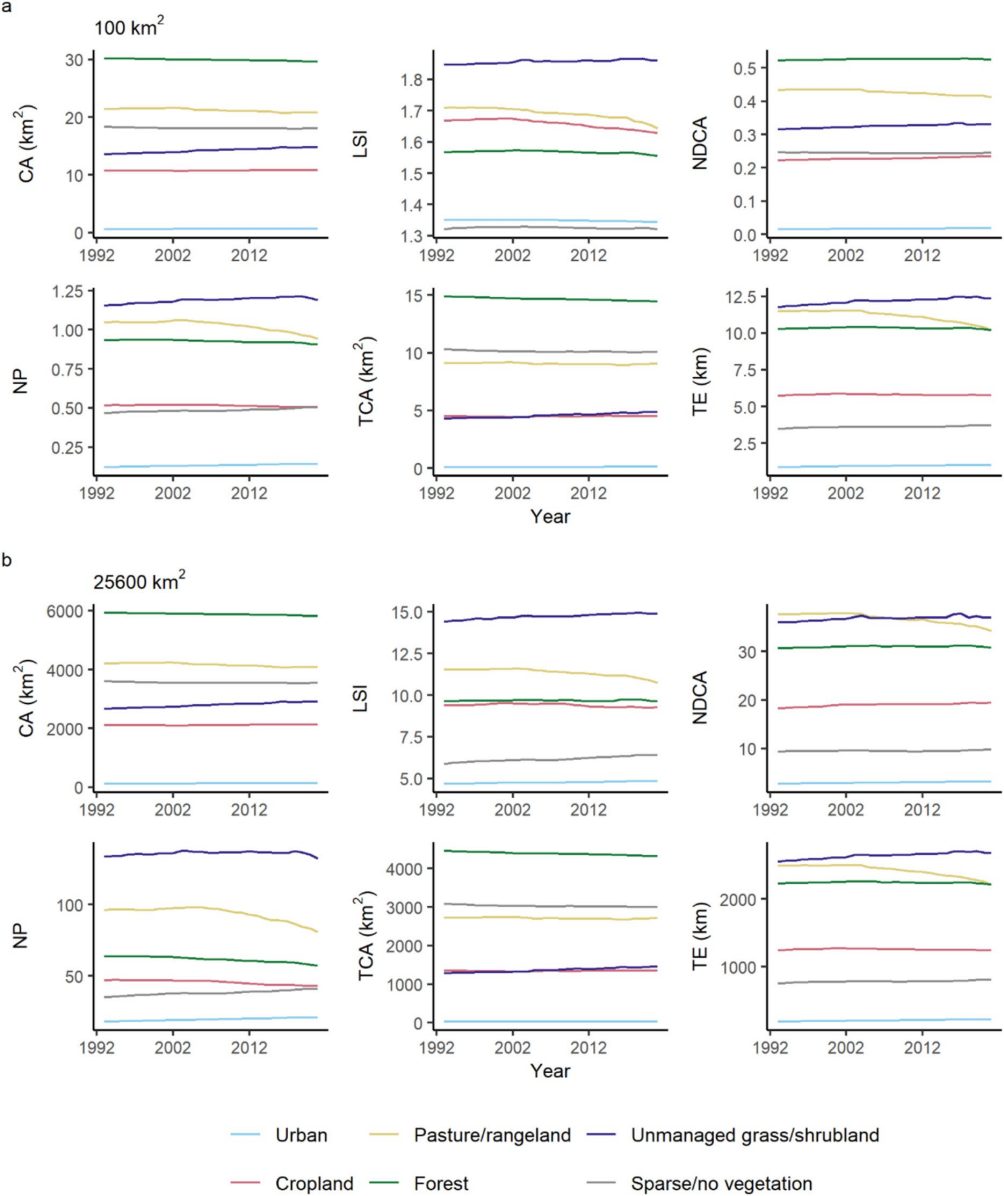
Average of global-scale landscape metrics in 100 km^2^ (**a**) and 25,600 km^2^ (**b**) extent landscapes from 1992 to 2020. Lines give the mean of one landscape metric for one LULC class across landscapes of that extent in every year from 1992 to 2020. See Fig. 2 for landscape metric definitions and units. Standard deviations were large (e.g., minimum 0.15 for NDCA and maximum 39.67 km^2^ for CA in 100 km^2^ landscapes) and are plotted separately in Figs. S 6 and S 10

The global mean values of landscape metrics for the urban and unmanaged grass/shrubland LULC classes tended to increase from 1992 to 2020 in landscapes of 100 and 25,600 km^2^ extent (Fig. 3). For example, the mean area of unmanaged grass/shrubland increased from 13.62 ± 25.45 km^2^ (mean ± standard deviation) in 1992 to 14.92 ± 26.58 km^2^ in 2020 in 100 km^2^ landscapes, with a corresponding increase in all other metrics. Similarly, there were increases in five out of six landscape metrics for urban LULC in 100 km^2^ landscapes, with only LSI exhibiting a small decrease from 1.35 ± 0.45 in 1992 to 1.34 ± 0.45 in 2020. Therefore, unmanaged grass/shrubland and urban land cover both increased on average in 100 km^2^ landscapes between 1992 and 2020, which coincided with increasing core area and number of patches, and in the case of unmanaged grass/shrubland an increase in fragmentation per se as measured by LSI.

By contrast, pasture/rangeland and forest LULC exhibited a decrease in area on average in most landscape metrics in 100 km^2^ landscapes across the study period. For instance, at global scale all metrics declined for pasture/rangeland from 1992 to 2020, except for TCA which showed no net change between the two years. For forest cover, there was a decrease in five out of six landscape metrics from 1992 to 2020 (CA, LSI, NP, TCA, and TE), and a very small expansion in NDCA (0.52 ± 0.77 in 1992 and 0.53 ± 0.77 in 2020). The magnitude of changes in landscape metrics were larger for pasture/rangeland than forest.

The direction of change in landscape metrics in 100 km^2^ landscapes from 1992 to 2020 was more variable for the cropland and sparse/no vegetation LULC classes, and the changes in each metric were smaller compared to the fluctuations in other LULC classes. Three out of six landscape metrics demonstrated an increase on average in 100 km^2^ landscapes for cropland from 1992 to 2020, with LSI, NP and TCA showing small reductions (LSI = 1.67 ± 0.58 in 1992 and LSI = 1.63 ± 0.53 in 2020; NP = 0.52 ± 1.12 in 1992 and NP = 0.51 ± 1.06 in 2020; TCA = 4.54 ± 13.95 km^2^ in 1992 and 4.52 ± 13.64 km^2^ in 2020). Comparatively, there were small decreases in CA, NDCA, and TCA for sparse/no vegetation, increases in NP and TE, and no change in LSI.

The behaviour of landscape metrics over time between 1992 and 2020 was generally similar in 100 km^2^ and 25,600 km^2^ landscapes (Fig. 3), which was expected as the landscape metrics were selected to show predictable scaling behaviour across landscape extents. However, there were differences in the comparative magnitude of some metrics within 100 km^2^ versus 25,600 km^2^ landscapes. For instance, unmanaged grass/shrubland NP was 10.2% higher than the pasture/rangeland NP in 100 km^2^ landscapes in 1992, and 38.6% higher in landscapes of 25,600 km^2^ extent. Cropland LSI was 6.5% higher than forest LSI in 100 km^2^ extent landscapes in 1992, whereas in 25,600 km^2^ landscapes cropland LSI was 2.6% less than forest LSI. These differences when comparing landscape metrics between LULC classes across scales could be due to variability in the shape of the relationship between landscape metrics and landscape extent for different LULC classes (Fig. 2).

**Fig. 4:**
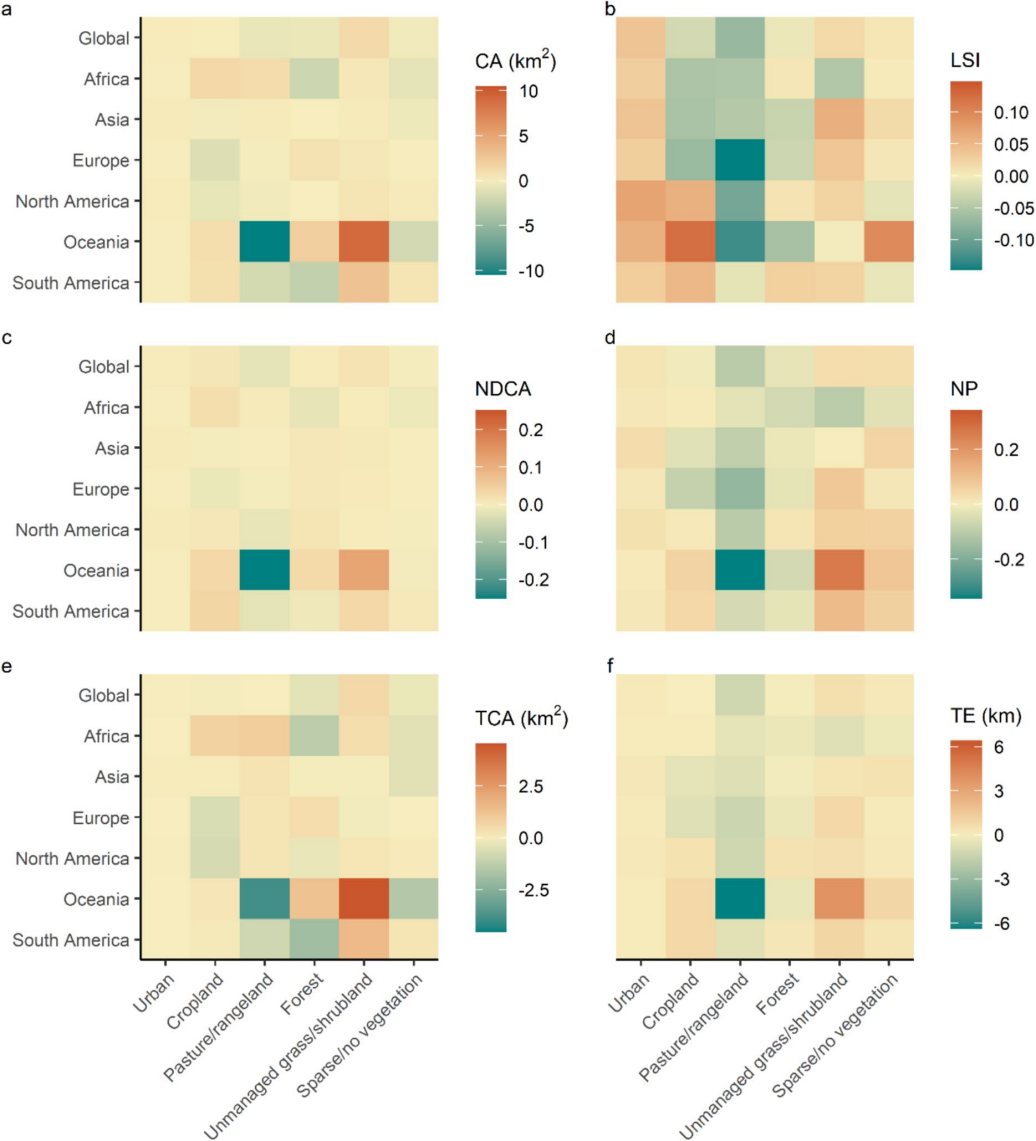
Average net change in landscape metrics across continents from 1992 to 2020. Average net change is shown for six LULC classes for landscapes of 100 km^2^ extent. See Fig. 2 for descriptions of landscape metrics and units

There was considerable variation in the direction and magnitude of landscape pattern changes across continents (Fig. 4). For example, while pasture/rangeland CA declined globally the largest average net decrease was for Oceania (mean and standard deviation of −10.47 ± 30.74 km^2^) whereas there was net expansion on average in Africa (1.17 ± 11.12 km^2^) and Asia (0.22 ± 10.46 km^2^). LSI for pasture/rangeland declined on average across all continents, with the largest decrease in Europe (−0.15 ± 0.46) and smallest in South America on average (−0.01 ± 0.37). Similarly, unmanaged grass/shrubland CA expanded on average in all continents while NP and TE increased for all continents except Africa, although the decreases in these two metrics across Africa were small (−0.10 ± 0.96 and −0.76 ± 9.16 km for NP and TE, respectively). Net changes in the patterns of forest and cropland were particularly variable among continents; for instance, forest CA increased in Oceania, Europe and Asia but declined in South America, Africa and North America. In general, the trends in average landscape metrics were consistent through time between 1992 and 2020 in landscapes of 100 km^2^ extent (Figs. S 11–S 16), although there were exceptions to this pattern. For example, average NP of pasture/rangeland increased in North America from 1998 to 2005 but showed a consistent decline from 2005 onwards. Overall, there was considerable variability in landscape pattern change from 1992 to 2020 between continents, and the direction of change at continental-scale was not always the same as at a global scale.

### Directional change in LULC area and configuration

**Fig. 5:**
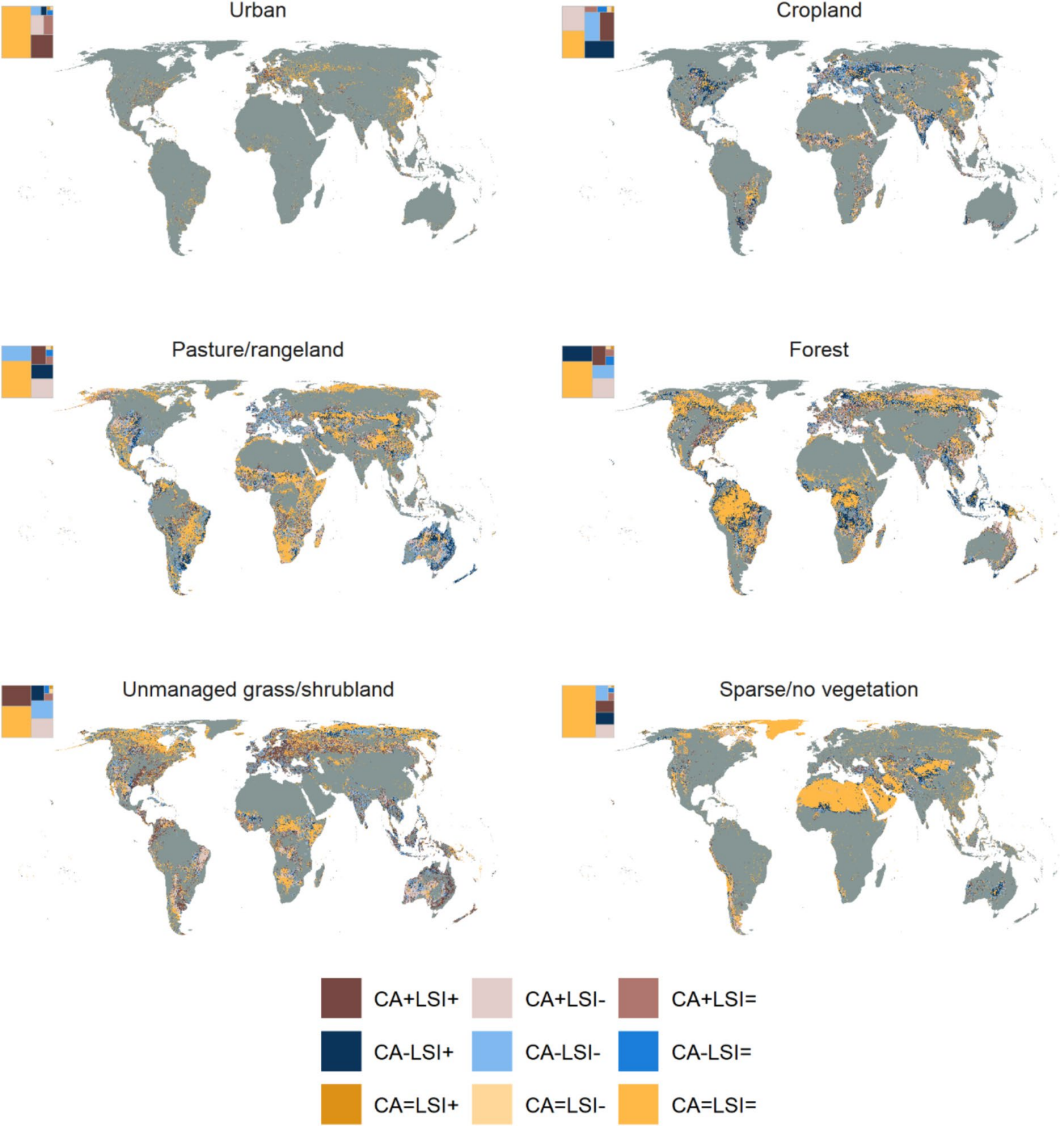
Direction of net change in area and configuration in 100 km^2^ landscapes from 1992 to 2020. Direction of net change is shown for landscapes that contained the land use and land cover (LULC) class of interest in both 1992 and 2020. CA = class area, LSI = Landscape Shape Index. The square inset in each panel shows the relative proportion of landscapes assigned to each of the nine categories of CA and LSI change for that LULC class. Grey shading indicates the absence of a LULC class in a landscape in both 1992 and 2020. Note that LSI+ indicates increased fragmentation per se of a LULC class and LSI− represents decreased fragmentation per se

**Fig. 6:**
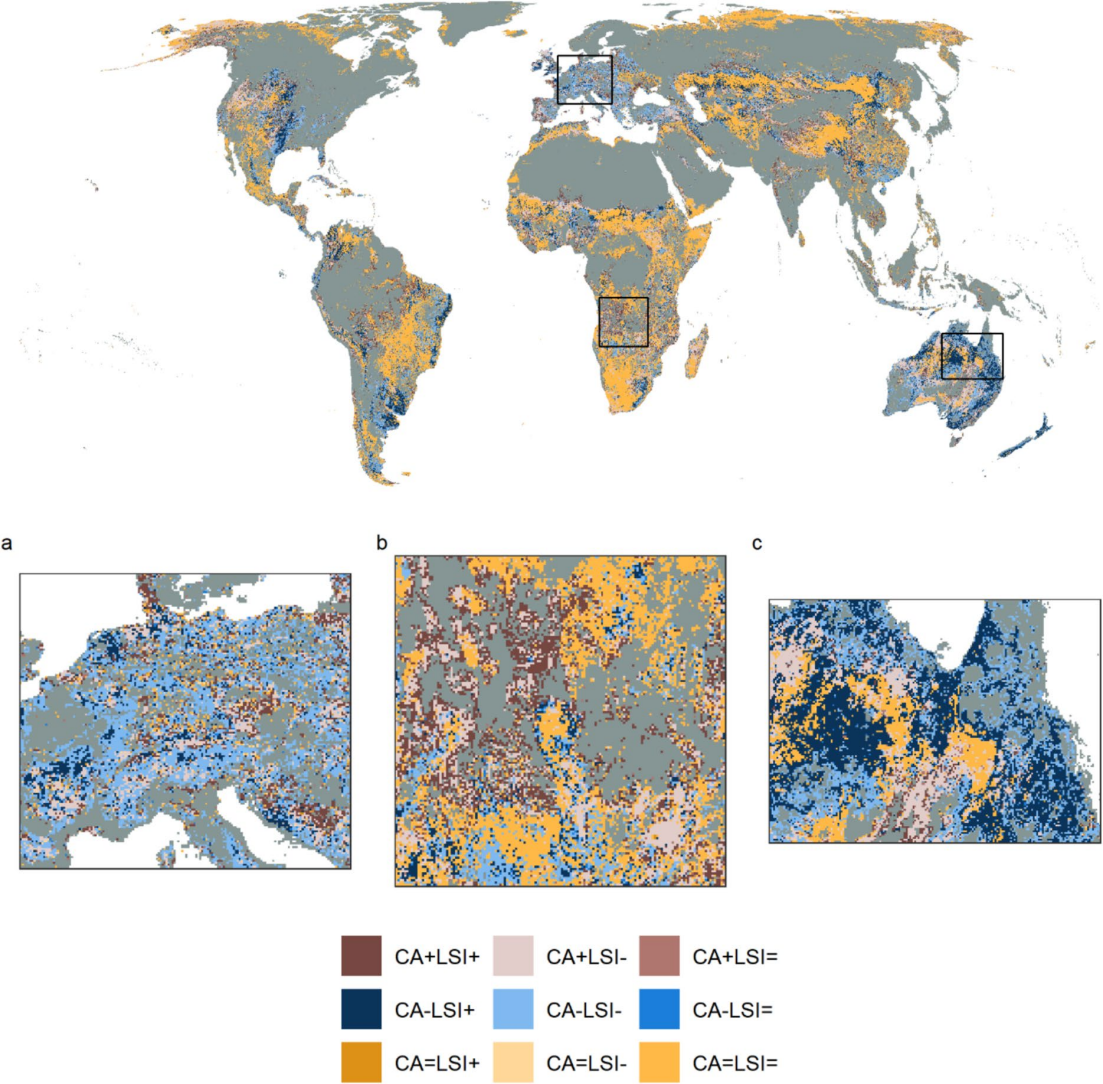
Direction of net change in area and configuration for pasture/rangeland from 1992 to 2020. Directional change is shown for example regions in **a** central Europe, **b** southern Africa, **c** northeastern Australia. CA = class area, LSI = Landscape Shape Index. See Fig. 5 for further information on CA and LSI categories

In addition to examining the global trends in landscape patterns, we investigated how often increases in the area of a LULC class were associated with configurational changes across scales. To assess changes in area and configuration, landscapes of 100 km^2^ extent that contained a LULC class in both 1992 and 2020 were classified as to whether they showed an increase (+), decrease (−), or no change (=) in CA and LSI between 1992 and 2020. The most common pattern for all LULC classes was no net change in CA or LSI between 1992 and 2020, so the majority of landscapes were categorised as CA=LSI= (Figs. 5 and 6). However, the second-most common category of CA and LSI change varied between LULC classes. For instance, the second-most prevalent category for the urban and unmanaged grass/shrubland LULC classes was CA+LSI+ (19.97 and 23.22% for urban and unmanaged grass/shrubland, respectively), whereas the second-most frequent category was CA−LSI− (16.91%) for pasture/rangeland and CA−LSI+ for forest (17.70%). The predominant patterns of LULC change were therefore divergent across LULC classes over the past three decades, although in multiple cases there was little difference in the frequency of each change category at global scale; for example, for cropland the frequencies of the categories where both CA and LSI changed were 20.28% for CA+LSI−, 19.15% for CA−LSI+, 16.27% for CA−LSI−, and 15.20% for CA+LSI+.

There was more variation in the prevalence of each category at continental scale, however (Fig. S 23). For instance, more than 50% of landscapes exhibited a decrease in cropland CA in Europe across the study period (CA−LSI+  = 26.36%, CA−LSI− = 26.29%, and CA−LSI= = 2.55%), whereas in Africa the majority of landscapes containing cropland showed an increase in CA from 1992 to 2020 (CA+LSI+  = 22.34%, CA+LSI− = 30.60% and CA+LSI=  = 3.56%). Hence, cropland was more likely to increase in landscapes in Africa and decrease in landscapes in Europe, while changes in configuration were split relatively evenly between increasing and declining fragmentation per se. There were also differences in the most common categories of CA and LSI change across landscape extents, with the CA=LSI= category becoming less frequent as landscape extent was increased at global scale (Fig. S24).

**Fig. 7:**
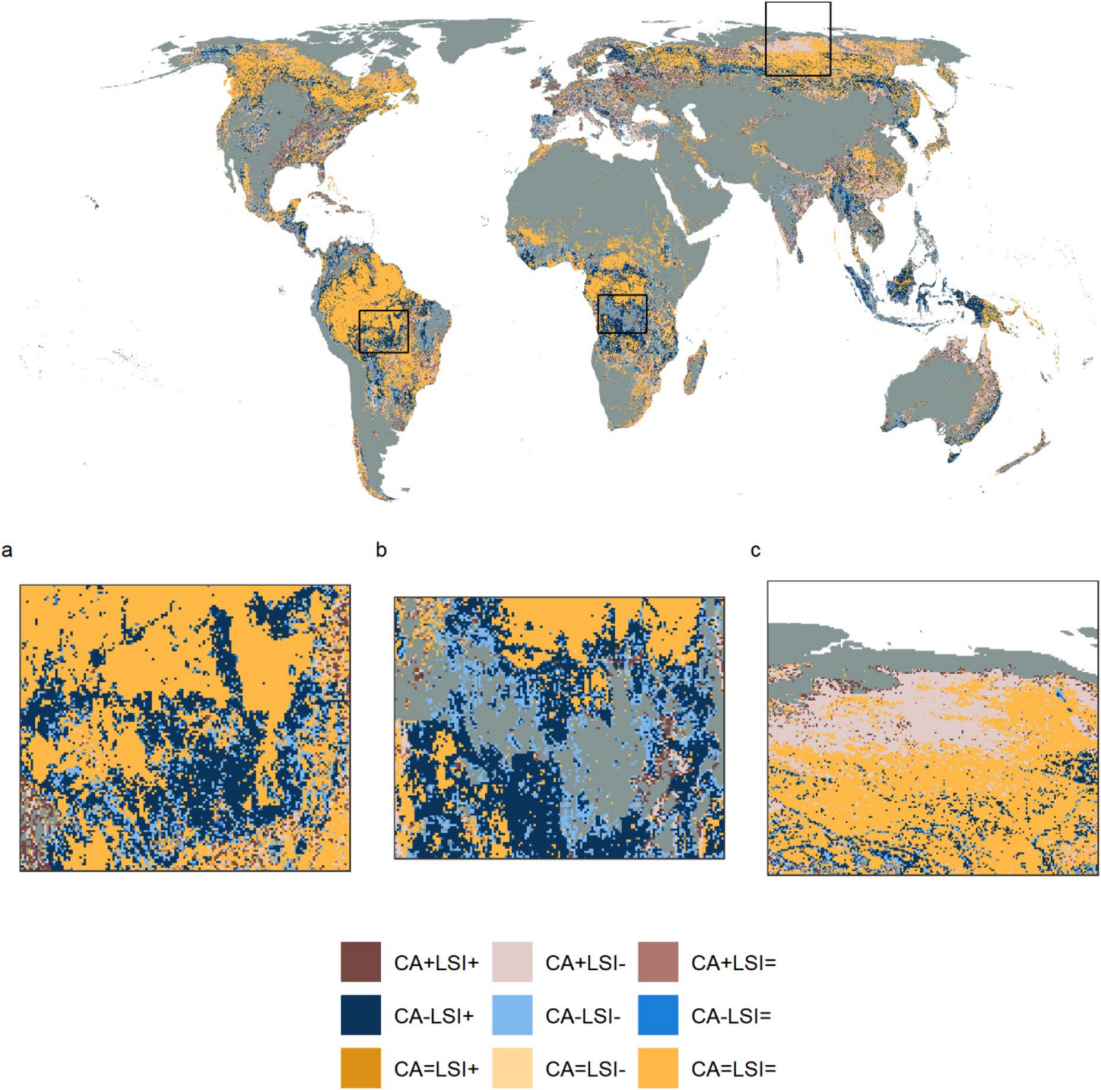
Direction of net change in area and configuration of forest cover from 1992 to 2020. Directional change is shown for example regions in **a** the southern Amazon, **b** southern Africa, **c** northern Russia. CA = class area, LSI = Landscape Shape Index. See Fig. 5 for further information on CA and LSI categories

There was considerable spatial variation in the directional net change in CA and LSI within 100 km^2^ landscapes that contained a specific LULC class in both 1992 and 2020 (examples shown for pasture/rangeland and forest in Figs. 6 and 7, respectively). For example, loss of pasture/rangeland in Europe was more commonly classified as CA−LSI− (22.78%) rather than CA−LSI+ (5.87%; Fig. 6a and Fig. S 23), indicating that remaining pasture/rangeland area was less fragmented in 2020 compared to 1992. Given that CA, NP, and TE of pasture/rangeland declined and TCA increased slightly on average across Europe during the study period (Fig. 4, Figs. S 11 and S 14–S 16), the apparent decrease in pasture/rangeland fragmentation may be due to the loss of small farms, which would leave large-scale farms with less complex boundaries, and hence lower edge relative to their area (as measured by LSI) and lower fragmentation per se. There were extensive changes in area and configuration of pasture/rangeland and unmanaged grass/shrubland across Australia, with a trend towards increased unmanaged grass/shrubland and decreased pasture/rangeland cover (Fig. S 11). Directional changes in area and configuration of pasture/rangeland were often spatially aggregated within Australia, with large areas of the northeast of the country classified as CA−LSI+, for example (Fig. 6c). Pasture/rangeland area increased across Africa during the study period (Fig. 4 and Fig. S 11), with the increases more commonly classified as CA+LSI− (17.75%) than CA+LSI+ (9.99%; Fig. S 23). However, as for Europe and Australia there was considerable local heterogeneity in the direction of pasture/rangeland changes (Fig. 6b), with the CA+LSI− and CA+LSI+ categories often clustered together.

Forest cover also showed spatial variation in the direction of net change in CA and LSI within 100 km^2^ landscapes from 1992 to 2020, both across and within continents (Fig. 7). For instance, CA−LSI+ was the second most common category for forest change in South America and Africa (18.76 and 21.45%, respectively; Fig. S 23). Regions of the southern Amazon and Congo Basin both demonstrated a high number of landscapes classified as CA−LSI+ , especially adjacent to stable (CA=LSI=) forest areas, between 1992 and 2020 (Fig. 7). However, other categories including CA+LSI+, CA+LSI−, and CA−LSI−, were also represented within the same regions. Indeed, landscapes with net decrease in area and fragmentation (CA−LSI−) were often adjacent to or clustered near those in the CA−LSI+ category. CA−LSI+ may represent large forest areas being converted into numerous smaller patches, whereas CA−LSI− may indicate landscapes that were already partially deforested in 1992 and lost individual small forest patches over the study period, leaving lower forest cover and lower fragmentation per se (independent of area). In comparison, CA+LSI− was the second most common category for area and configuration change across Europe (20.84%; Fig. S 23) and is exemplified by the increase in forest area and corresponding decrease in fragmentation across northern Russia (Fig. 7c). Overall, there was spatial variation in the directional net change in CA and LSI both across and within continents, with neighbouring landscapes often showing the same directional change in area and configuration.

## Discussion

This study calculated landscape metrics at global scale from 1992 to 2020 for six LULC classes and five landscape extents, and assessed changes in the area and configuration of LULC over the past three decades. We found that the unmanaged grass/shrubland and urban LULC classes expanded but showed relatively limited configurational changes independent of changes in area. Comparatively, forest and pasture/rangeland declined in area and became less fragmented, with corresponding decreases in number of patches (NP), total edge length (TE), and Landscape Shape Index (LSI). The magnitude and direction of changes in landscape metrics varied spatially between and within continents, which has implications for predicting landscape pattern change and understanding its environmental impacts.

At global scale, urban LULC showed increases in area, number of patches, edge length, and core area, indicating mostly changes in area rather than configuration. Urban LULC is known to have expanded globally since 1970 (van Vliet 2019; Güneralp et al. 2020; Liu et al. 2020), hence our findings agree with previous studies. Urban LULC change can drive both increased and decreased fragmentation of natural land cover classes of landscapes (Irwin and Bockstael 2007; Schneider and Woodcock 2008), likely due to factors such as the price of land and geographic constraints on expansion (Angel et al. 2012). However, our analyses suggest that at the scales we measure, the main change in urban LULC at global scale is its increase; this has implications for food security and the environment depending on whether urban areas expand into cropland or natural LULC classes.

Changes in the landscape patterns of forest LULC were spatially variable, with forests increasing in area and becoming less fragmented in Europe, Asia, and Oceania but declining and fragmenting in Africa, South America and North America. Although we summarised landscape metrics across continents rather than biomes or climatic zones (as in Ma et al. 2023), it appeared that tropical regions such as the southern Amazon and Congo Basin experienced high levels of forest loss and increased fragmentation between 1992 and 2020 (Fig. 7). However, other tropical areas were more likely to exhibit increased forest cover and decreased fragmentation (e.g., southeastern China and northwestern Australia, Fig. 7). The loss and fragmentation of tropical forest has implications for biodiversity loss (Alroy 2017; Giam 2017), carbon storage (Chaplin-Kramer et al. 2015; Shapiro et al. 2016) and carbon emissions (Brinck et al. 2017; Broggio et al. 2024).

Ma et al. (2023) found that approximately 75% of global forest landscapes with exactly 25 km^2^ extent became less fragmented between 2000 and 2020, whereas only 26.8% of forest landscapes with 100 km^2^ extent showed decreased fragmentation in terms of LSI between 1992 and 2020 here. There were multiple differences in methodology between the two studies that may explain this discrepancy. For example, net change in forest LSI was calculated from 1992 to 2020 in this study, whereas net change in forest fragmentation was assessed between 2000 and 2020 in Ma et al. (2023). Increases in forest fragmentation from 1992 to 2000 may have been counteracted by decreases between 2000 and 2020, leading to a lower proportion of landscapes identified as having decreased fragmentation in this study. Similarly, input LULC maps of 1 km resolution (this study) versus 30 m resolution (Ma et al. 2023) were used to calculate forest landscape metrics. Coarse resolution maps are more homogeneous than fine resolution ones (Wiens 1989), so Ma et al. (2023) may have detected fine-scale changes in forest patterns that were apparent at 30 m but not 1 km resolution. Furthermore, different metrics were used to quantify fragmentation (LSI versus a normalised index of edge density, patch density, and mean patch area; Ma et al. 2023), which could have influenced the classification of landscapes into increased versus decreased fragmentation in the two studies. Indeed, Wang et al. (2014) show these indices vary in complex ways in how well they capture fragmentation independently of habitat amount (fragmentation per se). A similar definition of forest was used in both studies, with forest defined as trees with height of ≥ 5 m in Ma et al. (2023) and as trees with height of > 5 m and cover ≥ 10% in the HILDA+ dataset (Winkler et al. 2021, 2025), but even these small differences may have contributed to the differing conclusions between the studies.

The pasture/rangeland and unmanaged grass/shrubland LULC classes showed the largest changes in area and configuration at global scale from 1992 to 2020. Pasture/rangeland is acknowledged to have declined globally since about the year 2000 (Blaustein-Rejto et al. 2019; Winkler et al. 2021), but the outcomes of pasture/rangeland declines will depend on what they are replaced by. For example, replacement of pasture/rangeland with unmanaged grass/shrublands may have positive implications for the environment such as through providing additional land for biodiversity (Poore 2016) and carbon sequestration (Silver et al. 2000), whereas replacing pasture/rangeland with urban LULC would likely not have environmental benefits. However, the replacement of pasture/rangeland with unmanaged grass/shrubland does not necessarily lead to positive outcomes. For instance, plant diversity does not always recover after land abandonment (Cava et al. 2018; Isbell et al. 2019) and land abandonment in cultural landscapes with low intensity land use can lead to biodiversity loss (Daskalova and Kamp 2023). Similarly, the occurrence of new unmanaged grass/shrubland patches at a distance from existing ones may increase the time taken for species to colonise the new patches, leading to much longer time lags in biodiversity recovery compared to creating new patches near to current ones (Synes et al. 2020). Overall, the global decrease in pasture/rangelands and increase in unmanaged grass/shrublands suggests that there are emerging opportunities for ecosystem recovery and restoration, although management actions may be needed to ensure that environmental outcomes are positive.

Again, the changes in unmanaged grass/shrubland we observed appear to have mainly been in area, rather than configuration; however, difficulties in classifying LULC as pasture or rangeland versus unmanaged land make this uncertain (Phelps and Kaplan 2017). This is also illustrated by the greater uncertainty associated with the grassland-related classes unmanaged grass/shrubland and pasture/rangeland in HILDA+ (Winkler et al. 2021). Moreover, the changes in the patterns of pasture/rangeland and unmanaged grass/shrubland LULC in our study are largely driven by shifts in LULC across Oceania, and particularly Australia (Figs. 4 and 6). Australia has extensive areas of both managed and unmanaged grasslands, which drives high uncertainty in these regions in HILDA+ because the underlying input datasets use different classifications for pasture and grasslands and it is difficult to delineate unmanaged versus low intensity grasslands within heterogeneous rangeland landscapes (Winkler et al. 2021). Additionally, the uncertainty in HILDA+ is time-dependent, with higher uncertainty in earlier years (such as 1992) due to limited availability of high-resolution input data, whereas improved spatial detail in more recent years (such as 2020) allows for better distinction between managed and unmanaged grasslands. This temporal variation in uncertainty can affect not only configuration metrics, but also area estimates (e.g., CA and core area metrics), as class confusion is more likely in years with higher uncertainty. Therefore, it is unclear whether the spatial patterns of pasture/rangeland and unmanaged grass/shrubland across Australia, and the rest of the globe, are real or an artifact of the HILDA+ data. Some of the observed changes in area and configuration of managed versus unmanaged grasslands may therefore partially reflect improvements in input data quality and classification accuracy over time, rather than solely real-world landscape changes.

While efforts have been made to harmonize LULC data and classification standards at the global scale, including the Land-Use Harmonization 2 (LUH2; Hurtt et al. 2020) dataset and the FAO/UNEP Land Cover Classification System (LCCS; Di Gregorio and Jansen 2005), challenges remain in achieving consistent, high-resolution mapping across diverse landscapes and research objectives. LUH2, for example, provides globally consistent land use data with broad class definitions at a coarse spatial resolution (~ 28 km), making it well-suited for Earth system modelling but less applicable for fine-scale landscape analyses. In contrast, HILDA+ (used in this study) offers higher spatial resolution (1 km) and more detailed thematic classes, but still faces limitations in distinguishing between land use intensities, particularly for pasture/rangeland and unmanaged grass/shrubland. The LCCS provides a flexible framework for standardisation, yet its implementation is often constrained by input data availability and the need for expert interpretation. Our findings therefore highlight the ongoing need for refinement in harmonisation practices, especially for LULC classes where classification uncertainty remains high.

Further research is needed to build on these harmonization efforts by advancing our capacity to identify land use intensity and distinguish between managed pastures and unmanaged grass/shrublands in LULC datasets, as well as by refining and consolidating class definitions in line with existing frameworks such as LCCS. Rather than proposing a wholly new unified classification system, we suggest that future work should focus on improving the interoperability, thematic detail, and uncertainty quantification of LULC datasets, particularly for classes that are currently subject to high classification uncertainty. These steps will help clarify whether observed changes in landscape patterns reflect real-world processes or are artifacts of evolving data quality and classification approaches.

Changes in landscape patterns were highly spatially variable for all LULC classes, both across and within continents, suggesting the drivers of landscape pattern change act across a range of scales. For example, pasture/rangeland area increased on average in Africa and Asia over the study period, but decreased on average in all other continents (Fig. 4), while the fragmentation of pasture/rangeland declined across all continents. The decline in pasture/rangeland area and fragmentation observed in Europe may be related to the loss of small farms across the continent between 2005 and 2020, leading to fewer, larger remaining farms (Eurostat 2022) and hence less spatially complex landscapes. Conversely, factors such as population growth might have driven the expansion of pasture/rangeland across Africa (Assede et al. 2023). At a regional scale, the net expansion of pasture/rangeland, coupled with both increased and decreased fragmentation, in southern Africa (Fig. 6b) may be associated with shifting cultivation, where forests are regularly cleared for agriculture and then fallowed (Schneibel et al. 2017). All categories of directional area (CA) and fragmentation (LSI) change were represented across the majority of continents and LULC classes (Fig. 5 and Fig. S 23), suggesting that these trajectories of change are not unique to specific classes or regions. The spatial heterogeneity in CA and LSI categories, combined with clustering of landscapes belonging to the same category (Figs. 6 and 7), indicates that landscape pattern changes are likely shaped by local- as well as continental-scale drivers. Further research could relate landscape pattern changes to known drivers of LULC change, in order to identify key drivers of landscape pattern dynamics and better represent these processes in land use models. It may also be possible to use historic landscape pattern change in a landscape or region to predict future changes, although it is unclear how well past change can predict the future (as evidenced for cropland expansion in Eigenbrod et al. 2020).

The global dataset of landscape metrics generated here could be used for further research into the dynamics of landscape pattern change. We only assessed temporal changes in landscape patterns at global- and continental-scales. Hence, future work could expand this temporal analysis by identifying regions or landscapes that showed varied temporal dynamics from 1992 to 2020, such as those where the temporal trend of fragmentation changed direction. A method such as RemotePARTS (Ives et al. 2021; Morrow and Ives 2025) could be applied to test for statistical differences in landscape pattern changes across LULC classes and continents or other spatial units. Changes in landscape patterns could also be assessed across different spatial units, such as climatic zones or biomes, to highlight areas that have experienced the largest changes in landscape patterns over the past few decades. Future work could examine the patterns of LULC transitions in addition to changes in the patterns of individual LULC classes, to understand whether fragmenting LULC classes are being converted to natural or anthropogenic LULC classes and how this in turn impacts the environment. Disentangling the effects of habitat amount and configuration using more landscape metrics at a single landscape extent could be another focus of future research, as unfortunately none of the landscape indices showing predictable behaviour across landscape extents are also entirely uncorrelated with area, though LSI is known to be a reasonable measure of fragmentation per se between 20 and 60% class area (Wang et al. 2014).

In conclusion, global-scale landscape pattern change demonstrated considerable heterogeneity between 1992 and 2020. To our knowledge, this is the first study to quantify landscape metrics globally for multiple scales (in terms of landscape extents) and LULC classes. Although trends in landscape metrics were generally consistent across landscape extents, the differing relationships between landscape metrics and landscape extent across LULC classes highlights the importance of considering multiple spatial scales and LULC classes when assessing landscape pattern change. The spatial heterogeneity in landscape patterns detected here suggests that landscape pattern change should be accounted for when quantifying and predicting LULC change, especially as landscape patterns are key for a range of ecological processes such as species movement and carbon storage. We anticipate that the global dataset of landscape metrics calculated in this study will have further applications in establishing links between landscape metrics and environmental processes such as biodiversity loss, carbon emissions, and habitat degradation. The inclusion of scale invariant landscape metrics for multiple landscape extents will allow for multiscale studies of the relationships between landscape patterns and environmental phenomena, thereby furthering our understanding of the impacts of the spatial pattern of LULC on the environment.

## Supplementary Information

Below is the link to the electronic supplementary material.

Supplementary file1 (DOCX 12748 KB)

## Data Availability

The HILDA+ version 2b dataset will be made publicly available on Zenodo at a later date (10.5281/zenodo.15017066). The global dataset of landscape patterns generated in this study is publicly available on Zenodo (10.5281/zenodo.15120267), as is the code used to generate and analyse the dataset (10.5281/zenodo.15124527).
