# Supplementary material for: Global assessment of landscape pattern changes from 1992 to 2020
Source: Landsc Ecol. 2025 Oct 16;40(11):196. doi: 10.1007/s10980-025-02210-0 (PMC12532760; doi:10.1007/s10980-025-02210-0)
Supplement: Supplementary file 1 — Supplementary file1 (DOCX 12748 KB) [file 10980_2025_2210_MOESM1_ESM.docx]

Global assessment of landscape pattern changes from 1992 to 2020

# Landscape Ecology

Tamsin L. Woodman^1,2^*, Peter Alexander^2,3^, David F.R.P. Burslem^1^, Justin M.J. Travis^1^, Karina Winkler^4^, Felix Eigenbrod^5^

^1^University of Aberdeen, School of Biological Sciences, Zoology Building, Tillydrone Avenue, Aberdeen, AB24 2TZ

^2^University of Edinburgh, School of GeoSciences, University of Edinburgh, Drummond Street, Edinburgh, EH8 9XP, UK

^3^Global Academy of Agriculture and Food Systems, University of Edinburgh, Edinburgh, EH25 9RG, UK

^4^Land Use Change and Climate, IMKIFU, Karlsruhe Institute of Technology (KIT), Campus Alpin, Garmisch-Partenkirchen, Germany

^5^School of Geography and Environmental Science, University of Southampton, Highfield Campus, Southampton, SO17 1BJ, UK

* Corresponding author: tamsin.woodman@ed.ac.uk

| **Landscape metric** | **Abbreviation** | **Units** | **Description** | **Treatment of missing values** |
| --- | --- | --- | --- | --- |
| Aggregation Index | AI | Percent | Number of edges shared by the same LULC class divided by the hypothetical maximum possible number of edges that could be shared by that class. | NA |
| Coefficient of variation of patch area | AREA_CV | Square kilometres | Coefficient of variation of all patch areas that belong to a given LULC class. | NA |
| Mean of patch area | AREA_MN | Square kilometres | Mean of all patch areas that belong to a given LULC class. | NA |
| Standard deviation of patch area | AREA_SD | Square kilometres | Standard deviation of all patch areas that belong to a given LULC class. | NA |
| Class area | CA | Square kilometres | Total area of a LULC class in a landscape. | Zero |
| Coefficient of variation of core area index | CAI_CV | Percent | Coefficient of variation of the core area index for all patches that are classified as a given LULC class. Core area index is core area as a percentage of patch area. | NA |
| Mean of core area index | CAI_MN | Percent | Mean of core area index for all patches that are classified as a given LULC class. Core area index is core area as a percentage of patch area. | NA |
| Standard deviation of core area index | CAI_SD | Percent | Standard deviation of core area index for all patches that are classified as a given LULC class. Core area index is core area as a percentage of patch area. | NA |
| Coefficient of variation of related circumscribing circle | CIRCLE_CV | None | Coefficient of variation of the related circumscribing circle for all patches that are classified as a given LULC class. The related circumscribing circle is a ratio between patch area and the smallest circumscribing circle of a patch. | NA |
| Mean of related circumscribing circle | CIRCLE_MN | None | Mean of the related circumscribing circle for all patches that are classified as a given LULC class. The related circumscribing circle is a ratio between patch area and the smallest circumscribing circle of a patch. | NA |
| Standard deviation of related circumscribing circle | CIRCLE_SD | None | Standard deviation of the related circumscribing circle for all patches that are classified as a given LULC class. The related circumscribing circle is a ratio between patch area and the smallest circumscribing circle of the patch. | NA |
| Clumpiness index | CLUMPY | None | The proportional deviation of the number of edges shared by the same LULC class from the number of edges expected to be shared by that class under a random spatial distribution. | NA |
| Patch Cohesion Index | COHESION | Percent | Describes the connectedness of patches which belong to a given LULC class. | NA |
| Coefficient of variation of Contiguity index | CONTIG_CV | None | Coefficient of variation of the Contiguity index for all patches of a given LULC class. Contiguity index describes the spatial connectedness of the cells which make up a patch based on a focal filter approach. | NA |
| Mean of Contiguity index | CONTIG_MN | None | Mean of the Contiguity index for all patches of a given LULC class. Contiguity index describes the spatial connectedness of the cells which make up a patch based on a focal filter approach. | NA |
| Standard deviation of Contiguity index | CONTIG_SD | None | Standard deviation of the Contiguity index for all patches of a given LULC class. Contiguity index describes the spatial connectedness of the cells which make up a patch based on a focal filter approach. | NA |
| Coefficient of variation of core area | CORE_CV | Square kilometres | Coefficient of variation of the core area of all patches classified as a given LULC class. Core area is the area of grid cells within a patch that are not adjacent to a cell of a different LULC class. | NA |
| Mean of core area | CORE_MN | Square kilometres | Mean of the core area of all patches classified as a given LULC class. Core area is the area of grid cells within a patch that are not adjacent to a cell of a different LULC class. | NA |
| Standard deviation of core area | CORE_SD | Square kilometres | Standard deviation of the core area of all patches classified as a given LULC class. Core area is the area of grid cells within a patch that are not adjacent to a cell of a different LULC class. | NA |
| Core area percentage of landscape | CPLAND | Percent | The core area of a given LULC class as a percentage of the total area of the landscape. Core area is made up of grid cells within patches that are not adjacent to a cell of a different LULC class. | Zero |
| Disjunct core area density | DCAD | Number per square kilometre | The number of disjunct core area patches per square kilometre and in relation to the total area of the landscape. A disjunct core area patch has no neighbouring cells of another LULC class. | Zero |
| Coefficient of variation of number of disjunct core areas | DCORE_CV | None | Coefficient of variation of the number of disjunct core area patches for a given LULC class. A disjunct core area patch has no neighbouring cells of another LULC class. | NA |
| Mean of number of disjunct core areas | DCORE_MN | None | Mean of the number of disjunct core area patches for a given LULC class. A disjunct core area patch has no neighbouring cells of another LULC class. | NA |
| Standard deviation of number of disjunct core areas | DCORE_SD | None | Standard deviation of the number of disjunct core area patches for a given LULC class. A disjunct core area patch has no neighbouring cells of another LULC class. | NA |
| Landscape division index | DIVISION | Proportion | Defines the probability that two grid cells selected at random are not in the same patch of a specific LULC class. | NA |
| Edge density | ED | Kilometres per square kilometre | The sum of all edges of a given LULC class in a landscape as a proportion of the total landscape area. | Zero |
| Coefficient of variation of Euclidean nearest-neighbour distance | ENN_CV | Kilometres | Coefficient of variation of the distance to the nearest neighbouring patch of the same LULC class for all patches of a given LULC class. | NA |
| Mean of Euclidean nearest-neighbour distance | ENN_MN | Kilometres | Mean of the distance to the nearest neighbouring patch of the same LULC class for all patches of a given LULC class. | NA |
| Standard deviation of Euclidean nearest-neighbour distance | ENN_SD | Kilometres | Standard deviation of the distance to the nearest neighbouring patch of the same LULC class for all patches of a given LULC class. | NA |
| Coefficient of variation of fractal dimension index | FRAC_CV | None | Coefficient of variation of the fractal dimension index of all patches classified as a given LULC class. Fractal dimension index characterises the complexity of a patch according to the patch perimeter and area. | NA |
| Mean of fractal dimension index | FRAC_MN | None | Mean of the fractal dimension index of all patches classified as a given LULC class. Fractal dimension index characterises the complexity of a patch according to the patch perimeter and area. | NA |
| Standard deviation of fractal dimension index | FRAC_SD | None | Standard deviation of the fractal dimension index of all patches classified as a given LULC class. Fractal dimension index characterises the complexity of a patch according to the patch perimeter and area. | NA |
| Coefficient of variation of the radius of gyration | GYRATE_CV | Kilometres | Coefficient of variation of the radius of gyration across all patches classified as a given LULC class. The radius of gyration quantifies the distance between each cell in a patch and the patch centroid. | NA |
| Mean of the radius of gyration | GYRATE_MN | Kilometres | Mean of the radius of gyration across all patches classified as a given LULC class. The radius of gyration quantifies the distance between each cell in a patch and the patch centroid. | NA |
| Standard deviation of the radius of gyration | GYRATE_SD | Kilometres | Standard deviation of the radius of gyration across all patches classified as a given LULC class. The radius of gyration quantifies the distance between each cell in a patch and the patch centroid. | NA |
| Interspersion and Juxtaposition index | IJI | Percent | Characterises the intermixing of LULC classes within a landscape. | NA |
| Largest Patch Index | LPI | Percent | Describes the percentage of the landscape covered by the largest patch of a given LULC class. | Zero |
| Landscape Shape Index | LSI | None | Ratio between the actual edge length of a LULC class in a landscape and its theoretical minimum edge length if it were as aggregated as possible. | NA |
| Effective Mesh Size | MESH | Square kilometres | The sum of squared patch areas for a given LULC class in relation to the total area of the landscape, which gives a measure of patch structure. | NA |
| Number of disjunct core area patches | NDCA | None | Number of core area patches of a LULC class in a landscape, where a core area patch has no neighbouring cells of another LULC class. | Zero |
| Normalised Landscape Shape Index | NLSI | None | Characterises the ratio of the actual edge length of a given LULC class to the theoretical range of edge lengths if that class was minimally or maximally aggregated. | NA |
| Number of patches | NP | None | Number of patches of a LULC class in a landscape. | Zero |
| Perimeter-Area Fractal Dimension | PAFRAC | None | Characterises the patch complexity of a specific LULC class by dividing two by the slope of the relationship between the area and perimeter of that class. | NA |
| Coefficient of variation of the perimeter-area ratio | PARA_CV | None | Coefficient of variation of the perimeter to area ratio for all patches belonging to a given LULC class. | NA |
| Mean of the perimeter-area ratio | PARA_MN | None | Mean of the perimeter to area ratio for all patches belonging to a given LULC class. | NA |
| Standard deviation of the perimeter-area ratio | PARA_SD | None | Standard deviation of the perimeter to area ratio for all patches belonging to a given LULC class. | NA |
| Patch density | PD | Number per square kilometre | The number of patches of a given LULC class in relation to the total area of the landscape. | Zero |
| Percentage of Like Adjacencies | PLADJ | Percent | Quantifies how often grid cells of a given LULC class are adjacent to one another. | NA |
| Percentage of landscape of class | PLAND | Percent | The percentage of a landscape covered by a specific LULC class. | Zero |
| Coefficient of variation of shape index | SHAPE_CV | None | Coefficient of variation of the shape index for all patches of a given LULC class, where the shape index is the ratio of the perimeter of a patch and the square root of patch area. | NA |
| Mean of shape index | SHAPE_MN | None | Mean of the shape index for all patches of a given LULC class, where the shape index is the ratio of the perimeter of a patch and the square root of patch area. | NA |
| Standard deviation of shape index | SHAPE_SD | None | Standard deviation of the shape index for all patches of a given LULC class, where the shape index is the ratio of the perimeter of a patch and the square root of patch area. | NA |
| Splitting index | SPLIT | None | Gives the hypothetical number of patches in a landscape if all the patches of a specific LULC class were split into patches of equal size. | NA |
| Total core area | TCA | Square kilometres | Total core area of a LULC class in a landscape, where core area is made up of grid cells with no neighbouring cells of a different LULC class. | Zero |
| Total edge length | TE | Kilometres | Total edge length of a LULC class in a landscape. | Zero |

**Table S 1** Description of all landscape metrics tested for predictable scaling relationships with increasing landscape extent across Colombia. All landscape metrics were quantified using the ‘landscapemetrics’ R package (Hesselbarth et al. 2019), which gives further information as to how each metric is calculated. Description of landscape metrics based on Hesselbarth et al. (2019). LULC = land use and land cover. Treatment of missing values refers to the value used for that metric to represent that a LULC class was missing from a landscape. ‘NA’ indicates that it was not possible to calculate that metric when a LULC class was missing from a landscape. ‘Zero’ indicates that a value of 0 was used in calculations for that metric when a LULC class was missing from a landscape; for example, class area would be equal to 0 where a LULC class is not present in a landscape


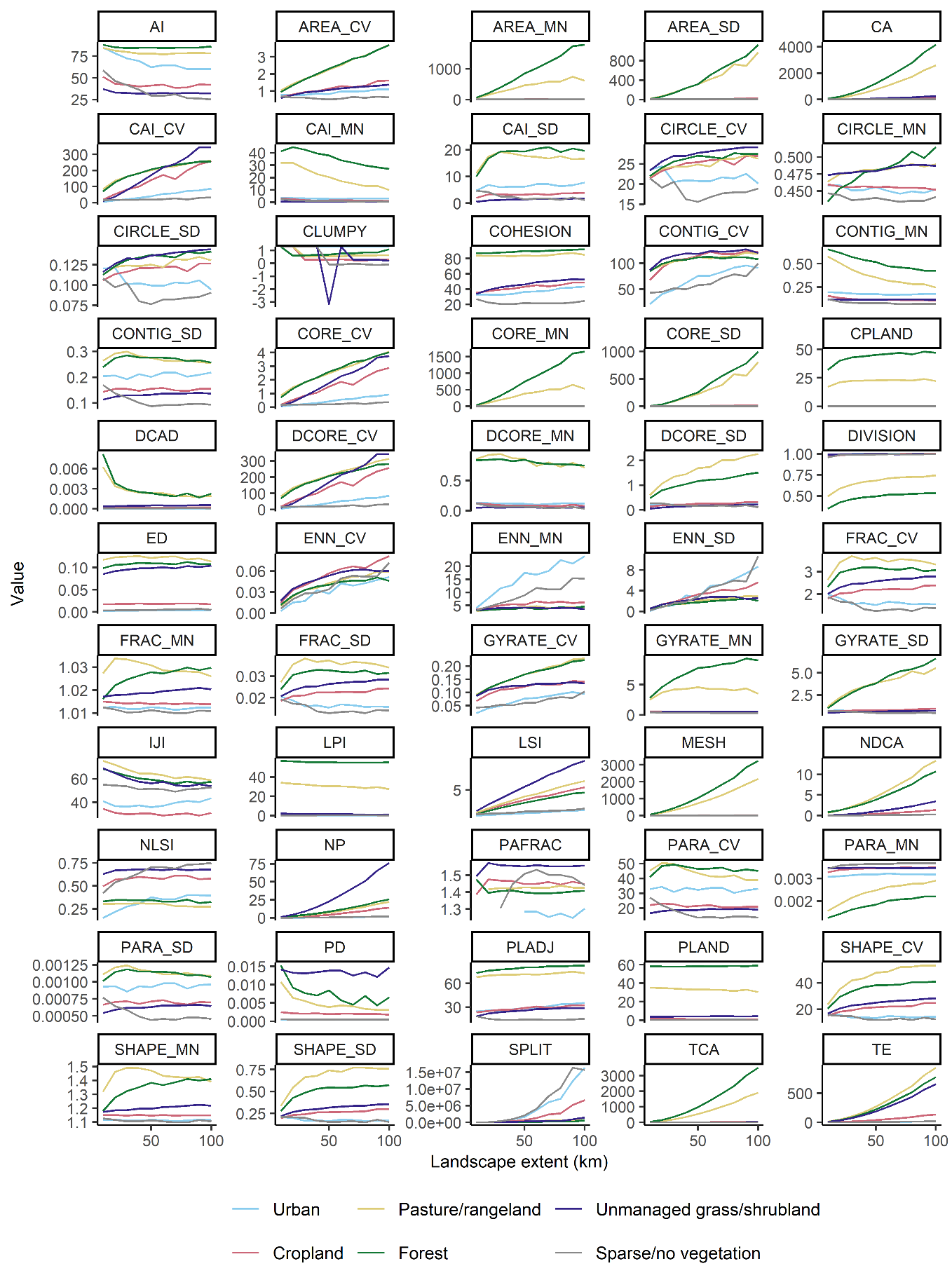


**Fig. S 1** Scaling relationships of landscape metrics with increasing landscape extent for 55 class-level metrics in Colombia in 1992. Landscape extent is the length of each side of a landscape in kilometres. All landscape metric definitions and units are described in Table S 1 and further information can be found in Hesselbarth et al. (2019). Lines give the mean value of a metric across landscapes of different extents for one land use and land cover class


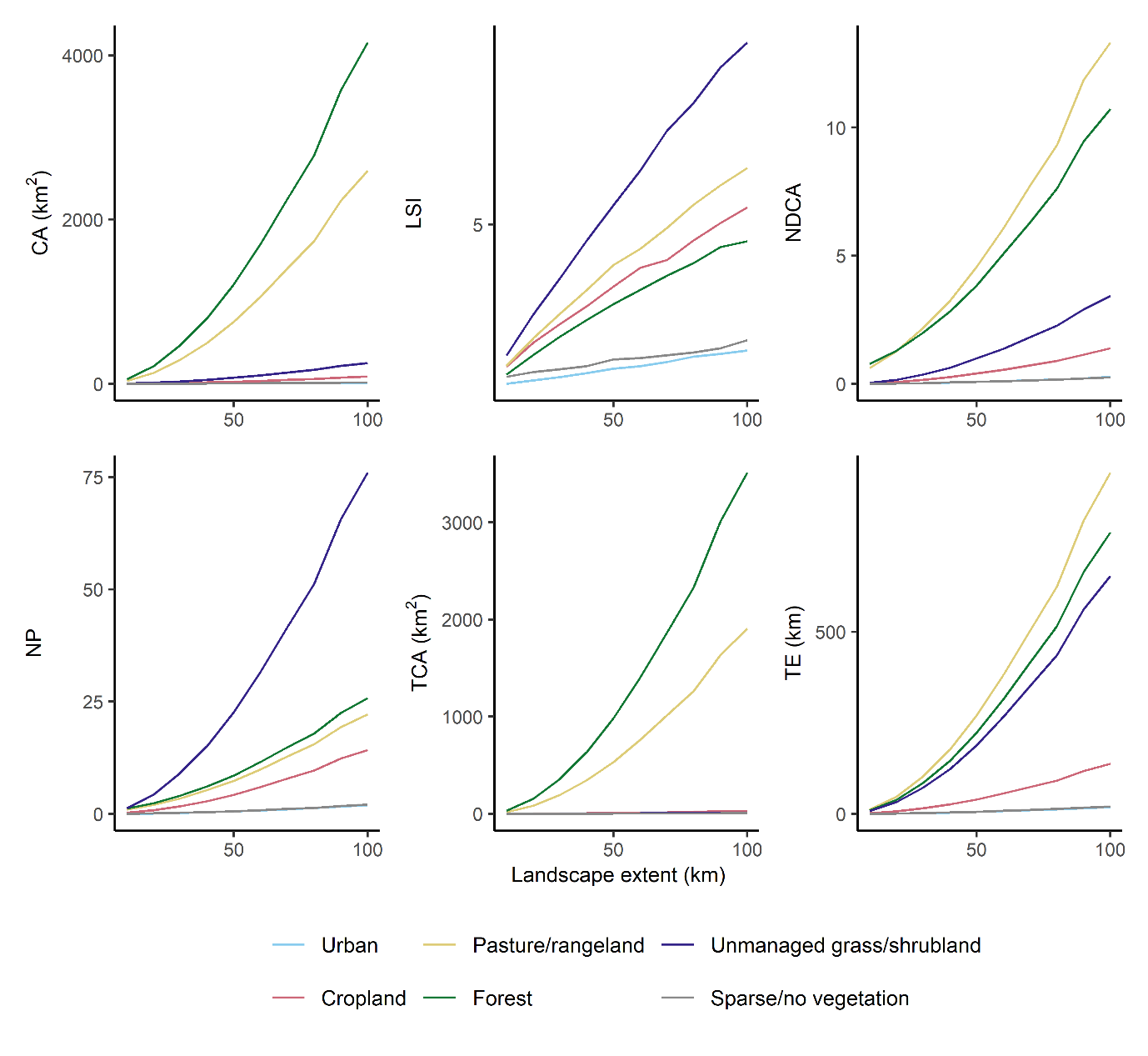


**Fig. S 2** Scaling relationships for six landscape metrics with increasing landscape extent across Colombia. All six metrics showed consistent scaling across increasing landscape extent in Colombia in 1992. Landscape extent is the length of each side of a landscape in kilometres. Lines give the mean value of a metric across landscapes of different extents for one land use and land cover class. See Fig. 2 for definitions of landscape metrics


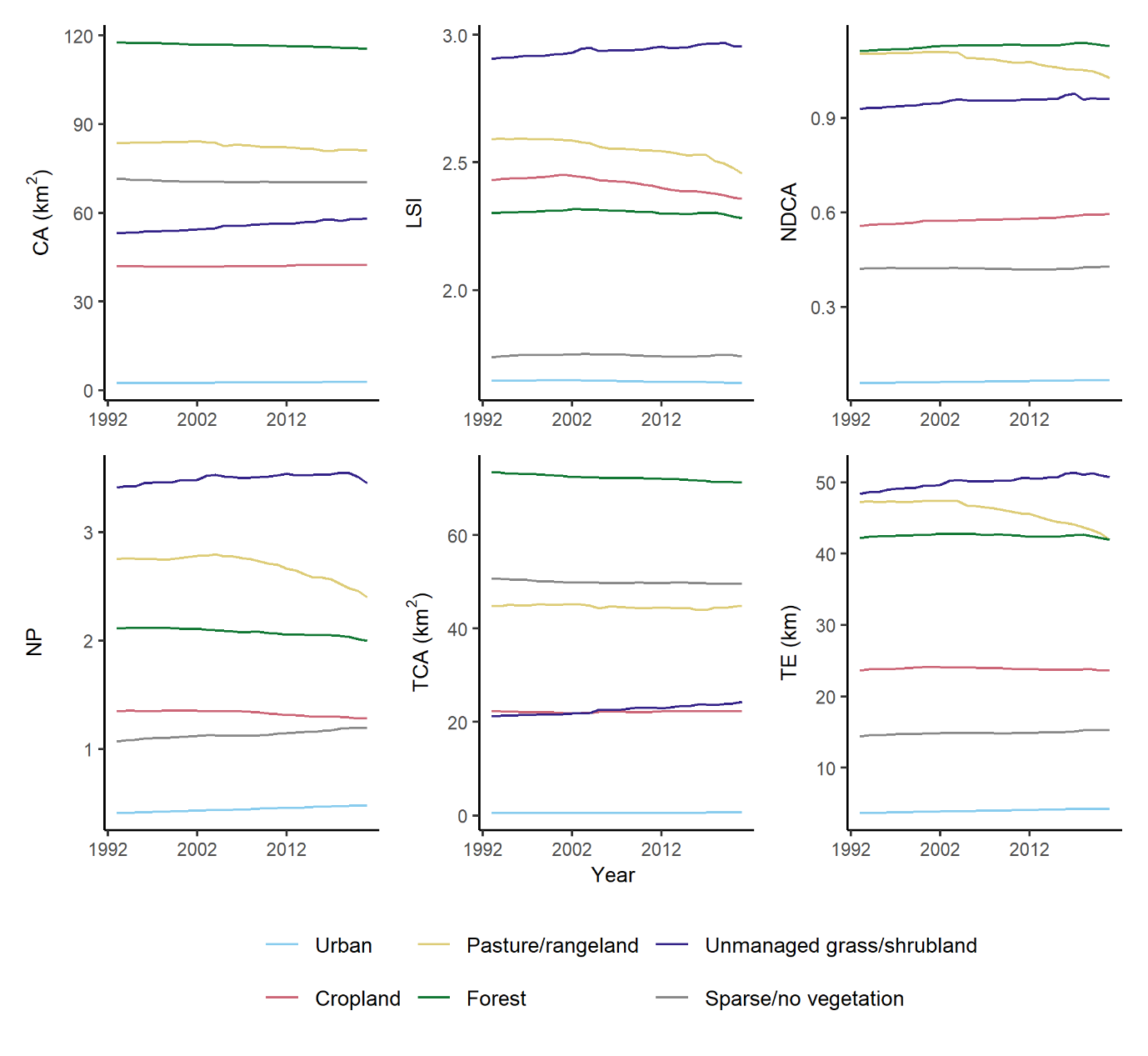


**Fig. S 3** Average of global-scale landscape metrics in 400 km^2^ extent landscapes from 1992 to 2020. Lines give the mean of one landscape metric for one land use and land cover class across 400 km^2^ landscapes in every year from 1992 to 2020. See Fig. 2 for landscape metric definitions and units. Standard deviations are plotted separately in Fig. S 7


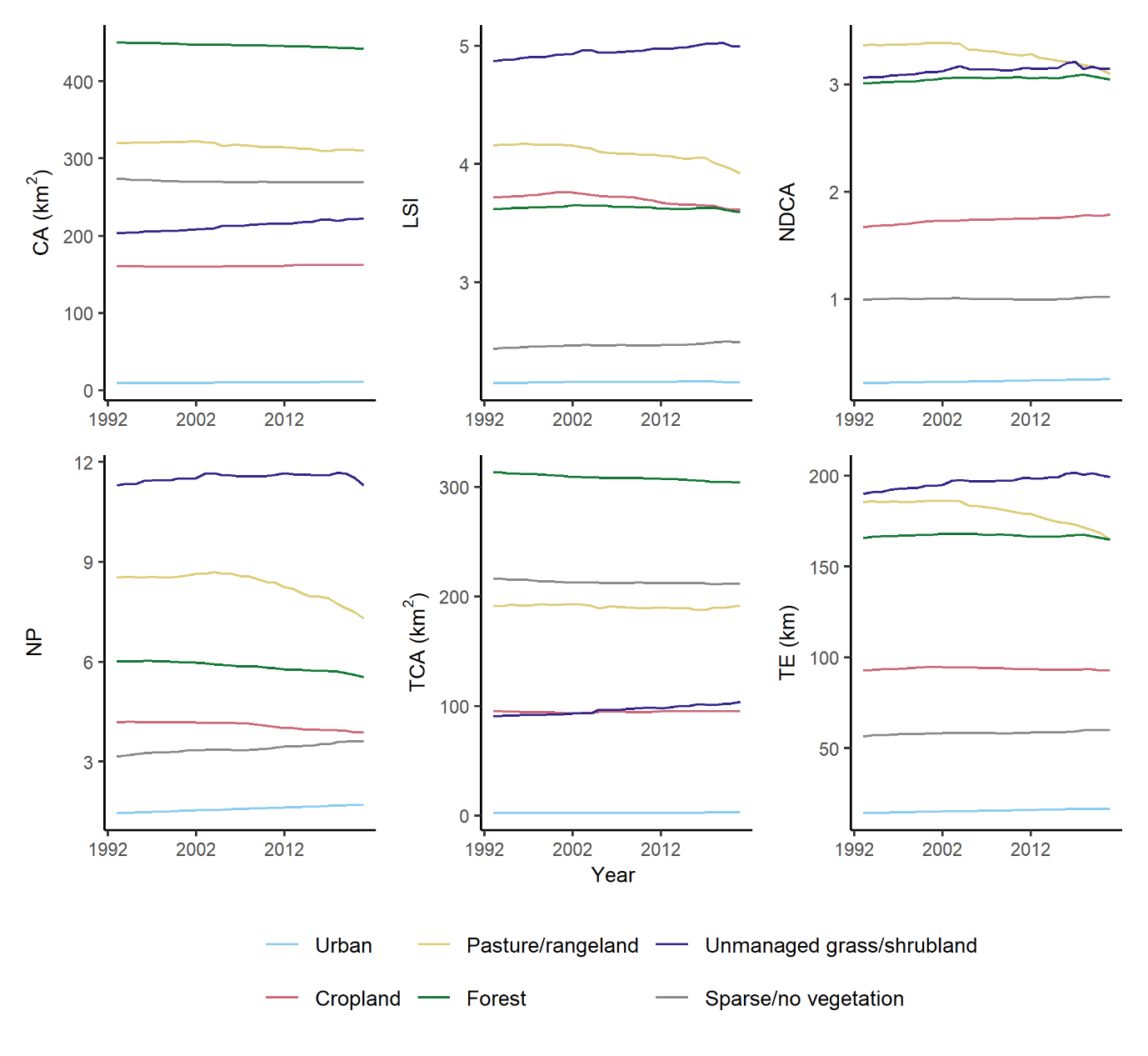


**Fig. S 4** Average of global-scale landscape metrics in 1600 km^2^ extent landscapes from 1992 to 2020. Lines give the mean of one landscape metric for one land use and land cover class across 1600 km^2^ landscapes in every year from 1992 to 2020. See Fig. 2 for landscape metric definitions and units. Standard deviations are plotted separately in Fig. S 8


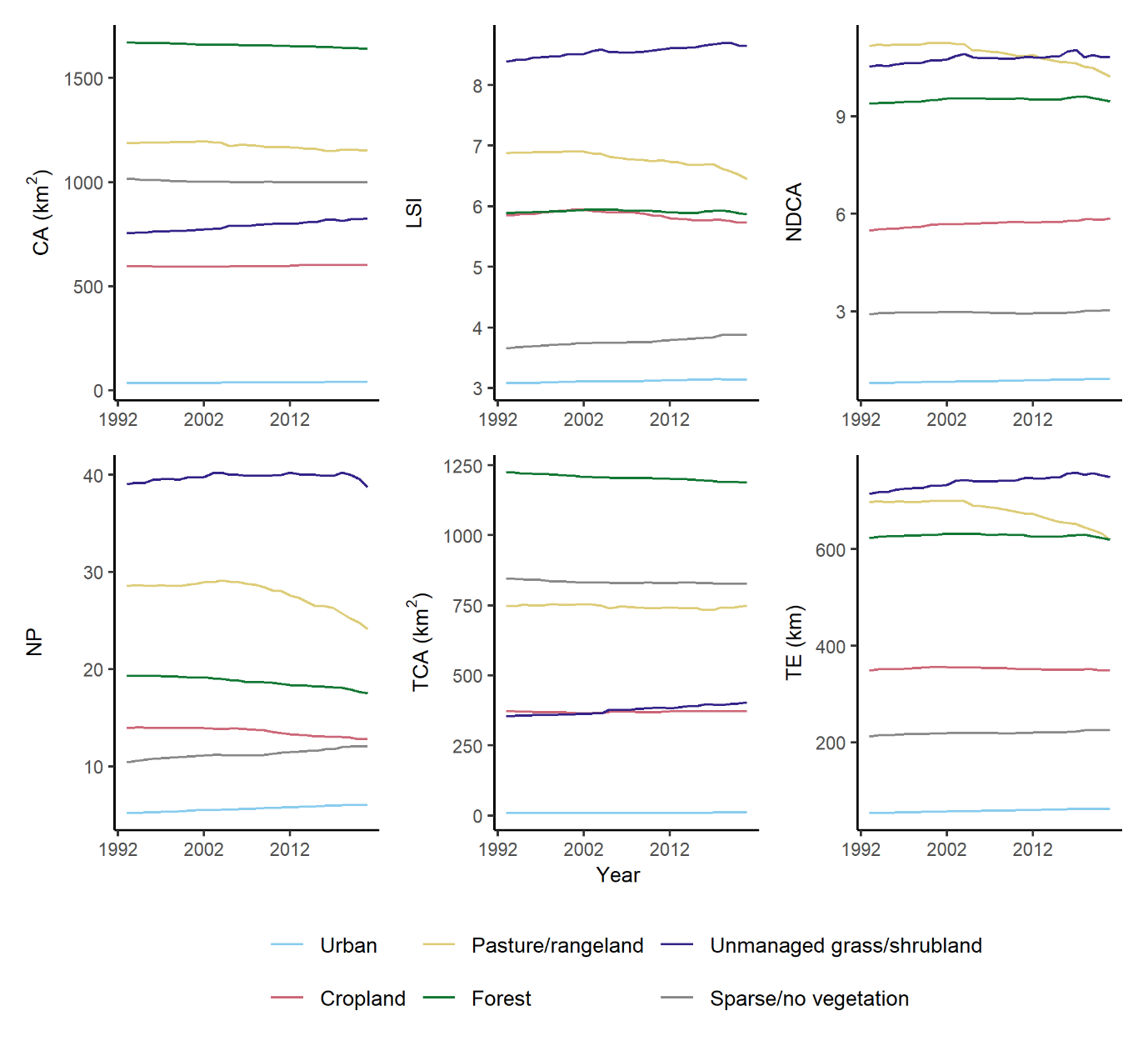


**Fig. S 5** Average of global-scale landscape metrics in 6400 km^2^ extent landscapes from 1992 to 2020. Lines give the mean of one landscape metric for one land use and land cover class across 6400 km^2^ landscapes in every year from 1992 to 2020. See Fig. 2 for landscape metric definitions and units. Standard deviations are plotted separately in Fig. S 9


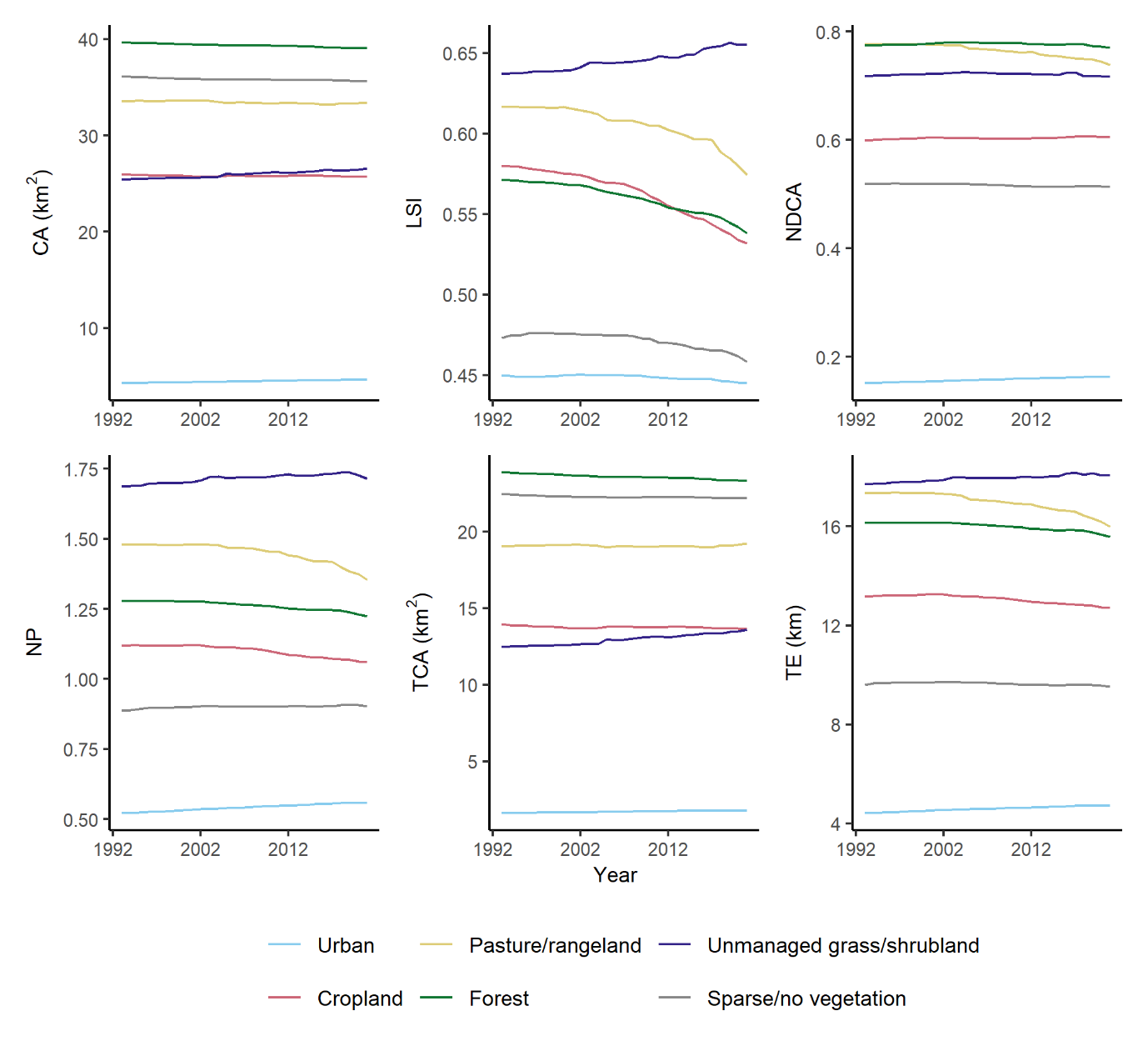


**Fig. S 6** Global-scale standard deviations in landscape metrics in 100 km^2^ extent landscapes from 1992 to 2020. Lines give the standard deviation of one landscape metric for one land use and land cover class across landscapes of 100 km^2^ extent in every year from 1992 to 2020. See Fig. 2 for landscape metric definitions and units


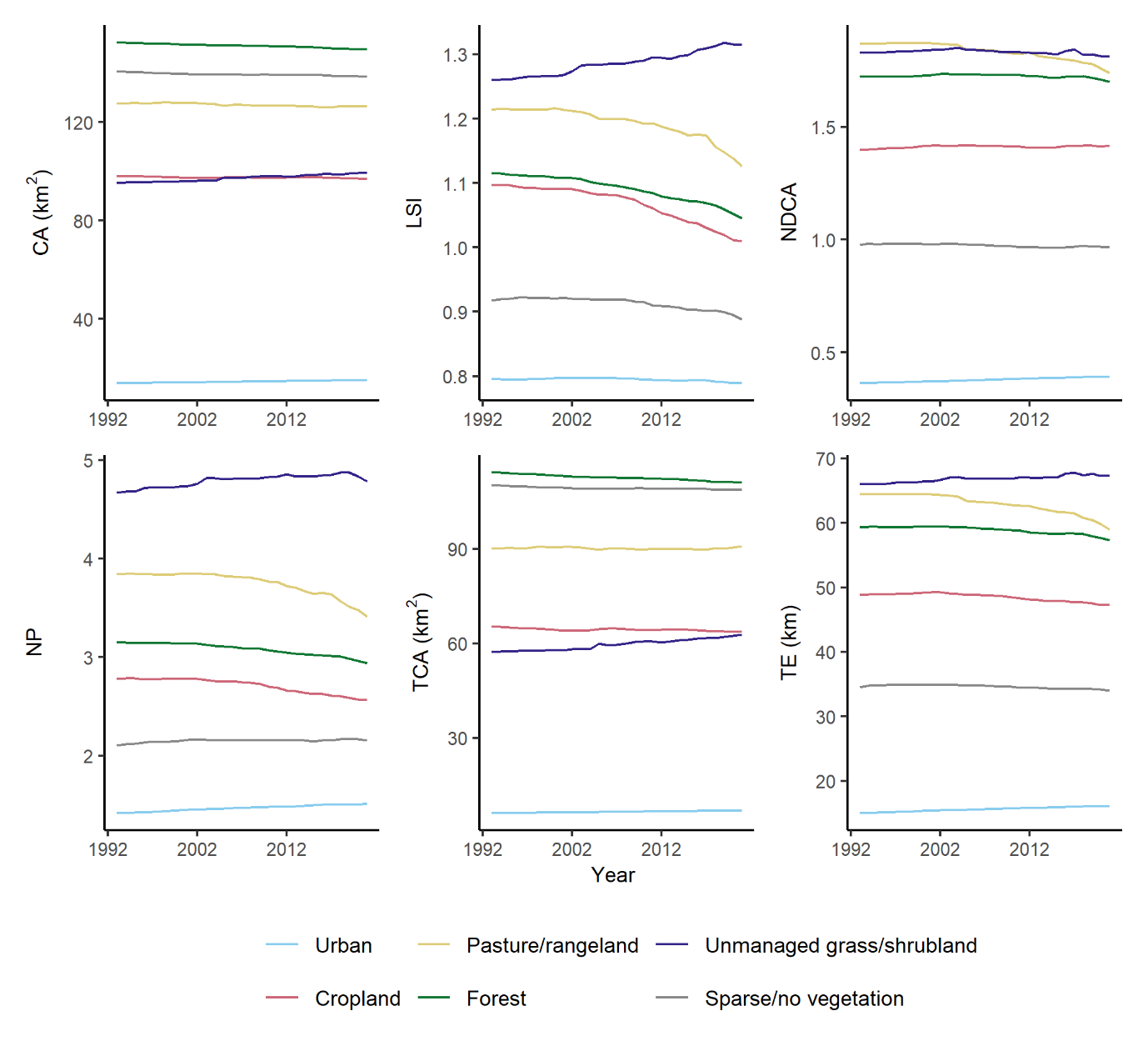


**Fig. S 7** Global-scale standard deviations in landscape metrics in 400 km^2^ extent landscapes from 1992 to 2020. Lines give the standard deviation of one landscape metric for one land use and land cover class across landscapes of 400 km^2^ extent in every year from 1992 to 2020. See Fig. 2 for landscape metric definitions and units


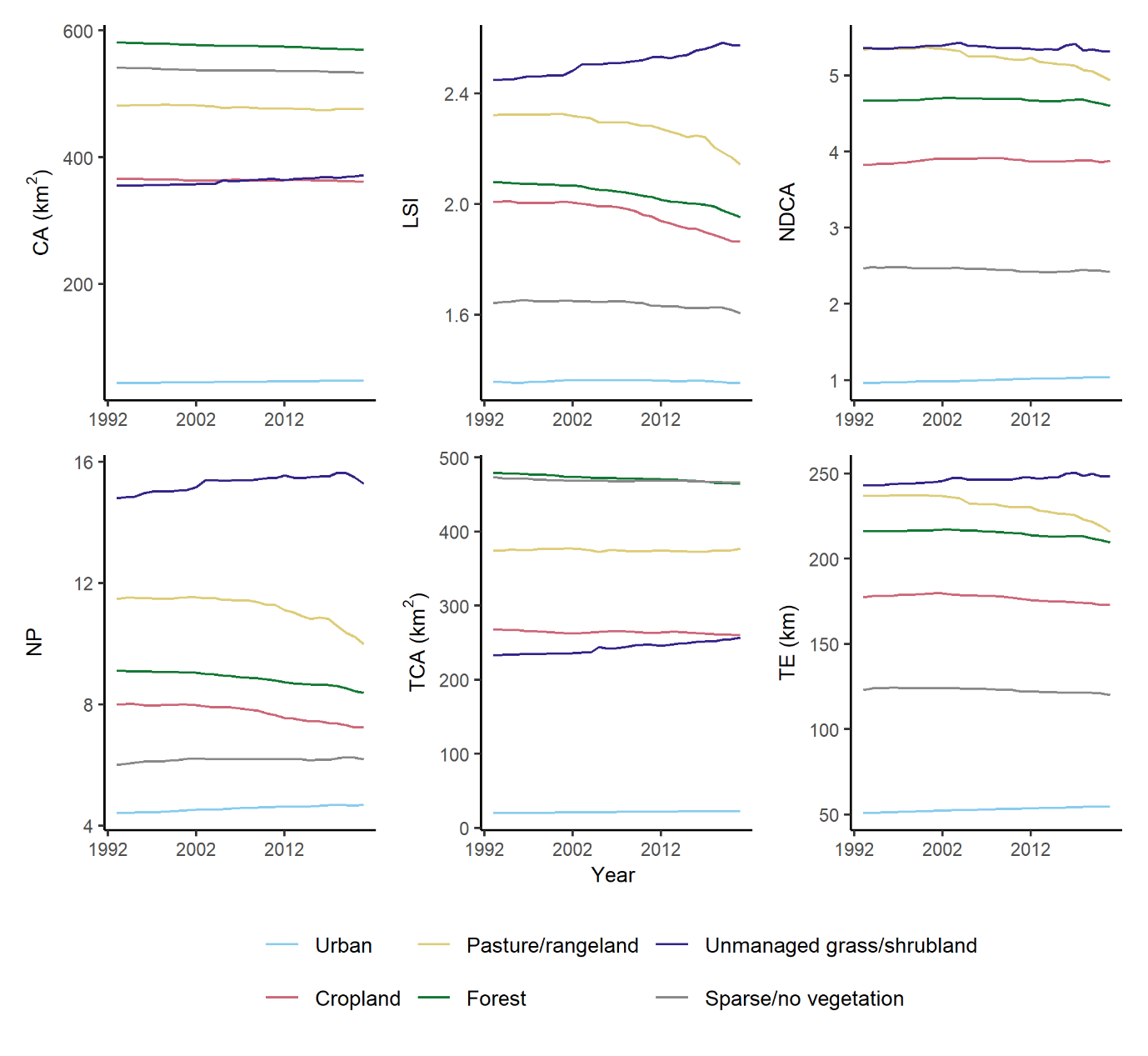


**Fig. S 8** Global-scale standard deviations in landscape metrics in 1600 km^2^ extent landscapes from 1992 to 2020. Lines give the standard deviation of one landscape metric for one land use and land cover class across landscapes of 1600 km^2^ extent in every year from 1992 to 2020. See Fig. 2 for landscape metric definitions and units


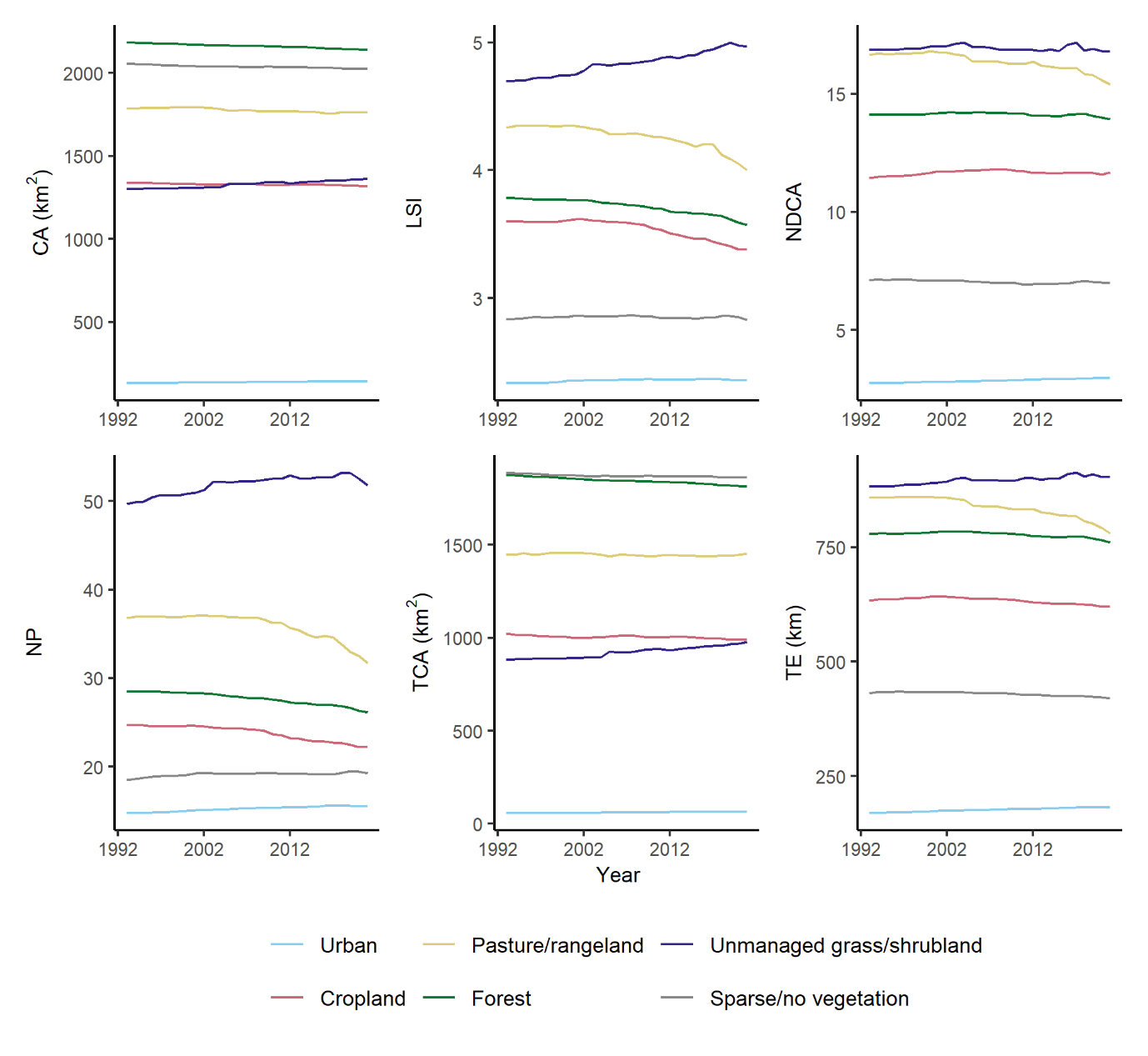


**Fig. S 9** Global-scale standard deviations in landscape metrics in 6400 km^2^ extent landscapes from 1992 to 2020. Lines give the standard deviation of one landscape metric for one land use and land cover class across landscapes of 6400 km^2^ extent in every year from 1992 to 2020. See Fig. 2 for landscape metric definitions and units


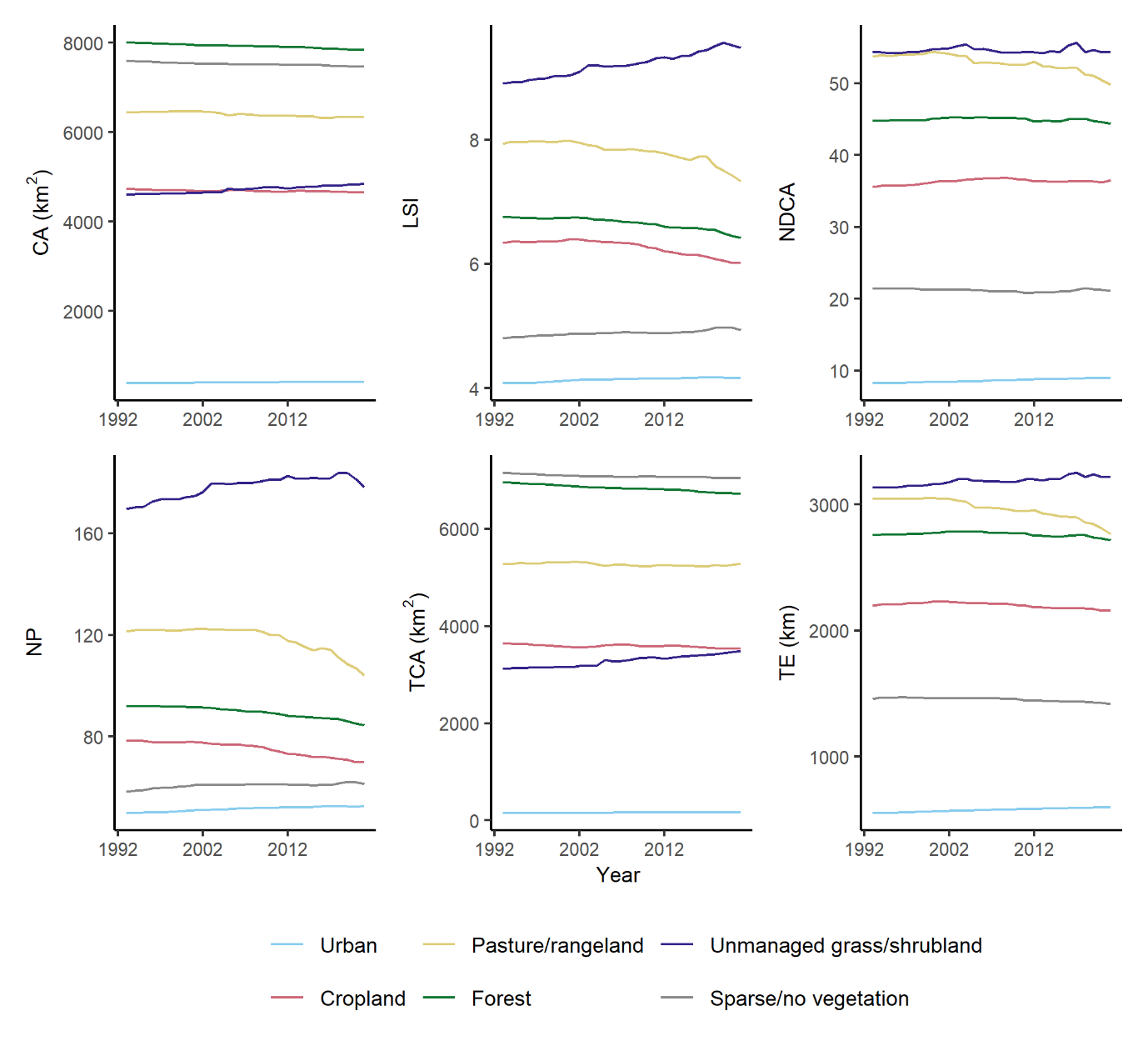


**Fig. S 10** Global-scale standard deviations in landscape metrics in 25600 km^2^ extent landscapes from 1992 to 2020. Lines give the standard deviation of one landscape metric for one land use and land cover class across landscapes of 25600 km^2^ extent in every year from 1992 to 2020. See Fig. 2 for landscape metric definitions and units


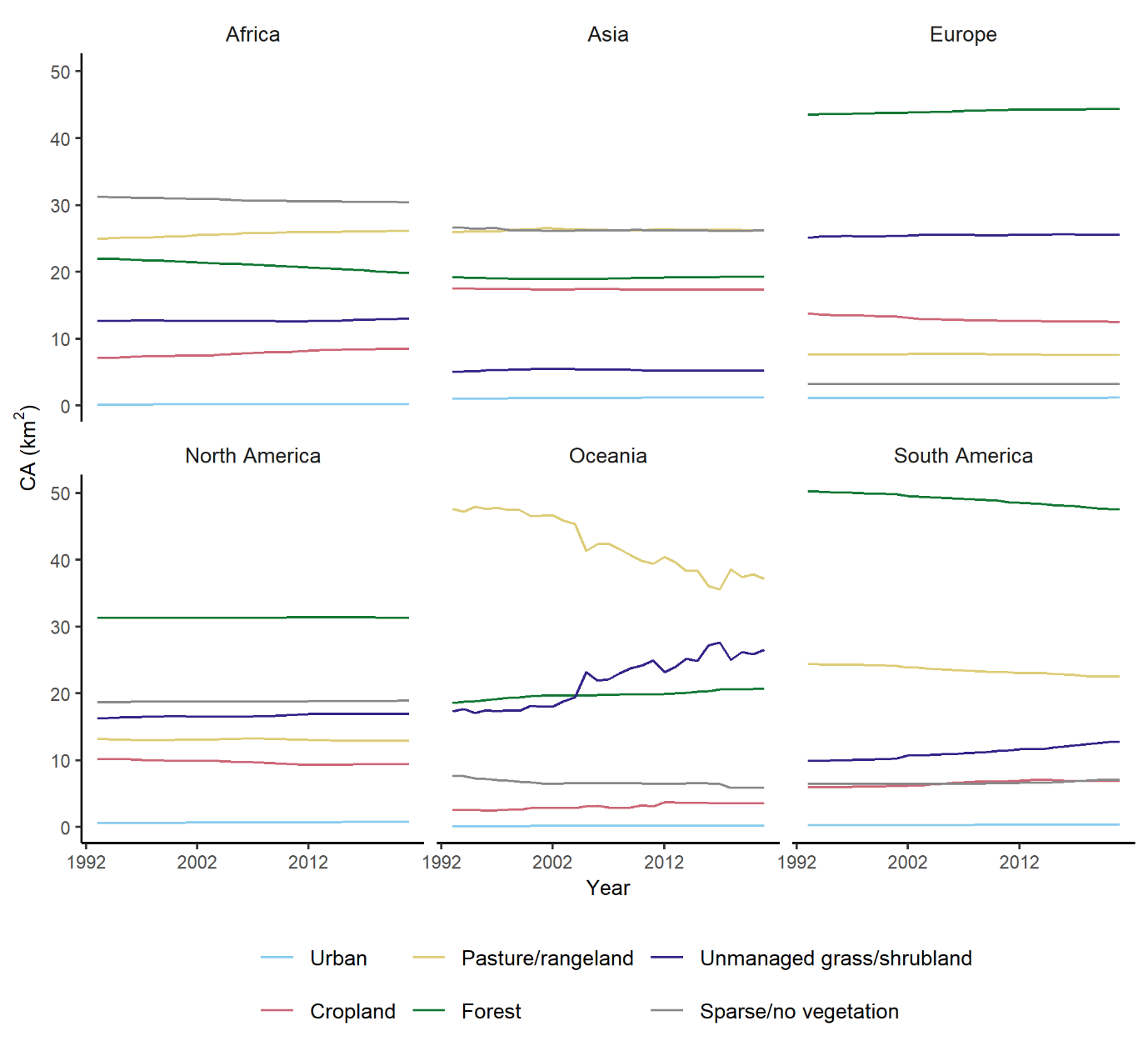


**Fig. S 11** Average class area (CA) at continental scale from 1992 to 2020 in landscapes of 100 km^2^ extent. Lines give the mean of CA for one land use and land cover class. Units for CA are square kilometres (km^2^). Standard deviations are plotted separately in Fig. S 17


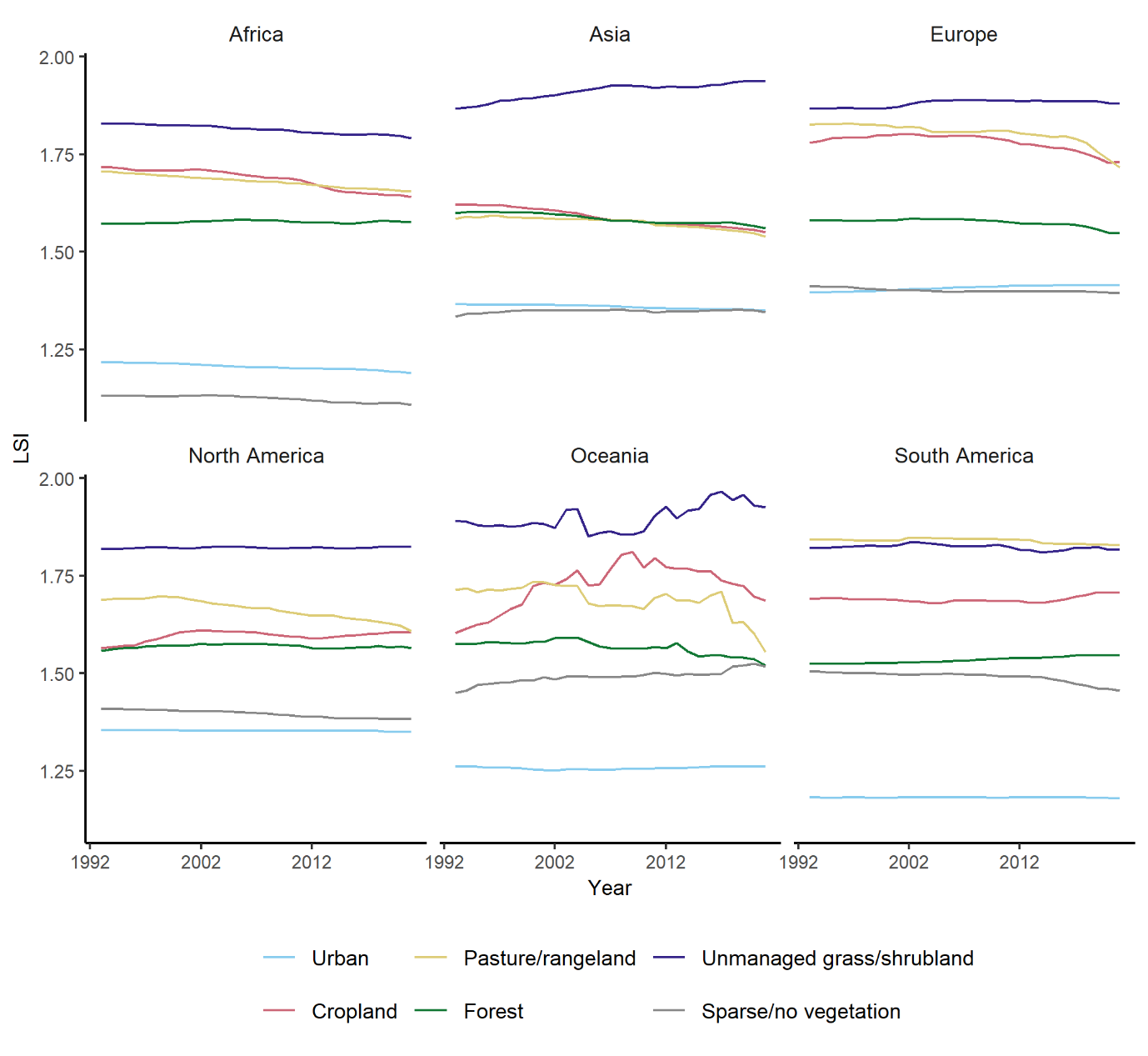


**Fig. S 12** Average Landscape Shape Index (LSI) at continental scale from 1992 to 2020 in landscapes of 100 km^2^ extent. Lines give the mean of LSI for one land use and land cover class. LSI is a unitless measure. Standard deviations are plotted separately in Fig. S 18


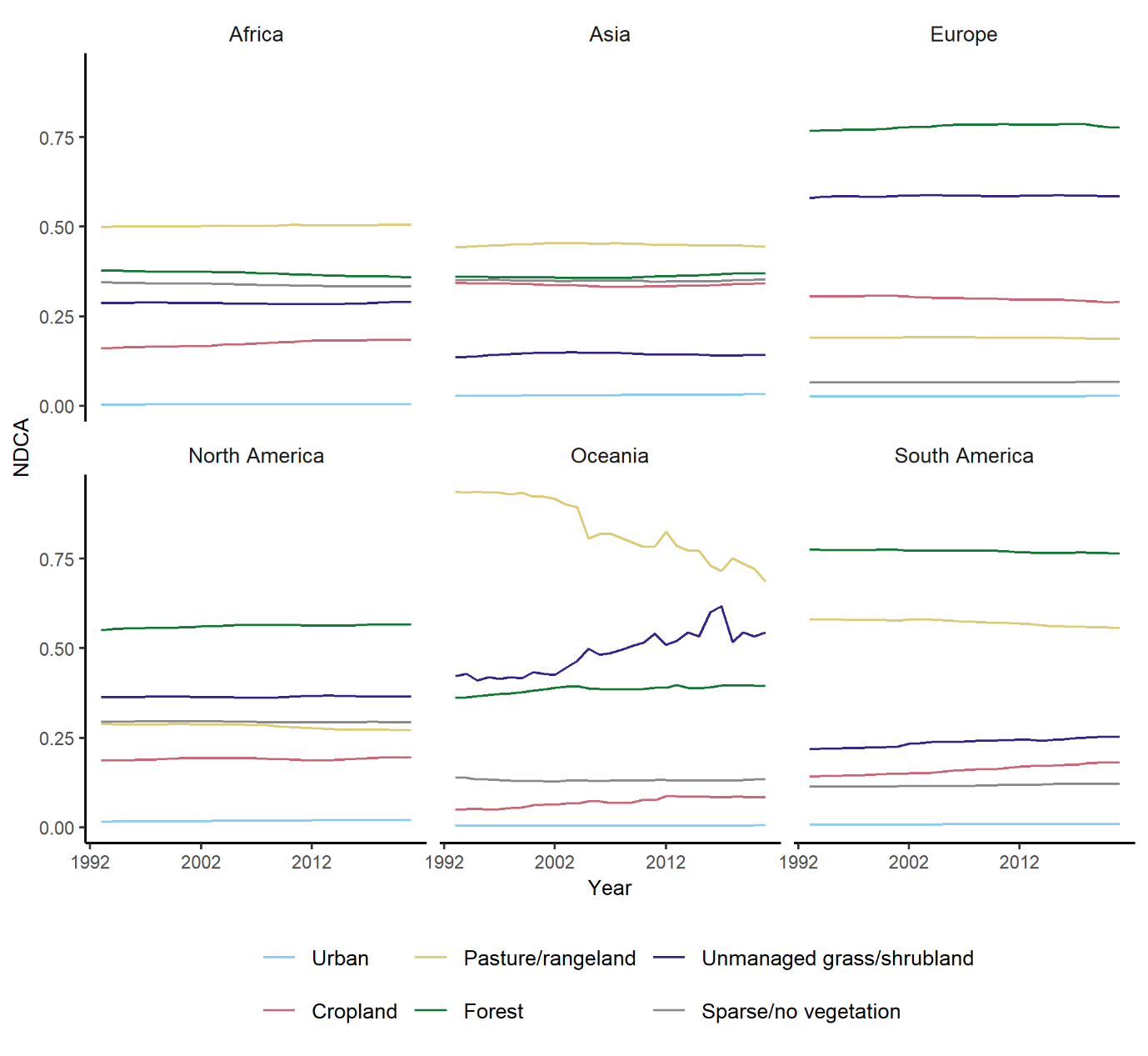


**Fig. S 13** Average number of disjunct core area patches (NDCA) at continental scale from 1992 to 2020 in landscapes of 100 km^2^ extent. Lines give the mean of NDCA for one land use and land cover class. NDCA is a unitless measure. Standard deviations are plotted separately in Fig. S 19


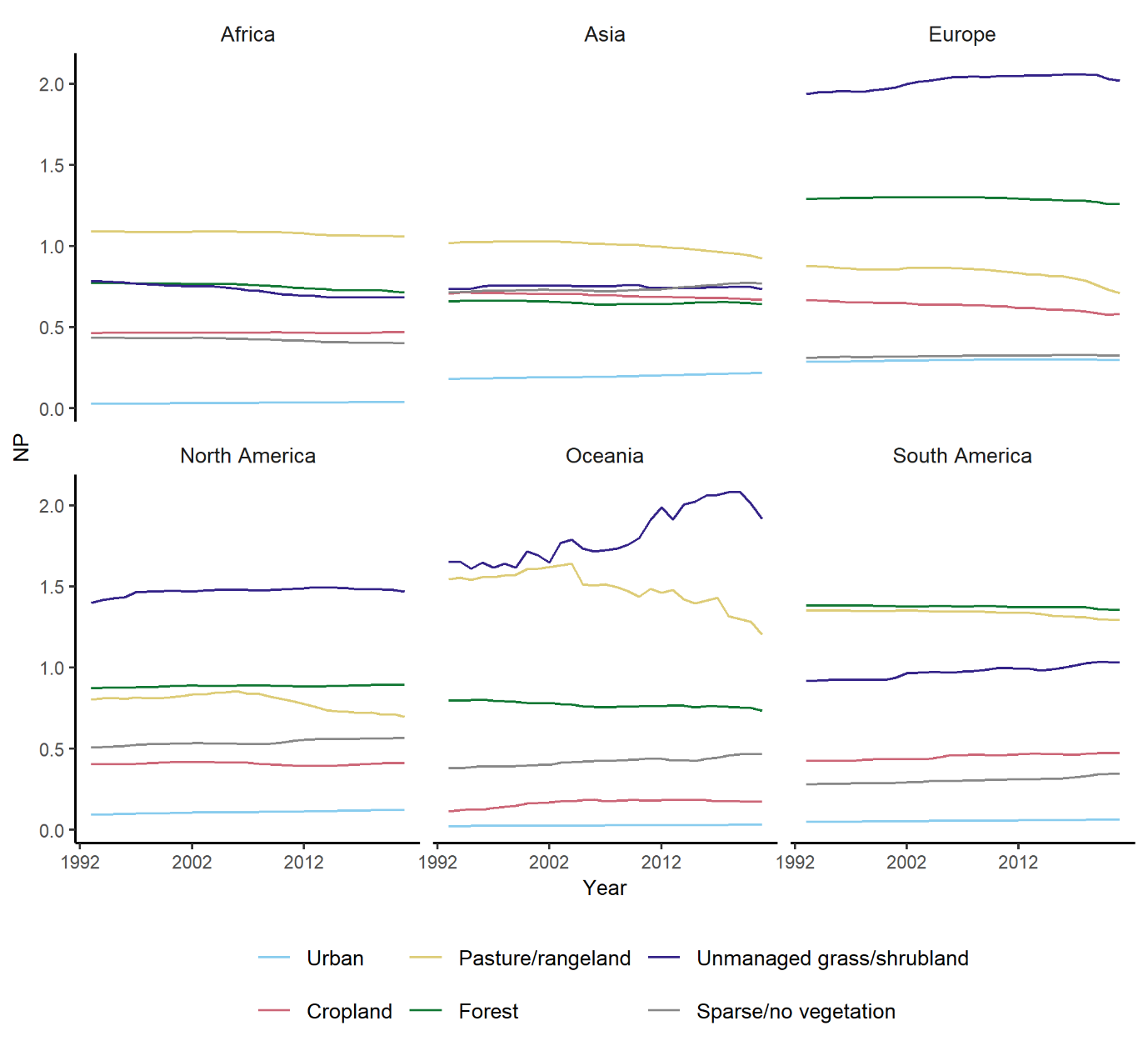


**Fig. S 14** Average number of patches (NP) at continental scale from 1992 to 2020 in landscapes of 100 km^2^ extent. Lines give the mean of NP for one land use and land cover class. NP is a unitless measure. Standard deviations are plotted separately in Fig. S 20


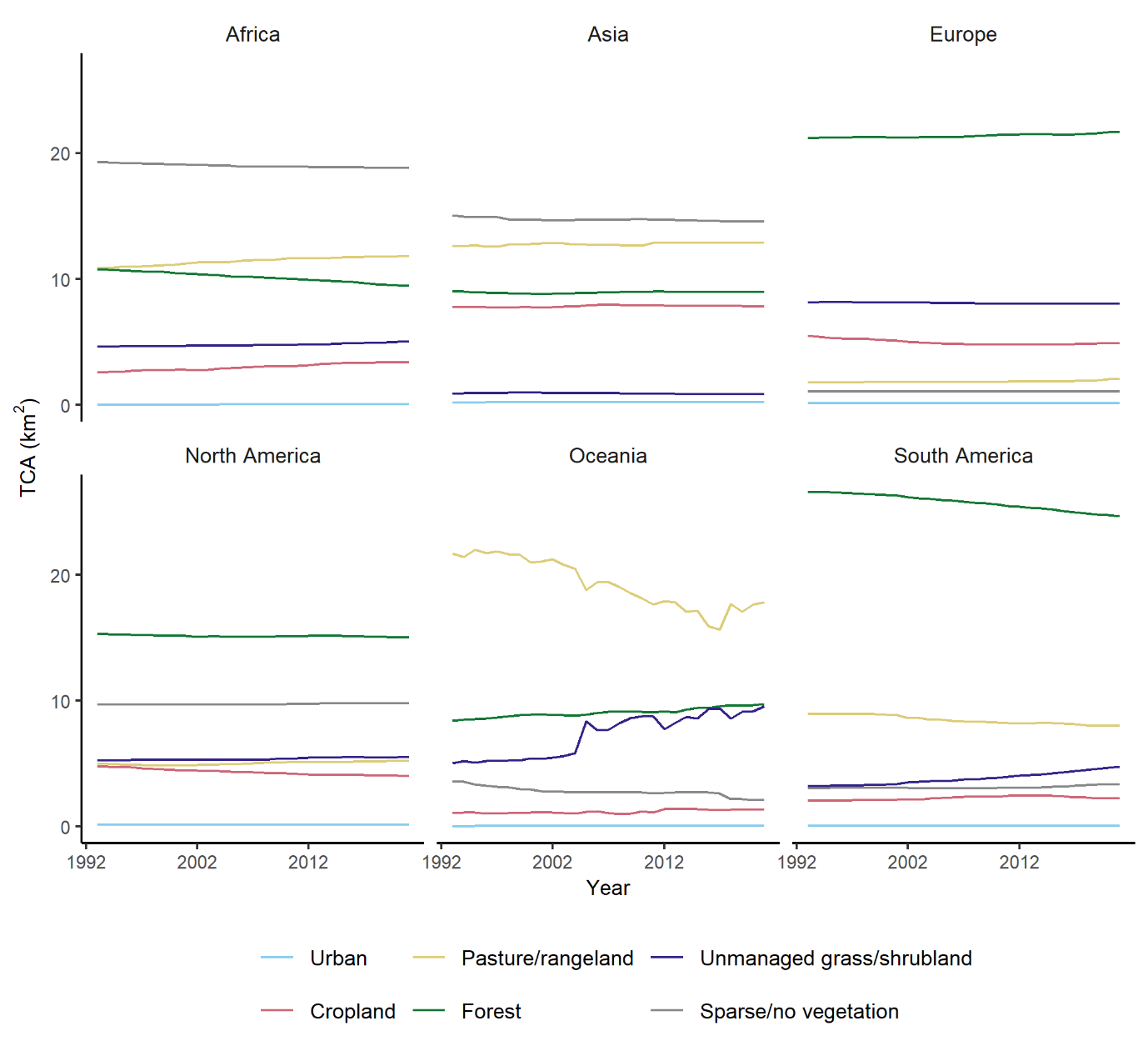


**Fig. S 15** Average total core area (TCA) at continental scale from 1992 to 2020 in landscapes of 100 km^2^ extent. Lines give the mean of TCA for one land use and land cover class. Units for TCA are square kilometres (km^2^). Standard deviations are plotted separately in Fig. S 21


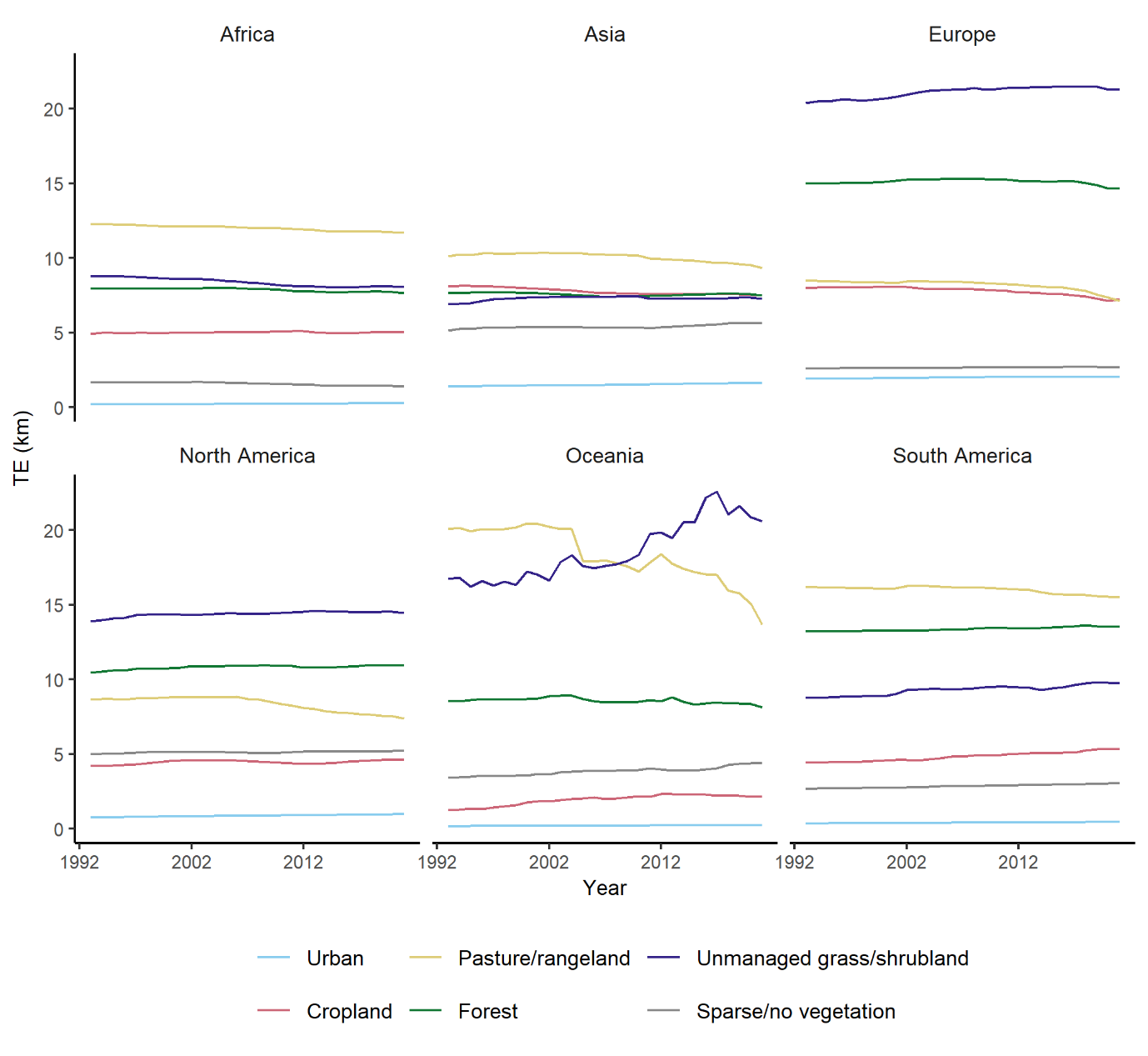


**Fig. S 16** Average total edge length (TE) at continental scale from 1992 to 2020 in landscapes of 100 km^2^ extent. Lines give the mean of TE for one land use and land cover class. Units for TE are kilometres (km). Standard deviations are plotted separately in Fig. S 22


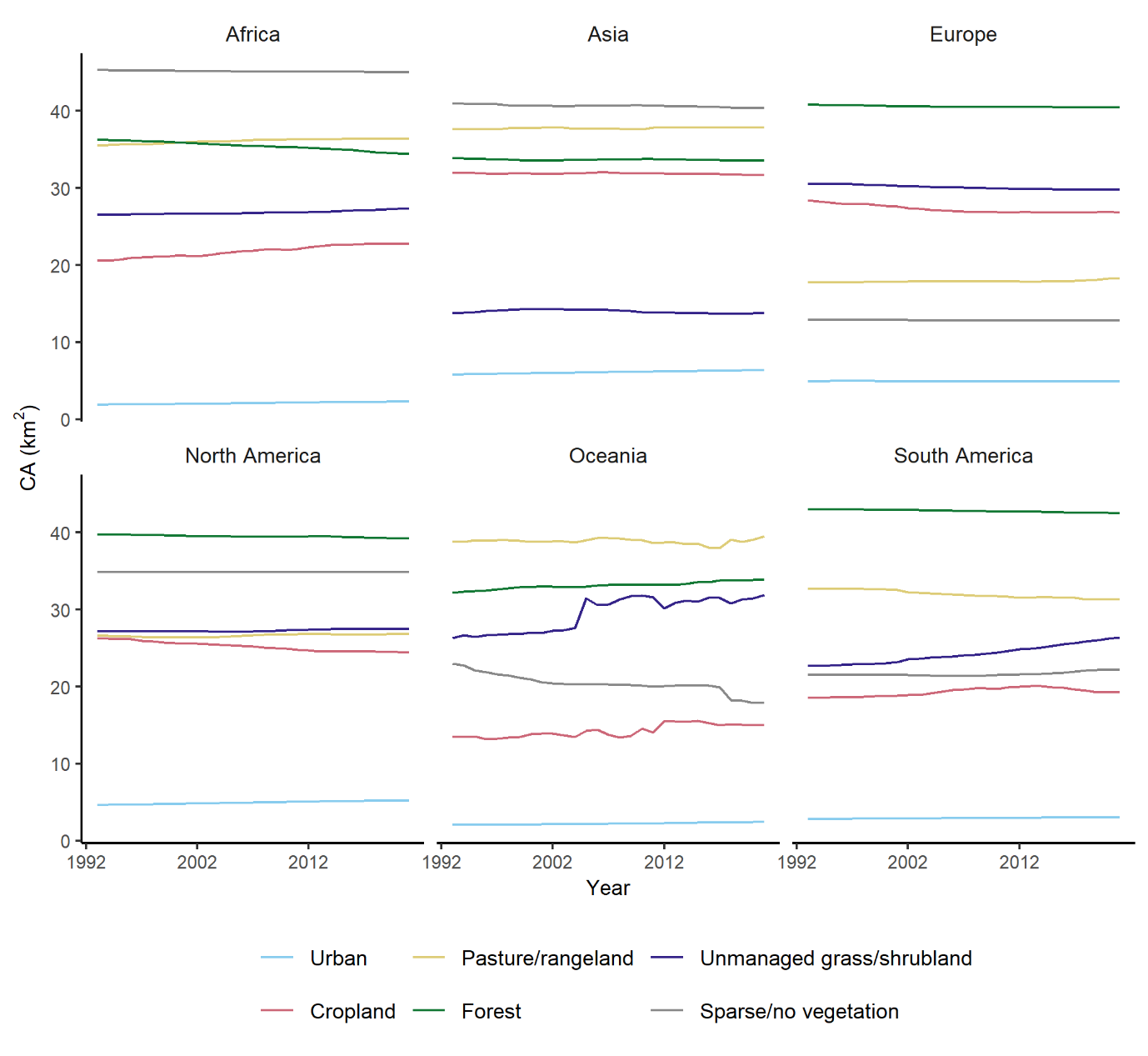


**Fig. S 17** Standard deviation of class area (CA) at continental scale from 1992 to 2020 in landscapes of 100 km^2^ extent. Lines give the standard deviation of CA for one land use and land cover class. Units of CA are square kilometres (km^2^)


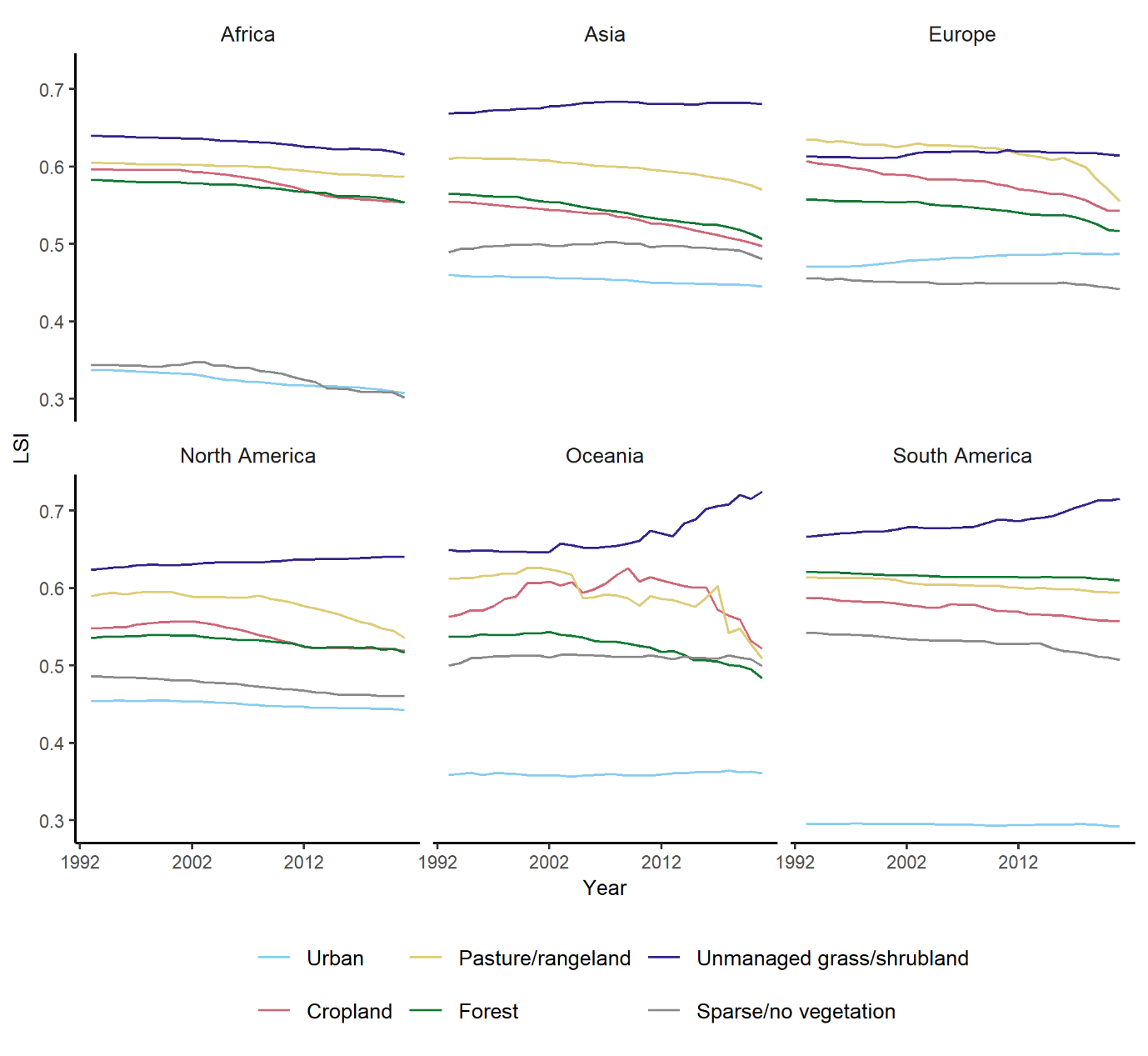


**Fig. S 18** Standard deviation of Landscape Shape Index (LSI) at continental scale from 1992 to 2020 in landscapes of 100 km^2^ extent. Lines give the standard deviation of LSI for one land use and land cover class. LSI is a unitless measure


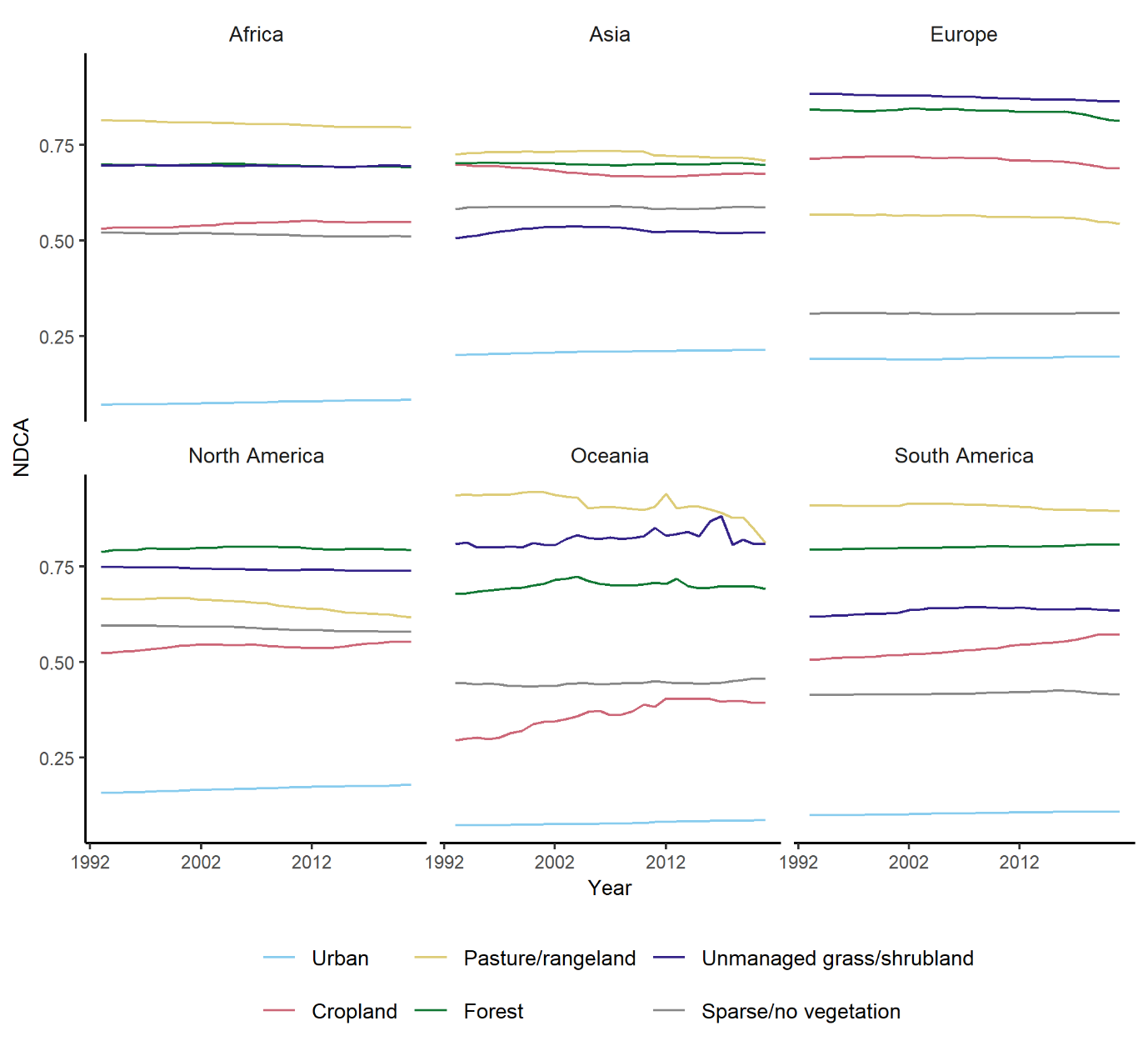


**Fig. S 19** Standard deviation of number of disjunct core area patches (NDCA) at continental scale from 1992 to 2020 in landscapes of 100 km^2^ extent. Lines give the standard deviation of NDCA for one land use and land cover class. NDCA is a unitless measure


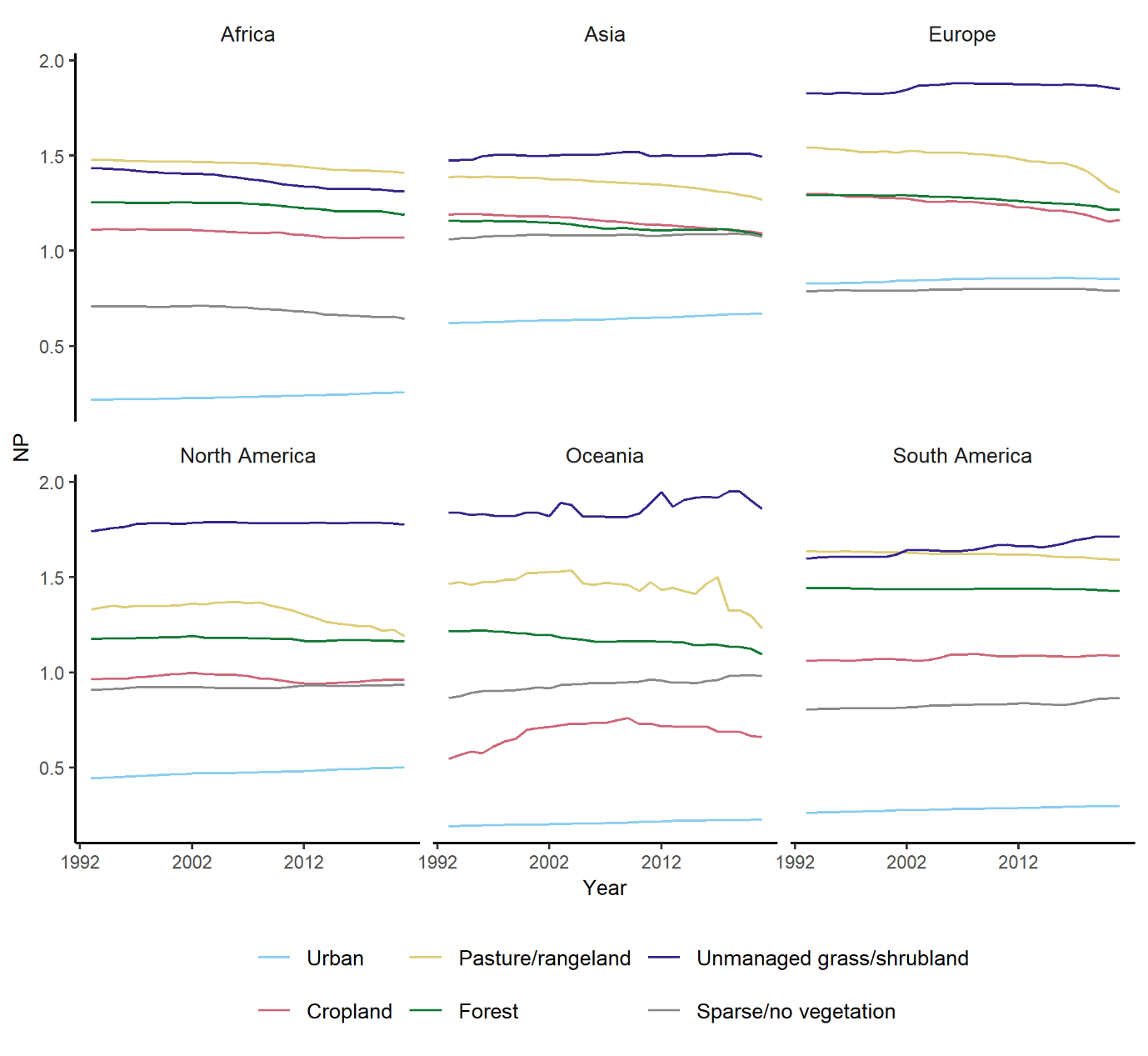


**Fig. S 20** Standard deviation of number of patches (NP) at continental scale from 1992 to 2020 in landscapes of 100 km^2^ extent. Lines give the standard deviation of NP for one land use and land cover class. NP is a unitless measure


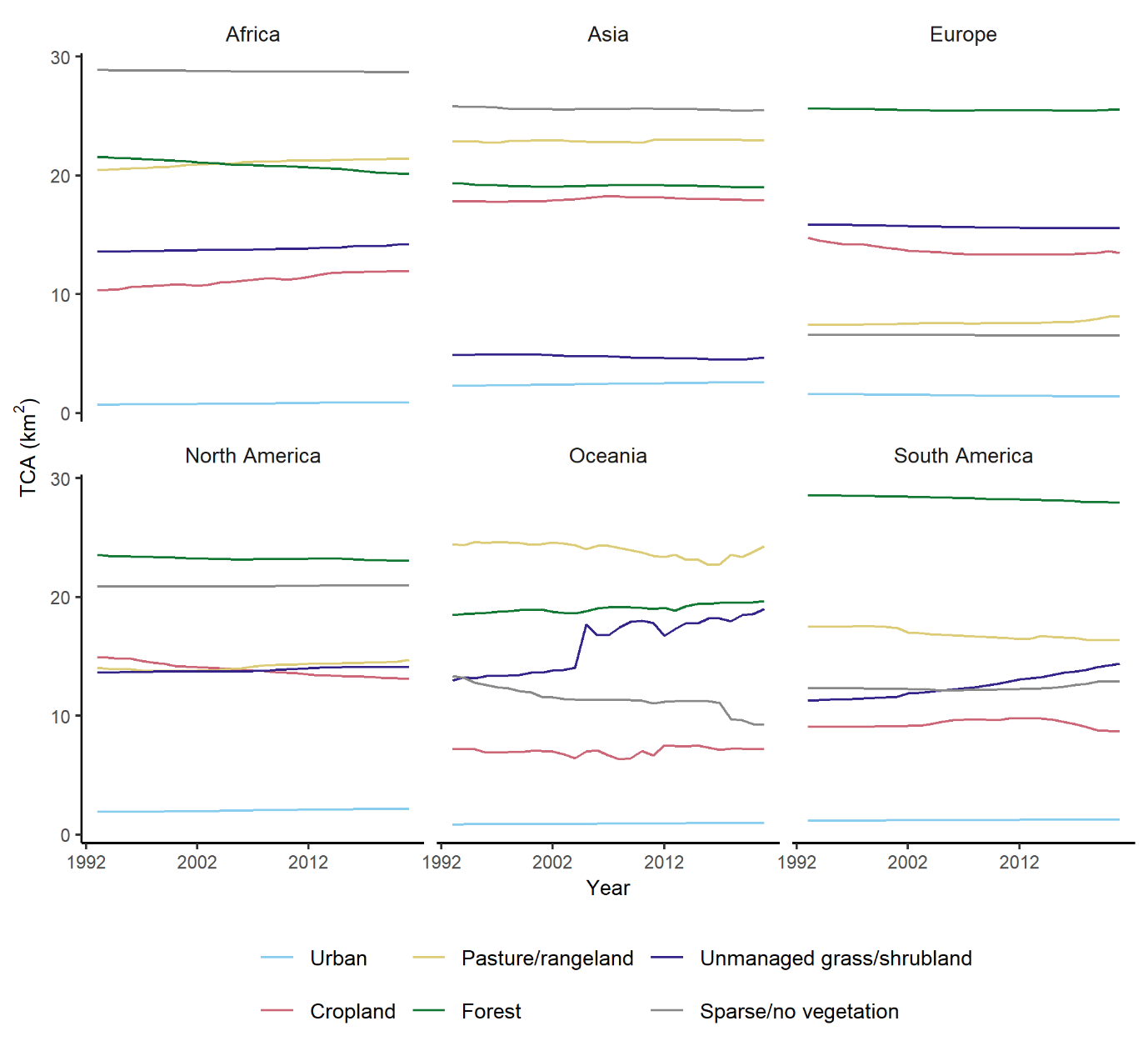


**Fig. S 21** Standard deviation of total core area (TCA) at continental scale from 1992 to 2020 in landscapes of 100 km^2^ extent. Lines give the standard deviation of TCA for one land use and land cover class. Units of TCA are square kilometres (km^2^)


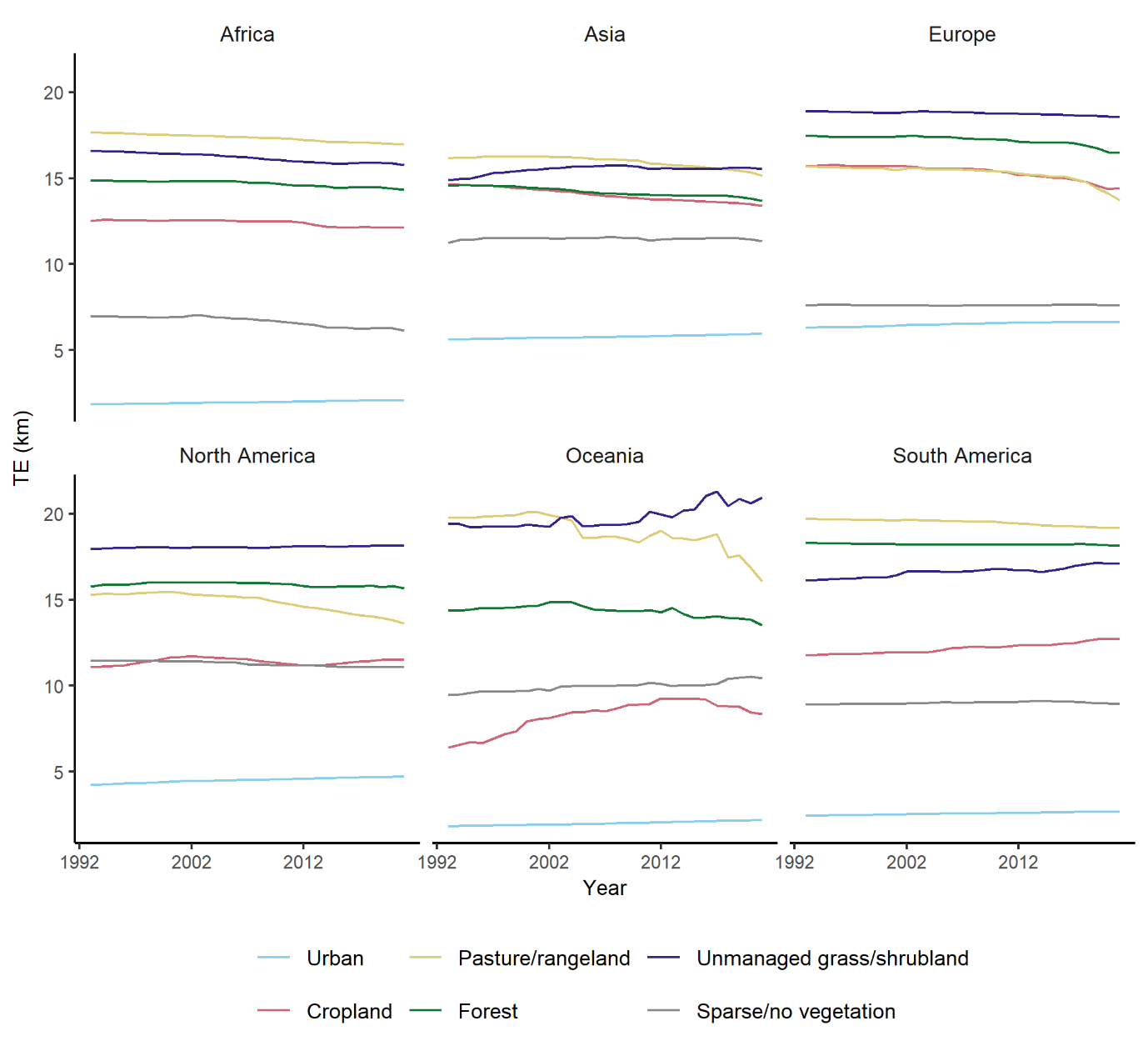


**Fig. S 22** Standard deviation of total edge length (TE) at continental scale from 1992 to 2020 in landscapes of 100 km^2^ extent. Lines give the standard deviation of TE for one land use and land cover class. Units of TE are kilometres (km)


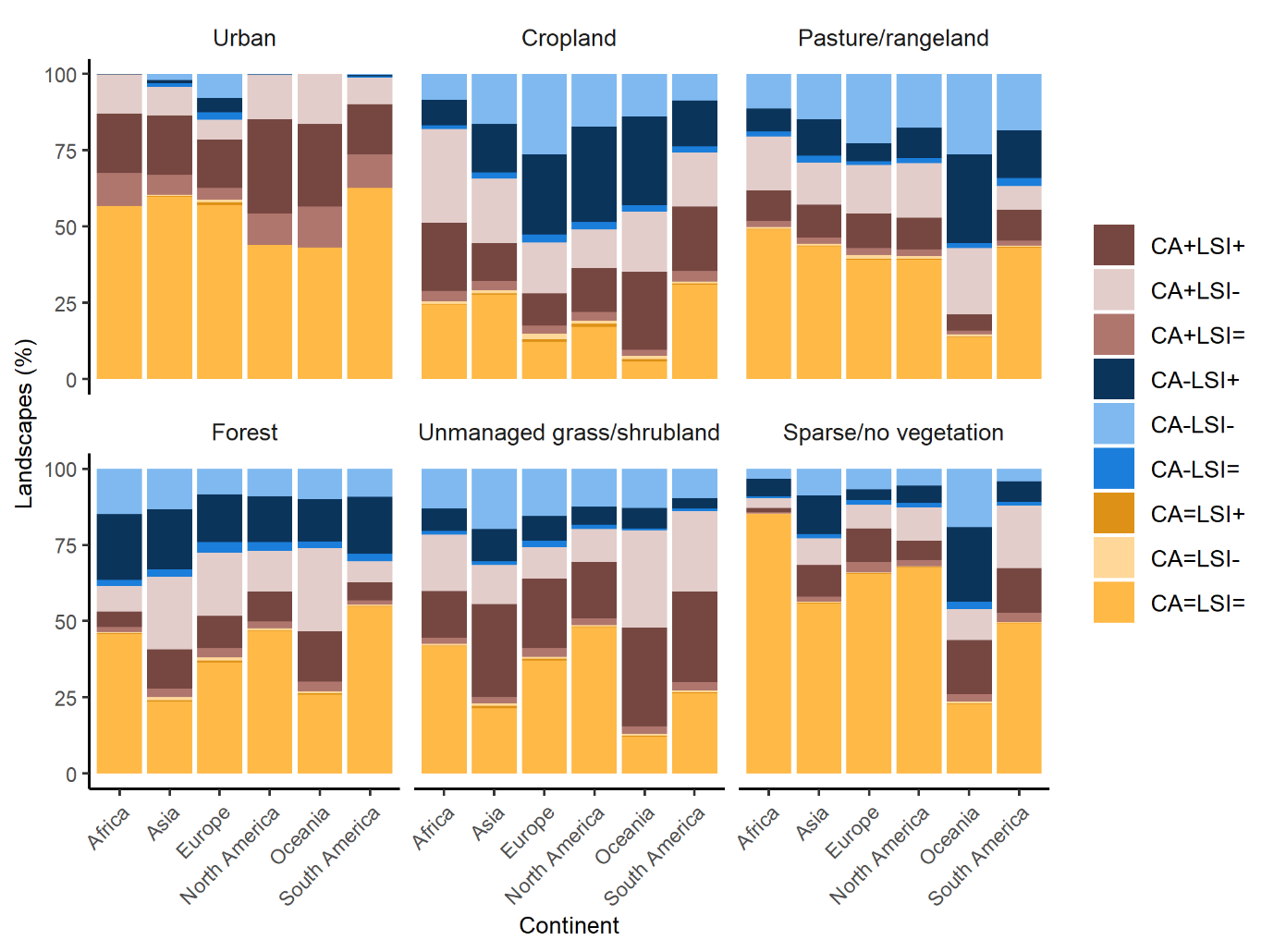


**Fig. S 23** Direction of net change in area and configuration within 100 km^2^ landscapes from 1992 to 2020 at continental scale. Bars give the percentage of landscapes which contained the specific land use and land cover (LULC) class in both 1992 and 2020


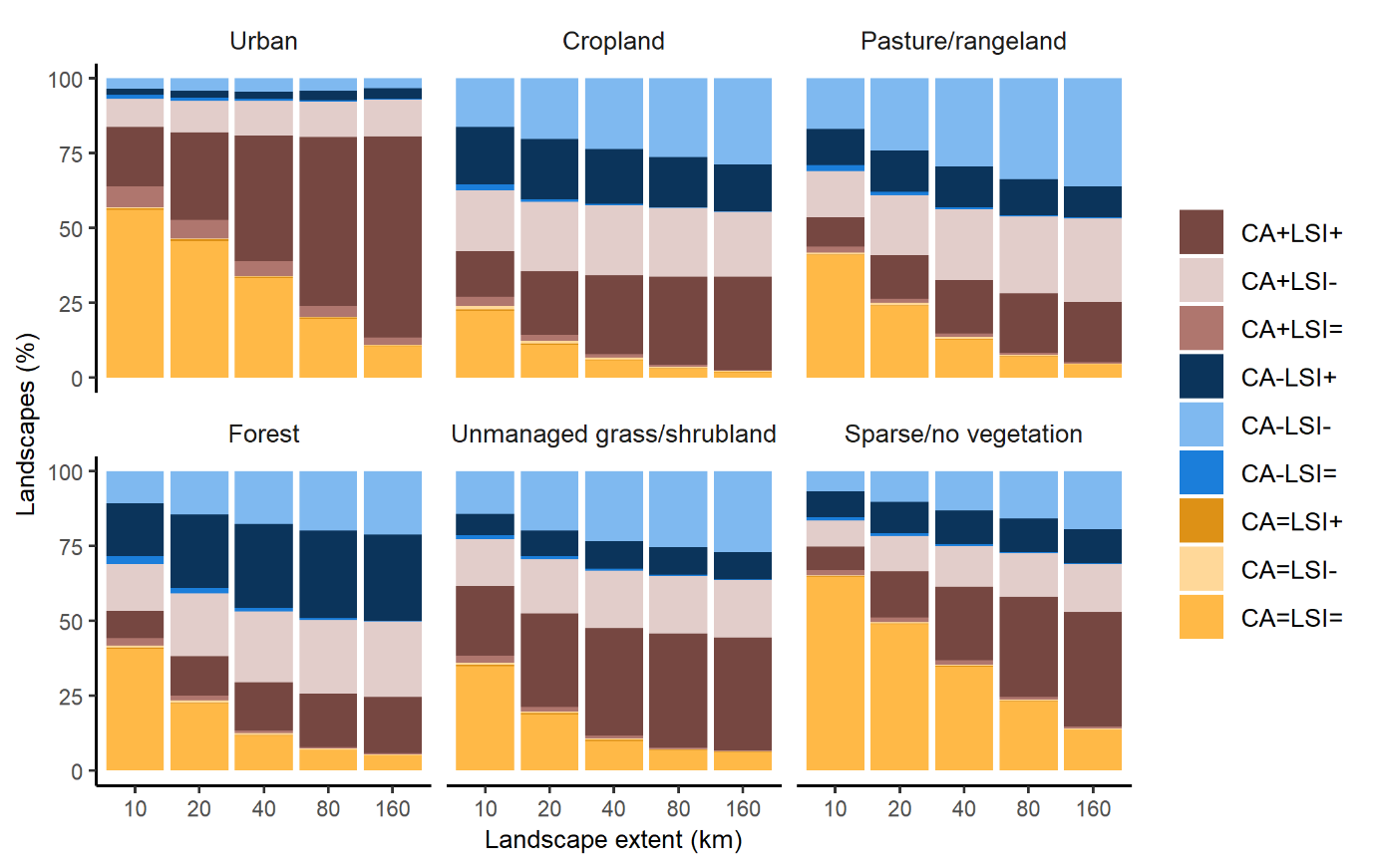


**Fig. S 24** Direction of net change in area and configuration within landscapes of increasing extent from 1992 to 2020. Landscape extent is the length of each side of a landscape in kilometres. Bars give the percentage of landscapes which contained the specific land use and land cover (LULC) class in both 1992 and 2020


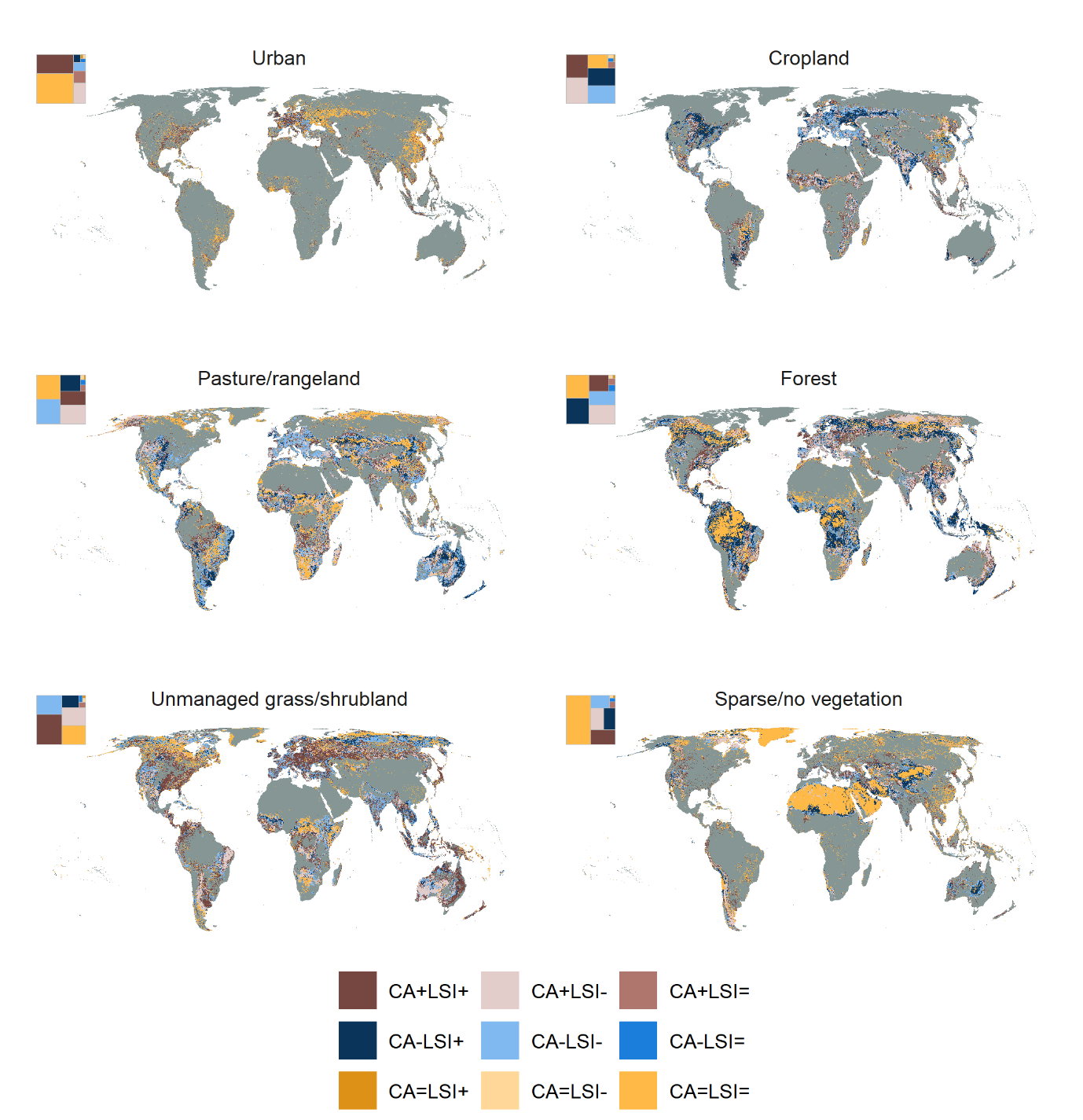


**Fig. S 25** Direction of net change in area and configuration in 400 km^2^ landscapes from 1992 to 2020. Direction of net change is shown for landscapes which contained the land use and land cover (LULC) class of interest in both 1992 and 2020. CA = class area, LSI = Landscape Shape Index. The square inset in each panel shows the relative proportion of landscapes assigned to each of the nine categories of CA and LSI change for that LULC class. Grey shading indicates the absence of a LULC class in a landscape in both 1992 and 2020. Note that LSI+ indicates increased fragmentation per se of a LULC class and LSI- represents decreased fragmentation per se


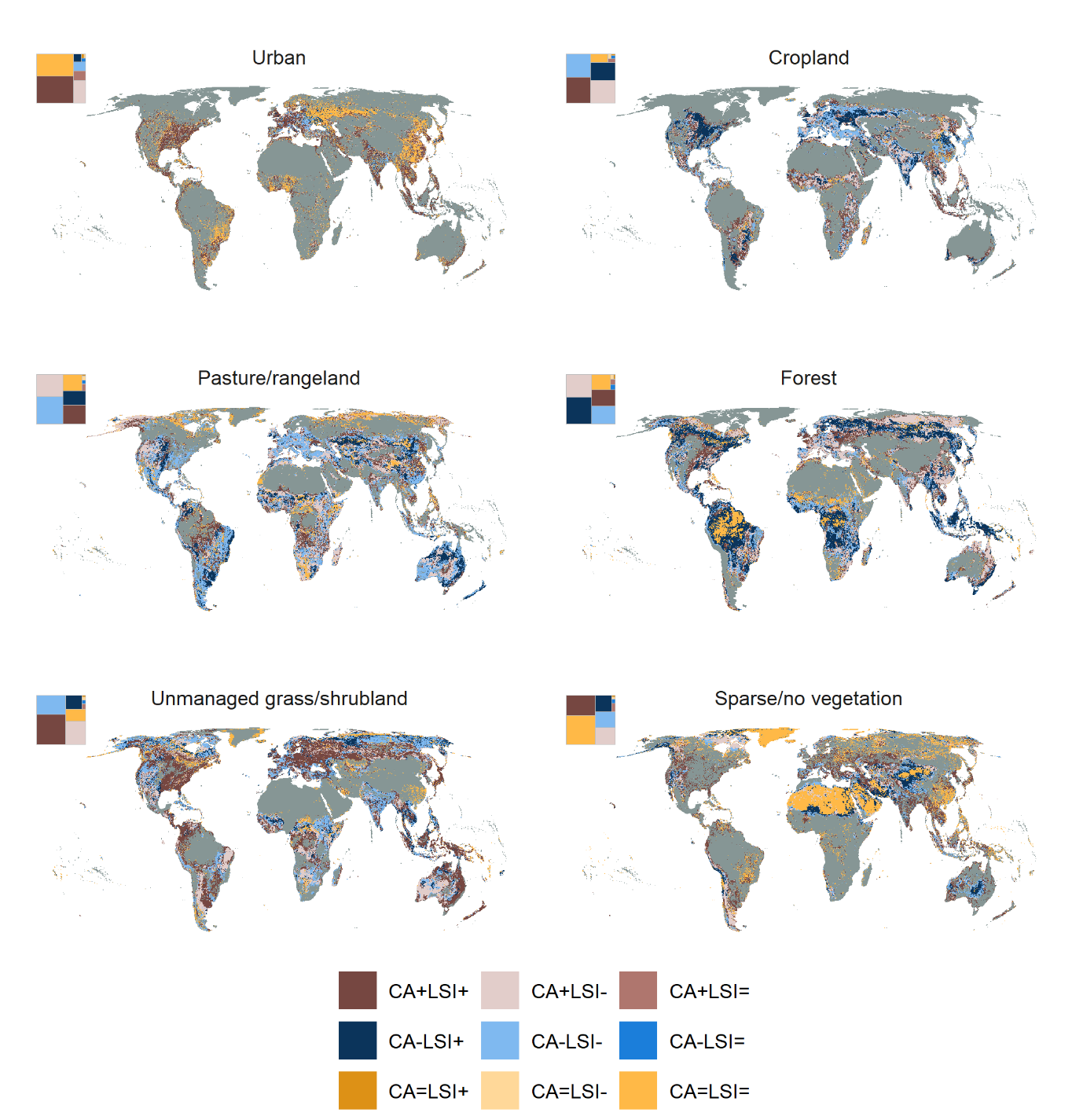


***Fig. S 26*** *Direction of net change in area and configuration in 1600 km^2^ landscapes from 1992 to 2020. Direction of net change is shown for landscapes which contained the land use and land cover (LULC) class of interest in both 1992 and 2020. CA = class area, LSI = Landscape Shape Index. The square inset in each panel shows the relative proportion of landscapes assigned to each of the nine categories of CA and LSI change for that LULC class. Grey shading indicates the absence of a LULC class in a landscape in both 1992 and 2020. Note that LSI+ indicates increased fragmentation per se of a LULC class and LSI- represents decreased fragmentation per se*


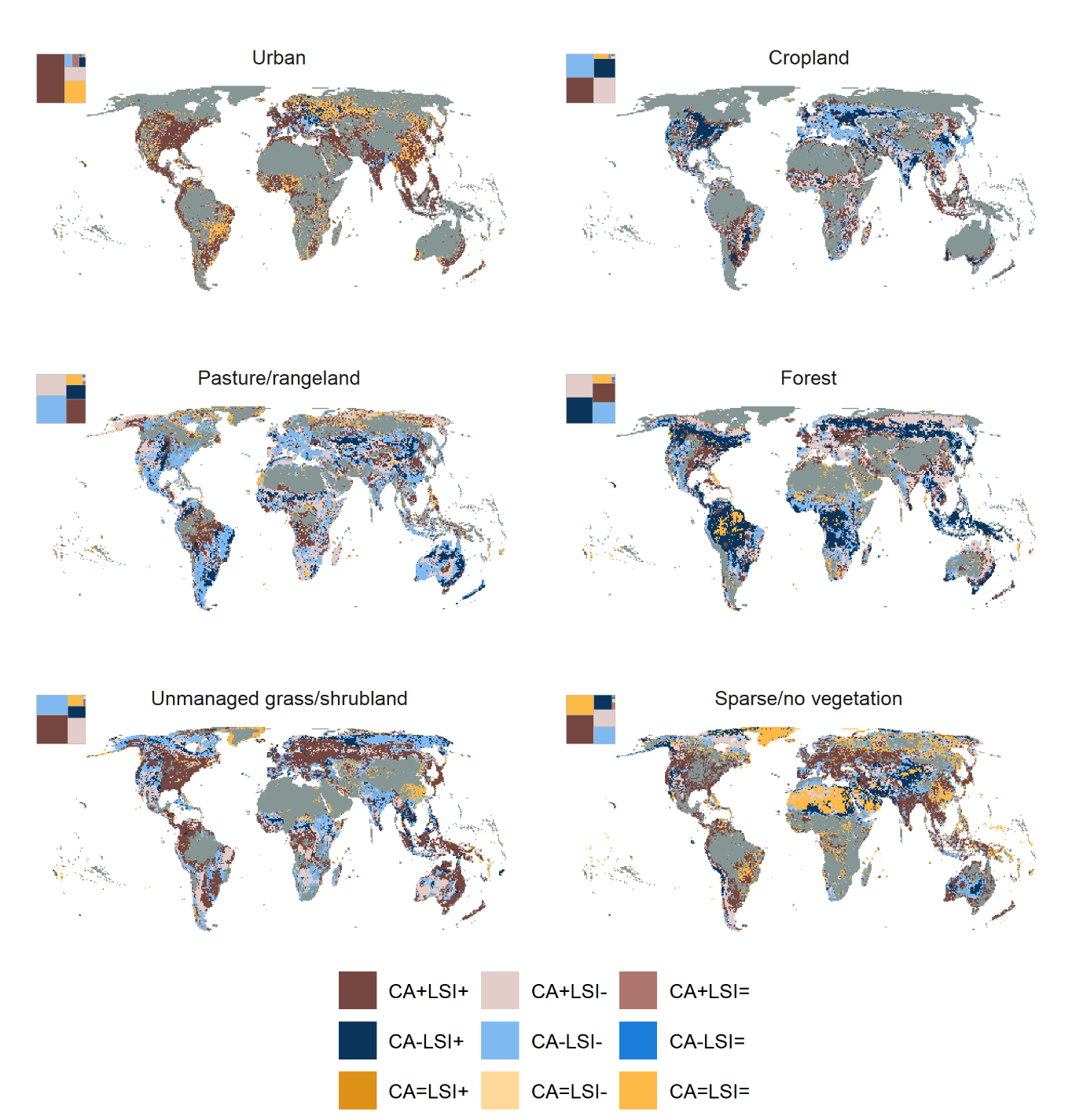


**Fig. S 27** Direction of net change in area and configuration in 6400 km^2^ landscapes from 1992 to 2020. Direction of net change is shown for landscapes which contained the land use and land cover (LULC) class of interest in both 1992 and 2020. CA = class area, LSI = Landscape Shape Index. The square inset in each panel shows the relative proportion of landscapes assigned to each of the nine categories of CA and LSI change for that LULC class. Grey shading indicates the absence of a LULC class in a landscape in both 1992 and 2020. Note that LSI+ indicates increased fragmentation per se of a LULC class and LSI- represents decreased fragmentation per se


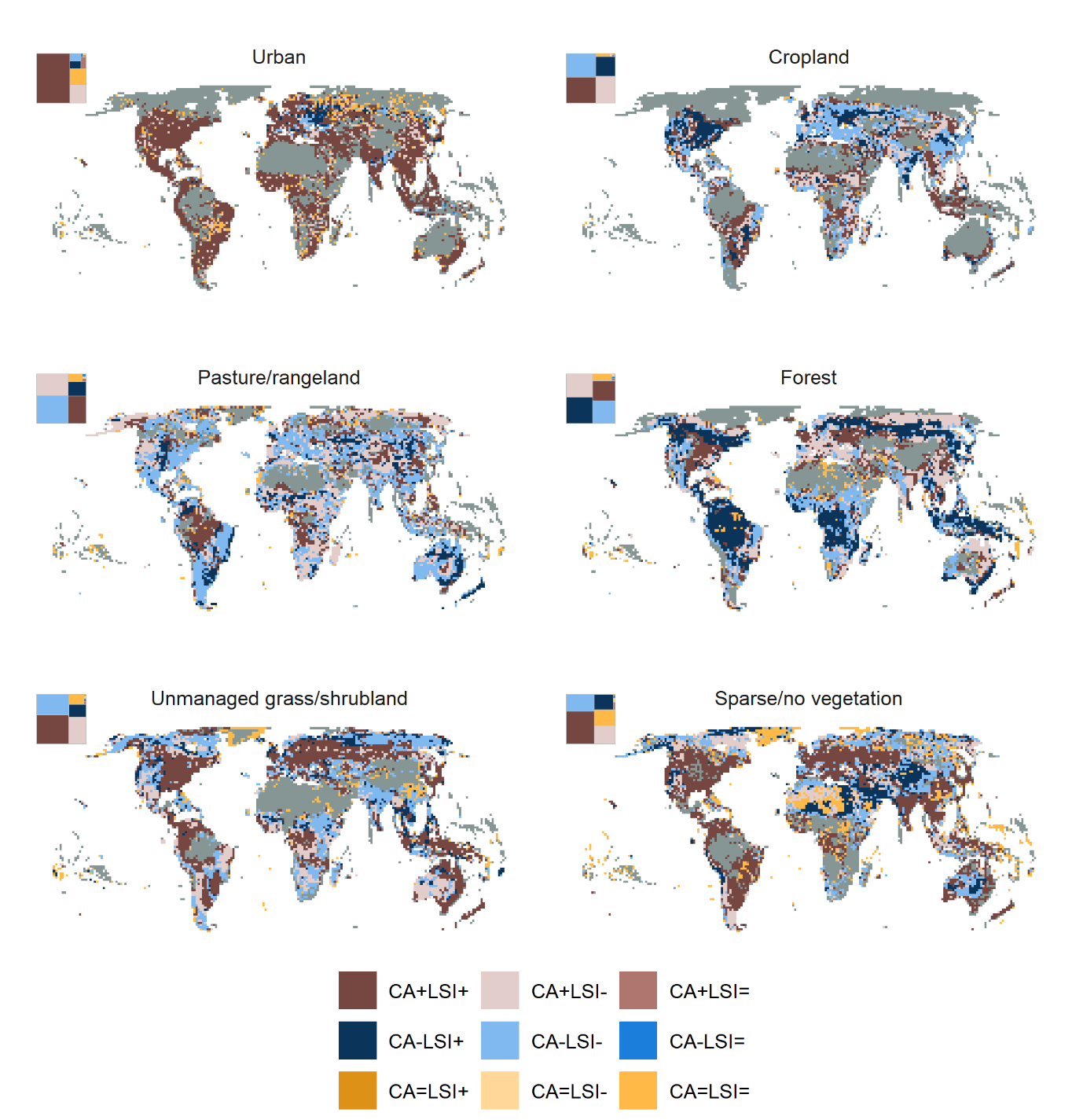


***Fig. S 28*** *Direction of net change in area and configuration in 25600 km^2^ landscapes from 1992 to 2020. Direction of net change is shown for landscapes which contained the land use and land cover (LULC) class of interest in both 1992 and 2020. CA = class area, LSI = Landscape Shape Index. The square inset in each panel shows the relative proportion of landscapes assigned to each of the nine categories of CA and LSI change for that LULC class. Grey shading indicates the absence of a LULC class in a landscape in both 1992 and 2020. Note that LSI+ indicates increased fragmentation per se of a LULC class and LSI- represents decreased fragmentation per se*


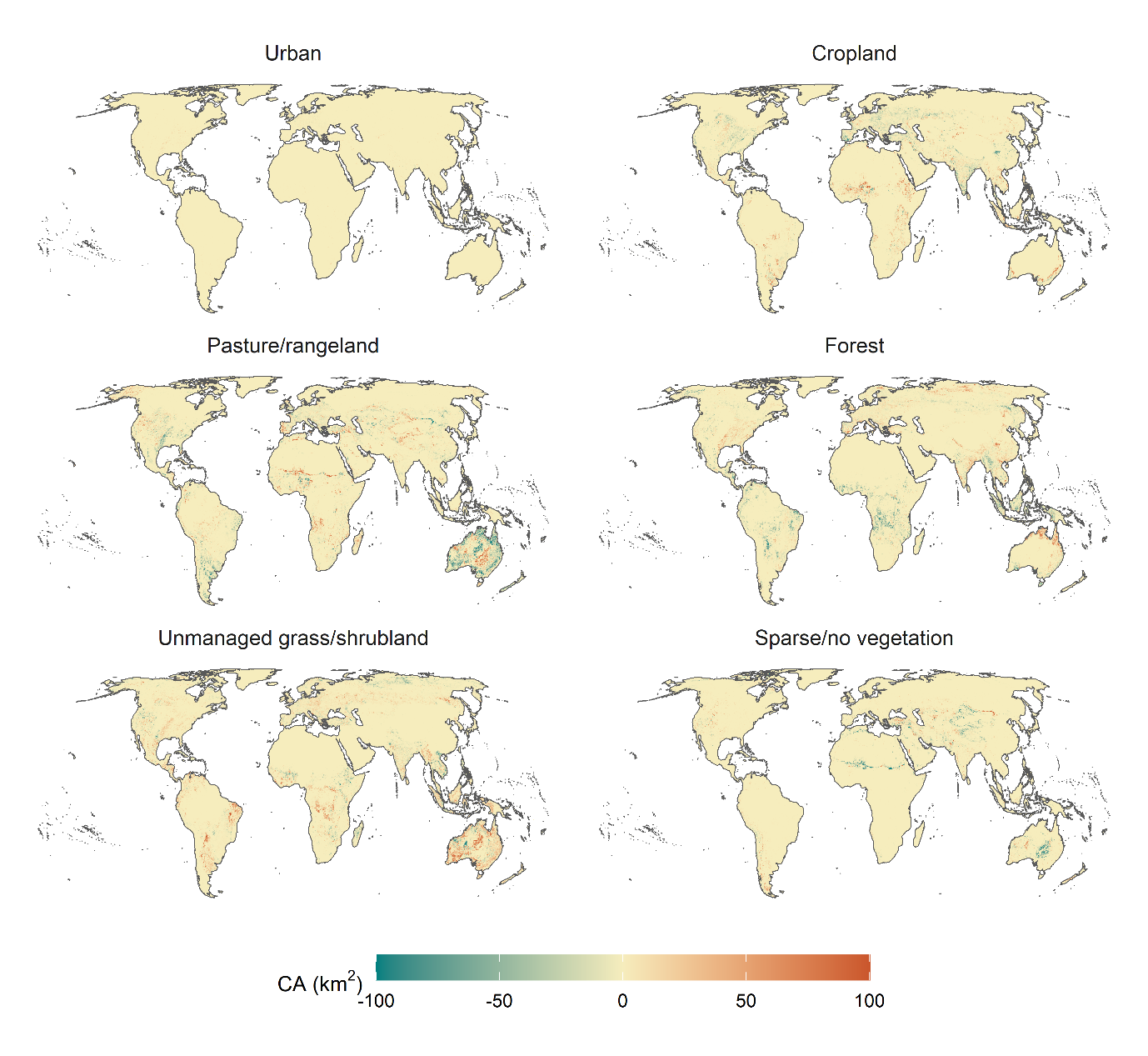


**Fig. S 29** Net change in class area (CA) from 1992 to 2020. Net change is shown in 100 km^2^ extent landscapes at global scale for six land use and land cover classes. Positive values (red) indicate a net increase in CA by 2020, whereas negative values (teal) indicate a net decrease. Units for CA are square kilometres (km^2^)


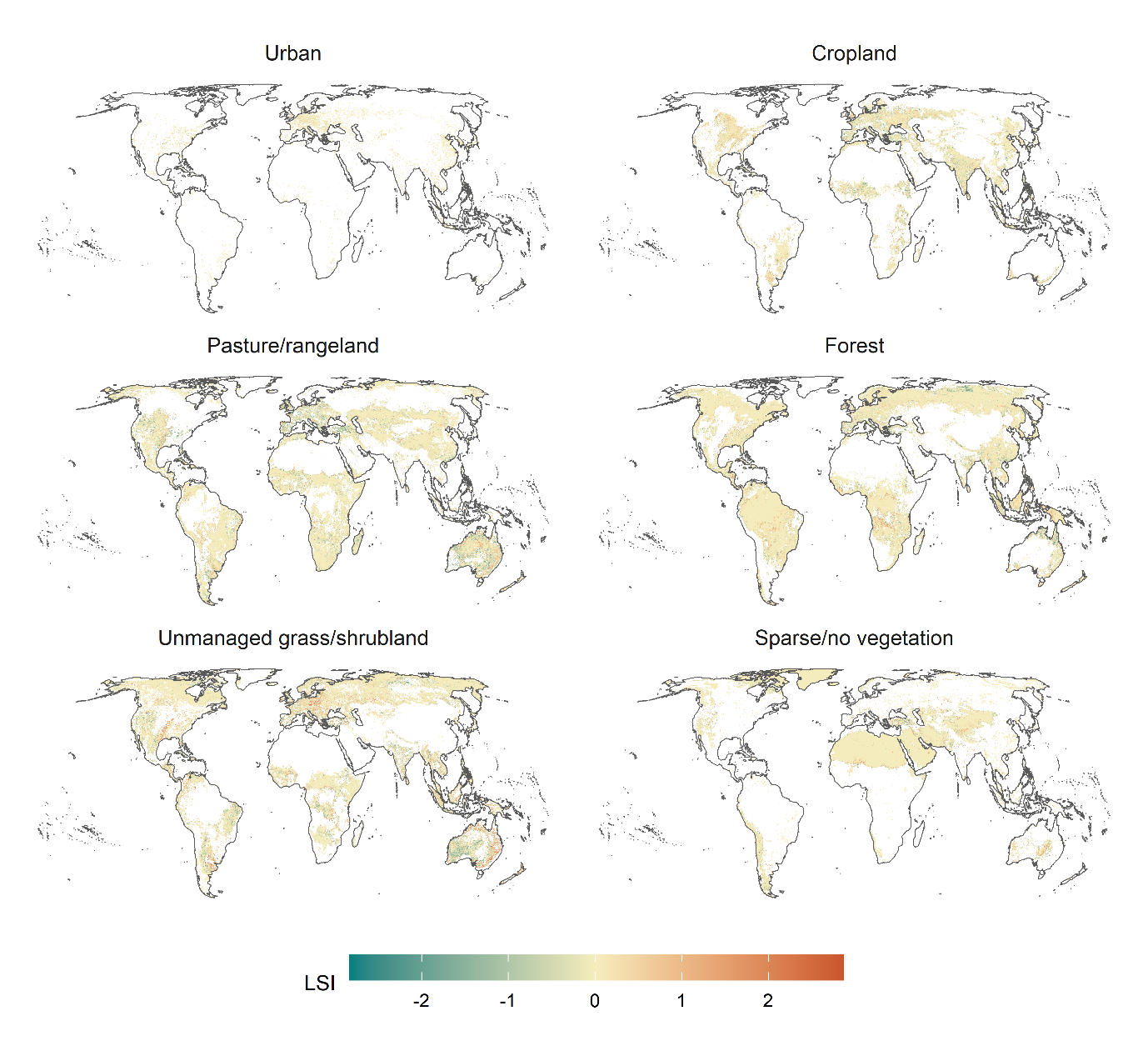


**Fig. S 30** Net change in Landscape Shape Index (LSI) from 1992 to 2020. It was not possible to calculate LSI when a land use and land cover (LULC) class was missing from a landscape, hence net change in LSI is only plotted for landscapes that contained that LULC class in both 1992 and 2020. See Fig. S 29 for further information. LSI is a unitless measure


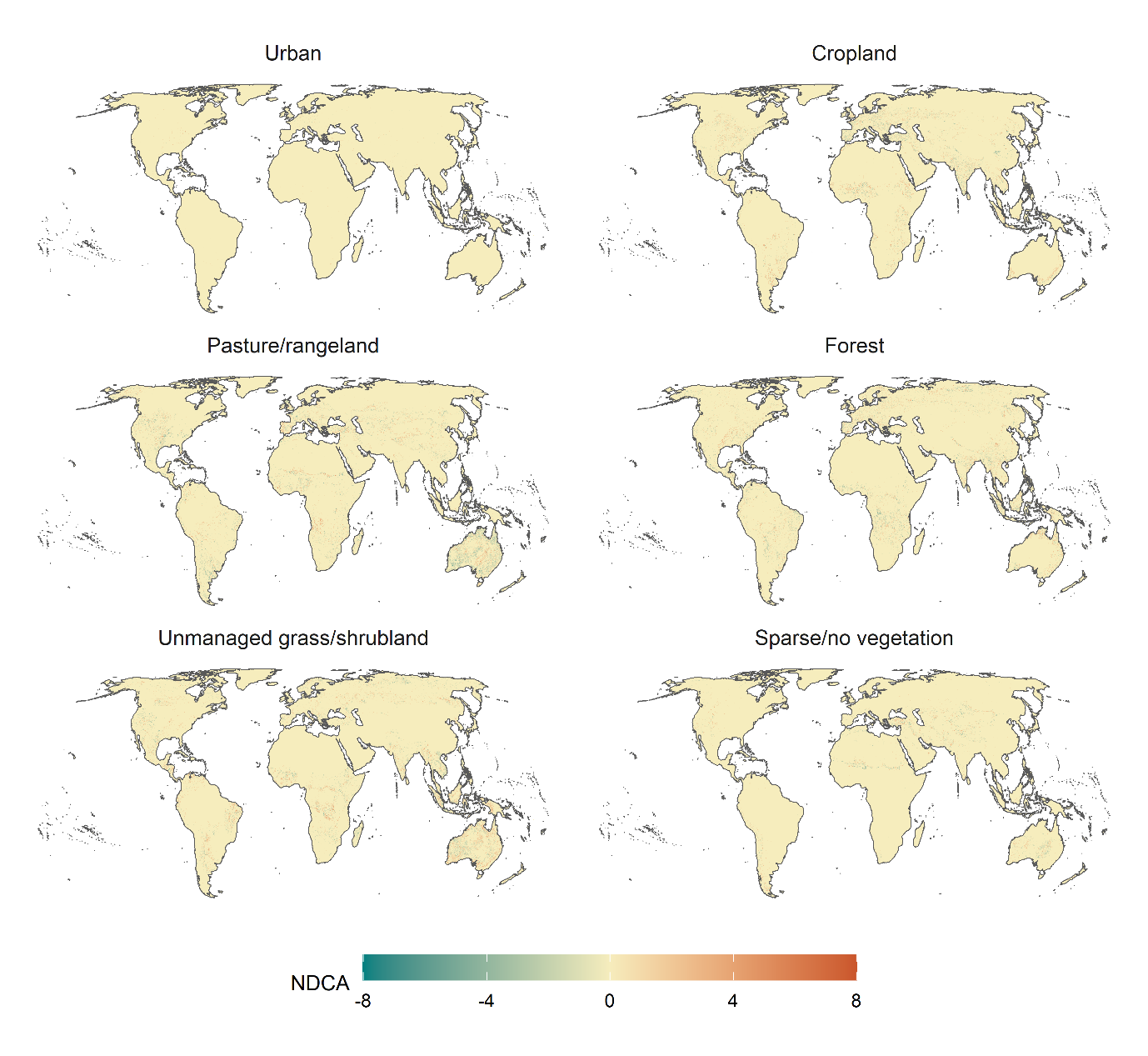


**Fig. S 31** Net change in number of disjunct core area patches (NDCA) from 1992 to 2020. See Fig. S 29 for further information. NDCA is a unitless measure


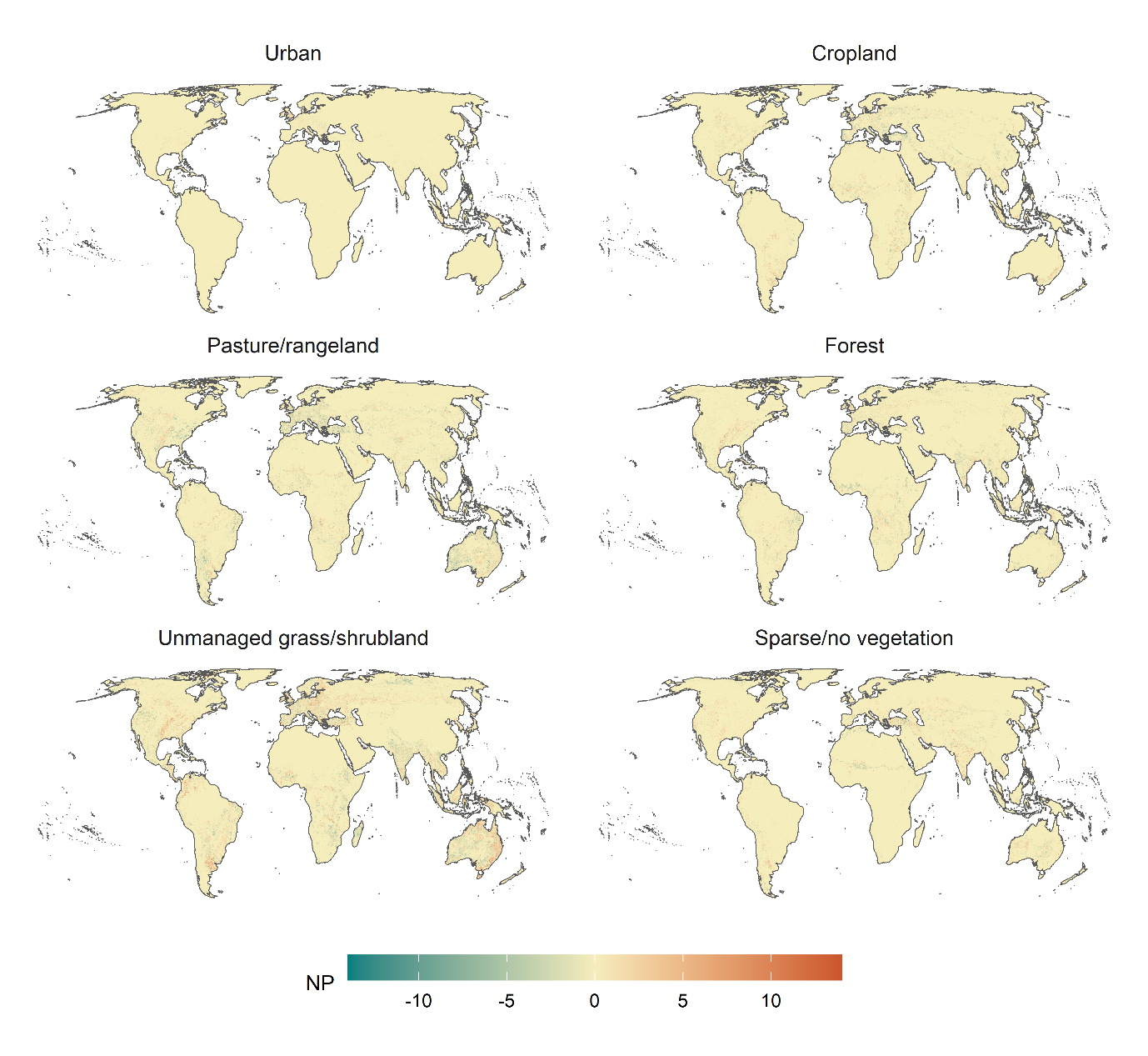


**Fig. S 32** Net change in number of patches (NP) from 1992 to 2020. See Fig. S 29 for further information. NP is a unitless measure


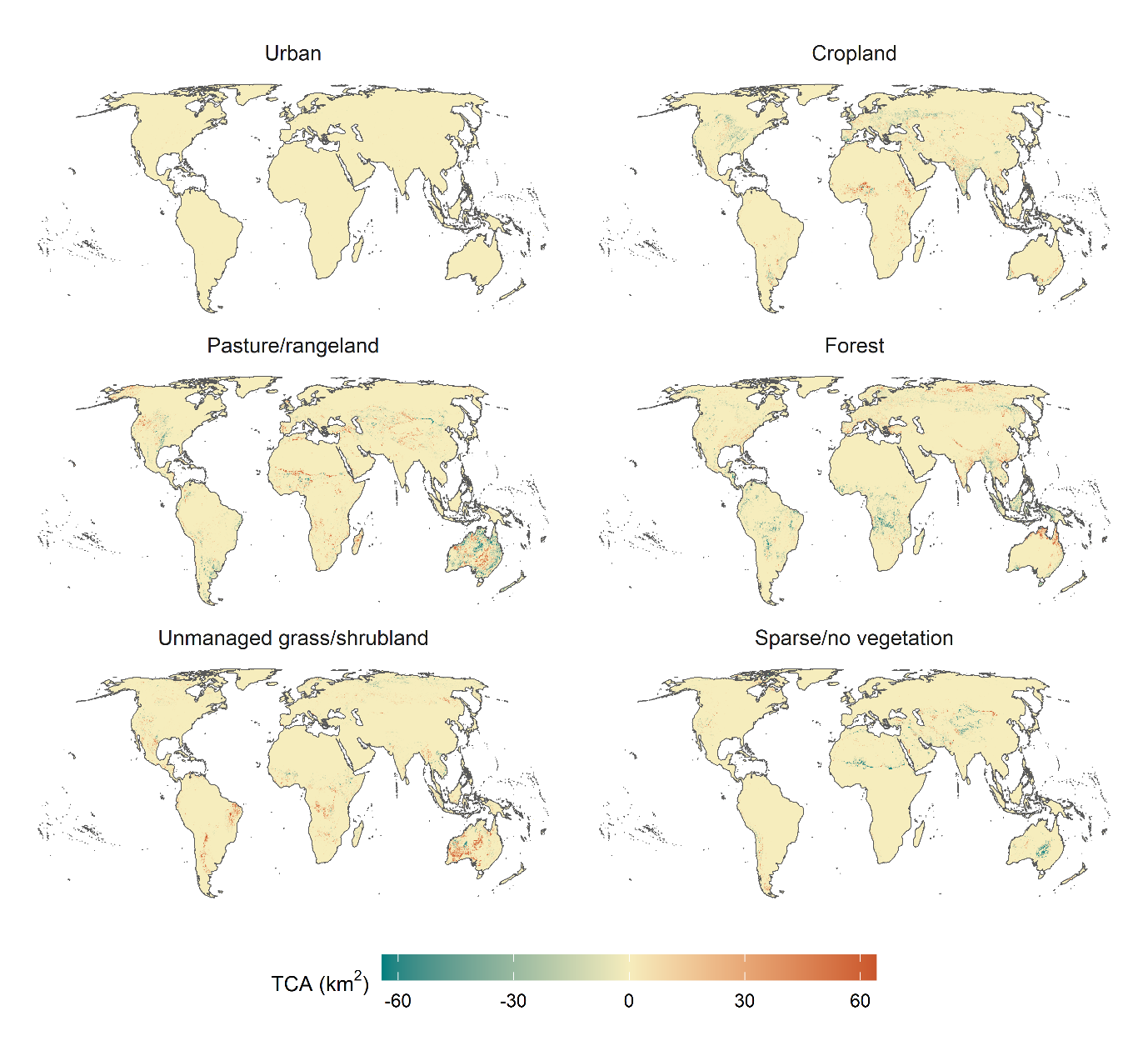


**Fig. S 33** Net change in total core area (TCA) from 1992 to 2020. See Fig. S 29 for further information. TCA is in units of square kilometres (km^2^)


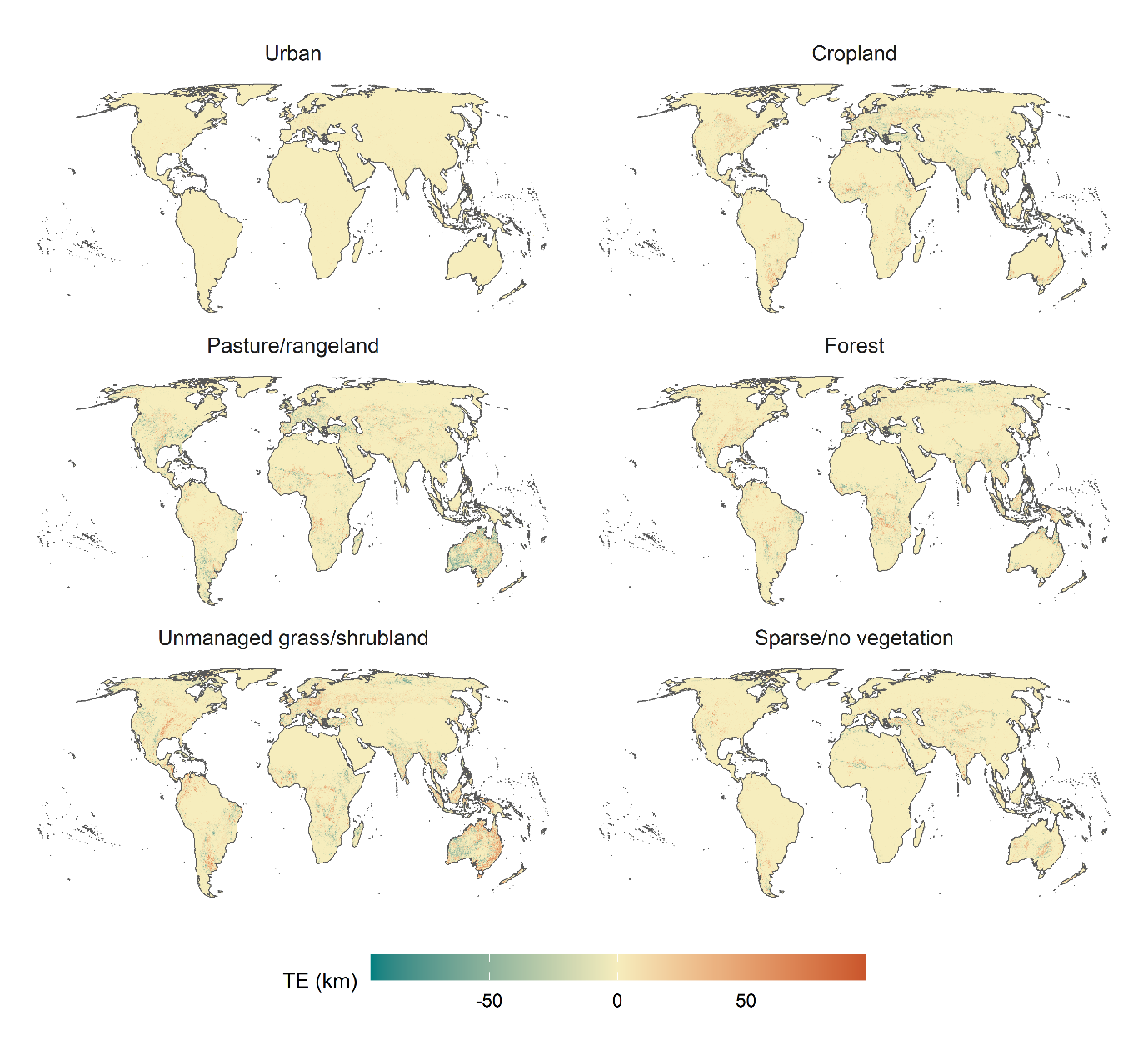


**Fig. S 34** Net change in total edge length (TE) from 1992 to 2020. See Fig. S 29 for further information. TE is displayed in units of kilometres (km)

## References

Hesselbarth MHK, Sciaini M, With KA, et al (2019) landscapemetrics: an open-source R tool to calculate landscape metrics. Ecography 42:1648–1657. https://doi.org/10.1111/ecog.04617
